# Expert consensus statement on the science of HIV in the context of criminal law

**DOI:** 10.1002/jia2.25161

**Published:** 2018-07-25

**Authors:** Françoise Barré‐Sinoussi, Salim S Abdool Karim, Jan Albert, Linda‐Gail Bekker, Chris Beyrer, Pedro Cahn, Alexandra Calmy, Beatriz Grinsztejn, Andrew Grulich, Adeeba Kamarulzaman, Nagalingeswaran Kumarasamy, Mona R Loutfy, Kamal M El Filali, Souleymane Mboup, Julio SG Montaner, Paula Munderi, Vadim Pokrovsky, Anne‐Mieke Vandamme, Benjamin Young, Peter Godfrey‐Faussett

**Affiliations:** ^1^ Pasteur Institute Paris France; ^2^ Mailman School of Public Health Columbia University New York NY USA; ^3^ Centre for the AIDS Program of Research in South Africa University of KwaZulu‐Natal Durban South Africa; ^4^ Weill Medical College Cornell University New York NY USA; ^5^ Department of Microbiology, Tumor and Cell Biology Karolinska Institutet Stockholm Sweden; ^6^ Institute of Infectious Disease and Molecular Medicine University of Cape Town Cape Town South Africa; ^7^ Department of Epidemiology Center for AIDS Research and Center for Public Health and Human Rights John Hopkins Bloomberg School of Public Health Baltimore MD USA; ^8^ Infectious Diseases Unit Juan A. Fernandez Hospital Buenos Aires CABA Argentina; ^9^ Buenos Aires University Medical School Buenos Aires Argentina; ^10^ Fundación Huésped Buenos Aires Argentina; ^11^ Infectious Diseases Geneva University Hospital Geneva Switzerland; ^12^ Instituto Nacional de Infectologia Evandro Chagas‐Fiocruz Fiocruz, Rio de Janeiro Brazil; ^13^ Kirby Institute University of New South Wales Sydney NSW Australia; ^14^ Faculty of Medicine University of Malaya Kuala Lumpur Malaysia; ^15^ YRGCARE Medical Centre Voluntary Health Services Chennai India; ^16^ Women's College Research Institute Toronto Canada; ^17^ Women's College Hospital Toronto Canada; ^18^ Department of Medicine University of Toronto Toronto Canada; ^19^ Infectious Diseases Unit Ibn Rochd Universtiy Hospital Casablanca Morocco; ^20^ Institut de Recherche en Santé de Surveillance Epidemiologique et de Formations Dakar Senegal; ^21^ Faculty of Medicine University of British Columbia Vancouver Canada; ^22^ BC Centre for Excellence in HIV/AIDS Vancouver Canada; ^23^ International Association of Providers of AIDS Care Kampala Uganda; ^24^ Russian Peoples’ Friendship University (RUDN‐ University) Moscow Russian Federation; ^25^ Central Research Institute of Epidemiology Federal Service on Customers’ Rights Protection and Human Well‐being Surveillance Moscow Russian Federation; ^26^ KU Leuven Department of Microbiology and Immunology Rega Institute for Medical Research, Clinical and Epidemiological Virology Leuven Belgium; ^27^ Center for Global Health and Tropical Medicine Unidade de Microbiologia Instituto de Higiene e Medicina Tropical Universidade Nova de Lisboa Lisbon Portugal; ^28^ International Association of Providers of AIDS Care Washington DC USA; ^29^ UNAIDS Geneva Switzerland; ^30^ Department of Infectious and Tropical Diseases London School of Hygiene and Tropical Medicine London England

**Keywords:** human rights, law and policy, risk factors, policy, criminalization, criminal law, prosecution

## Abstract

**Introduction:**

Globally, prosecutions for non‐disclosure, exposure or transmission of HIV frequently relate to sexual activity, biting, or spitting. This includes instances in which no harm was intended, HIV transmission did not occur, and HIV transmission was extremely unlikely or not possible. This suggests prosecutions are not always guided by the best available scientific and medical evidence.

**Discussion:**

Twenty scientists from regions across the world developed this Expert Consensus Statement to address the use of HIV science by the criminal justice system. A detailed analysis of the best available scientific and medical research data on HIV transmission, treatment effectiveness and forensic phylogenetic evidence was performed and described so it may be better understood in criminal law contexts. Description of the possibility of HIV transmission was limited to acts most often at issue in criminal cases. The possibility of HIV transmission during a single, specific act was positioned along a continuum of risk, noting that the possibility of HIV transmission varies according to a range of intersecting factors including viral load, condom use, and other risk reduction practices. Current evidence suggests the possibility of HIV transmission during a single episode of sex, biting or spitting ranges from no possibility to low possibility. Further research considered the positive health impact of modern antiretroviral therapies that have improved the life expectancy of most people living with HIV to a point similar to their HIV‐negative counterparts, transforming HIV infection into a chronic, manageable health condition. Lastly, consideration of the use of scientific evidence in court found that phylogenetic analysis alone cannot prove beyond reasonable doubt that one person infected another although it can be used to exonerate a defendant.

**Conclusions:**

The application of up‐to‐date scientific evidence in criminal cases has the potential to limit unjust prosecutions and convictions. The authors recommend that caution be exercised when considering prosecution, and encourage governments and those working in legal and judicial systems to pay close attention to the significant advances in HIV science that have occurred over the last three decades to ensure current scientific knowledge informs application of the law in cases related to HIV.

## 1 Introduction

At least 68 countries have laws that specifically criminalize HIV non‐disclosure, exposure, or transmission. Thirty‐three countries are known to have applied other criminal law provisions in similar cases (Unpublished data, HIV Justice Network, 2018). Most prosecutions have related to perceived risk of HIV acquisition associated with sexual activity but prosecutions have also occurred for acts such as biting and spitting (Unpublished data, HIV Justice Network, 2018). These laws and prosecutions have not always been guided by the best available scientific and medical evidence 1, have not evolved to reflect advancements in knowledge of HIV and its treatment, and can be influenced by persistent societal stigma and fear associated with HIV 2. HIV continues to be singled out, with prosecutions occurring in cases where no harm was intended; where HIV transmission did not occur, was not possible or was extremely unlikely; and where transmission was neither alleged nor proven 1, 3.

In this context, 20 HIV scientists with expertise in scientific research, epidemiology, and patient care from regions across the world developed this Consensus Statement, prompted by concern that criminal law is sometimes applied in a manner inconsistent with contemporary medical and scientific evidence: including overstating both the risk of HIV transmission and also the potential for harm to a person's health and wellbeing. Such limited understanding of current HIV science reinforces stigma and may lead to miscarriages of justice. It may also undermine efforts to address the HIV epidemic 4. The Consensus Statement has been endorsed by additional scientists from across the globe (See Supplementary Material S1), and by the International AIDS Society, the International Association of Providers of AIDS Care and the Joint United Nations Programme on HIV/AIDS. An Executive Summary of this Statement is included as Supplementary Material S2.

This Consensus Statement aims to assist scientific experts considering individual criminal cases in which HIV non‐disclosure, (perceived or possible) exposure, or transmission has been alleged. It provides expert opinion regarding individual HIV transmission dynamics (i.e. the “possibility” of transmission), long‐term impact of chronic HIV infection (i.e. the “harm” of HIV), and the application of phylogenetic analysis as evidence. It describes the possibility of HIV transmission between individuals who have engaged in a specific act at a specific time under specific circumstance, as that is usually the focus of criminal cases, and aims to communicate current scientific evidence relating to HIV in a manner understandable to a non‐scientific audience. The Consensus Statement has been translated into French, Russian and Spanish (See Supplementary Material [Link], [Link]–S5).

## 2 Discussion

The first part of this Statement focuses on the possibility of HIV transmission during specific acts that are commonly considered in prosecutions: sexual activity, biting or spitting 3. It does not reference other ways HIV may be transmitted, for example, through blood transfusion, needle stick injury, injecting drugs or breastfeeding.

An initial meeting in Seattle (February 2017) decided the contents and framing of this Consensus Statement. A detailed literature review was prepared based on a search for literature published in English using the PubMed online database up to April 2017. Specific search terms relating to the possibility of HIV transmission were used, including “HIV and viral load,” “HIV sexual transmission risk per act,” “oral sex HIV transmission,” “anal sex HIV transmission,” “vaginal sex HIV transmission condom per act,” “anal sex HIV transmission condom per act,” and “anal sex HIV transmission circumcision per act.” Key articles were used to search for related articles. Preference was given to meta‐analyses, reviews and important studies. Other sources were identified by the expert authors. Abstracts from scientific conferences were used as appropriate.

The authors next engaged in multiple rounds of drafting and review, considering the best available scientific and medical research data according to the following hierarchy: systematic review of randomized clinical trials; randomized clinical trials; and comparative studies (i.e. cohort studies, case–control studies and historical control studies). Two teleconferences were held to discuss a preliminary draft, followed by three rounds of redrafting via electronic correspondence by all authors. National and international legal experts, including UNAIDS staff members, were consulted on the application of the criminal law in cases involving HIV. A second face‐to‐face meeting was convened in Paris (July 2017) to resolve outstanding data analysis issues. Further rounds of comment and redrafting were undertaken by the authors to ensure agreement that the Consensus Statement accurately relayed current scientific research related to HIV transmission, harms and the use of scientific evidence in court.

The authors considered numerical findings and statistical estimates from all studies cited herein, including data summaries from reports presented in systematic or table form (for example, the works of Patel *et al*. 5). Evidence establishing estimates of the possibility of HIV transmission through different acts varies in both type and quality; the authors factored these considerations into their assessment of the possibility associated with different acts. The authors considered that the evidence regarding transmission via different acts falls into three categories (Table 1).

**Table 1:** Quality scale for evidence regarding the possibility of HIV transmission

Specific acts	Examples
Acts for which the transmission possibility can be estimated with some degree of certainty because multiple cohort studies have been undertaken.	Acts such as vaginal or anal sex.
Acts for which transmission possibility can be estimated with less certainty from isolated case reports, biological plausibility or mathematical models.	Acts such as oral sex or transmission via pre‐ejaculate fluid.
Acts for which it is biologically implausible for transmission to occur as the conditions required for transmission are not present.	Acts such as spitting.

When describing the evidence, the authors aimed to use scientific concepts in ways that are helpful in the context of criminal law. For example, the statistical concept of confidence intervals is designed to address uncertainty inherent in results derived from sampling a subset of a population. When dealing with probabilities that are or approach zero, confidence intervals take on special significance because the fact that something was not observed to happen during a study cannot prove that it could never happen. The larger the study, the more precisely the authors can estimate that the probability is zero. Consequently, a zero probability calculated from study data is associated with a confidence interval from zero to a small, positive probability. It is important that calculations of confidence intervals are not misinterpreted to exaggerate remote theoretical possibilities.

Consideration of the methodology and results of studies cited in this Consensus Statement informed the development of three descriptors located along a continuum to describe the possibility of HIV transmission during a single, specific act (Table 2).

**Table 2:** Defining the possibility of HIV transmission during a single, specific act

Terminology for this statement	Possibility of transmission per act
Low possibility	Transmission during a single act is possible but the likelihood is low.
Negligible possibility	Transmission during a single act is extremely unlikely, rare or remote.
No possibility	The possibility of transmission during a single act is either biologically implausible or effectively zero.

Importantly, this Consensus Statement is not intended as a public health document to inform HIV prevention, treatment and care messaging or programming. Its approach, based on individual‐level risk which may be applied in criminal justice settings, differs from descriptions of population‐level risks that are used in the context of public health, which often describe sexual acts as ranging from “low risk” to “high risk.” The differences between the public health descriptors and those used in this Consensus Statement reflect both history and context. First, public health definitions used to describe HIV transmission risk were developed during the early days of the HIV epidemic, before the emergence of recent evidence on HIV transmission. Second, they describe relative risk (not absolute risk) as a means to help people reduce the possibility of HIV transmission by comparing different acts.

Although the simplicity of such public health terminology was originally intended to support effective, broad‐based public health education campaigns for HIV prevention, its generalized categories now pose real problems for those developing current HIV health promotion messaging based on up‐to‐date scientific evidence 6, including evidence of the different variables that modify risk associated with specific acts, such as viral load. In some instances, understanding of the riskiness of certain sexual acts communicated by public health characterizations has also been misapplied in the context of criminal proceedings, for example, the Canadian case of Mabior 7, 8. Consequently, although sexual transmission is a common form of HIV transmission at a global population level, this Consensus Statement recognizes that the possibility of HIV transmission during a single sexual encounter ranges from no possibility to low possibility, while it ranges from no possibility to negligible possibility in cases of spitting or biting. This approach to the science of HIV in the context of criminal law is similar to that used in national scientific consensus statements from Australia 9, Canada 10, Sweden 11 and Switzerland 12.

### 2.1 Possibility of transmission: overview

HIV is not easily transmitted from one person to another. It is a relatively fragile virus that is transmitted through specific well‐described routes. It is not passed on through airborne, droplet, fomite, contact or vector‐borne transmission routes and cannot penetrate intact human skin 13.

For HIV transmission to occur, certain basic conditions must exist:

○ There must be a sufficient amount of the virus in particular bodily fluids (i.e. blood, semen, pre‐seminal fluid, rectal fluids, vaginal fluids, or breast milk).
○ A sufficient quantity of at least one of those bodily fluids must come into direct contact with sites in the body of an HIV‐negative person where infection can be initiated. These are usually mucous membranes, damaged tissue or inflamed ulcers, but not intact skin.
○ The virus must overcome the person's innate immune defences so that infection can be established and propagated.

Most everyday activities carry no risk of HIV transmission because these conditions are not met. Leaving aside parenteral or vertical transmission, intimate contact, such as sexual intercourse, is usually required for transmission. Even in those cases, the per‐act chance of transmission is zero to low (with estimates ranging from 0% to 1.4% per act) 5.

### 2.2 Factors influencing the possibility of HIV transmission

The possibility of HIV transmission associated with individual acts varies according to a range of intersecting factors. When multiple intersecting factors are present, their effect is minimized or amplified to various degrees 14.

- Correct use of a condom prevents HIV transmission

Correct use of a condom (either male or female) prevents HIV transmission because the porosity of condoms is protective against even the smallest sexually transmissible pathogens, including HIV 15; latex and polyurethane condoms act as an impermeable physical barrier through which HIV cannot pass. Correct condom use means the integrity of the condom is not compromised and the condom is worn throughout the sex act in question. Correct use of a condom during sex means HIV transmission is not possible.

Population level studies have found that consistent condom use for anal or vaginal sex dramatically reduces the possibility of HIV transmission even when factoring in instances of incorrect use or breakage 16, 17, 18, 19, 20, 21. For example, a meta‐analysis of 14 studies found that long periods of consistent use of male condoms during vaginal sex reduces the possibility of HIV transmission by at least 80% 22. However, more recent research suggests that this may be an underestimate 23, with the meta‐analysis described including non‐standard data analysis methods which may have led to recruitment and other biases which could have lowered the level of prevention observed 22, 23.

Population‐level research is only relevant in cases where multiple sex acts have occurred and it is not known whether condoms were correctly used in each instance. The population level estimate of 80% condom effectiveness does not exist as a stand‐alone estimate of HIV transmission risk but must be applied against risk associated with different sex acts. For example, if the estimated risk of HIV transmission from an HIV‐positive man to a woman during a single episode of condomless vaginal sex is 0.08% 5, then the risk of transmission when a condom is used can be understood as *at least* 80% lower, or 0.016% (less than 2 in 10,000) 5. Importantly, when other risk reduction factors are present (e.g. low viral load or withdrawal before ejaculation) the possibility of HIV transmission, even in the event of incorrect condom use, is further reduced.

To reiterate, HIV cannot be transmitted in individual cases where a condom has been used correctly (i.e. it was worn through the sex act in question and its integrity was not compromised). The population‐level estimates can only apply in situations where multiple instances of condom use have occurred, including occasional instances of incorrect use and breakage.

- Viral load that is low or “undetectable” significantly decreases or eliminates the possibility of HIV transmission

Soon after acquiring HIV, a person's viral load is very high but typically decreases over the first few weeks as their immune system responds. If a person does not commence treatment, their viral load remains fairly stable for some time, while the immune system is gradually depleted. In advanced HIV infection, viral load usually increases to higher levels again.

Antiretroviral therapy prevents HIV from replicating, thereby significantly reducing the viral load in a person's bodily fluids. When effective antiretroviral therapy is commenced, viral load usually drops to levels that are undetectable by current standard laboratory blood tests within a few weeks or months. Testing availability and lower limits of detection vary in different parts of the world, with lower limits of detection ranging from around 20 viral copies/mL to 400 copies/mL. A small percentage of people living with HIV (often referred to as long‐term non‐progressors) have a low viral load without taking antiretroviral therapy because their immune systems are able to control HIV 24, 25, 26, 27, 28.

Reduced viral load improves immune function and dramatically decreases the long‐term likelihood of illness and death. It also greatly reduces the possibility of HIV transmission 29, 30, 31. Decreases in viral load are associated with concomitant decreases in the likelihood of HIV transmission 32, 33, 34, 35, meaning that many people on treatment cannot transmit HIV.

Recent analyses from key studies (namely, HPTN052, PARTNER and Opposites Attract) involving both heterosexual and male couples of different HIV status have not identified any cases of sexual transmission from a person with an undetectable viral load 29, 30, 36, 37. These findings have transformed public health messaging. For example, the United States Centers for Disease Control and Prevention now describes the estimated possibility of HIV transmission from an HIV‐positive person with an undetectable viral load (as a result of effective antiretroviral treatment) as “effectively no risk” 6.

In 2011, the HPTN052 trial (conducted in Botswana, Brazil, India, Kenya, Malawi, South Africa, Thailand, the United States and Zimbabwe), which investigated the impact of early treatment initiation, observed no HIV transmission from 1763 people on antiretroviral therapy who had a stable viral load below 400 copies/mL. Partners of HIV‐positive participants were followed for the equivalent of 8509 person‐years. The only transmission from people on treatment occurred either early in treatment (before viral load was stabilized below 400 copies) or when viral load was above 1000 copies/mL on two consecutive visits 29, 37.

The PARTNER and Opposites Attract studies found no HIV transmission from people with a viral load below 200 copies/mL after more than 75,000 acts of condomless vaginal or anal sex 18, 30, 38. In the PARTNER study, heterosexual couples reported approximately 36,000 condomless sex acts and homosexual male couples reported about 22,000 condomless sex acts 30. No HIV transmission occurred between partners in the study. Eleven cases of new HIV infection did occur, however, phylogenetic analysis found that in all cases, the infection resulted from sexual contact with someone other than the person's regular sexual partner. The Opposites Attract study included nearly 17,000 condomless sex acts among men. No HIV transmission was reported between partners involved in the study, while three cases of new HIV infection resulted from sexual contact with someone other than the person's regular sexual partner 18.

A 2013 systematic review and meta‐analysis also found no transmission where viral load fell below a threshold of between 50 and 500 copies/mL (depending on the study) 39. Another study reported no transmission when viral load was lower than 400 copies/mL 40. A number of other studies have provided evidence that low (but detectable) viral load dramatically decreases (and may eliminate) the possibility of transmission. For example, early studies involving participants who were not taking antiretroviral therapy identified no instances of transmission among couples where one partner was living with HIV and had a low but detectable viral load: below 1500 copies/mL (Uganda) 32, below 1094 copies/mL (Thailand) 33 and below 1000 copies/mL (Zambia) 34. The Ugandan study found that the probability of transmission through vaginal intercourse where viral load was lower than 1700 copies/mL was 1 in 10,000 41.

While short‐lived, small‐magnitude increases in viral load, known as “blips,” occur among many individuals adhering to their antiretroviral therapy 42, 43, they are not an indication that HIV therapy is “failing;” are not considered to be clinically significant; and have not been shown to increase the possibility of HIV transmission during sex 44, 45. Large‐scale studies among couples of different HIV status have included many HIV‐positive participants who experienced blips in their viral load during the course of the study. Consequently, such blips have been factored into the observed reduction in transmissions.

- Pre‐exposure Prophylaxis (PrEP) significantly decreases the possibility of HIV acquisition

PrEP describes the use of antiretroviral medication by HIV‐negative people prior to HIV exposure to prevent HIV acquisition 46, 47, 48, 49, 50. One recent study has found PrEP to be up to 95% effective among adherent users 50, however, only a handful of cases of PrEP failures in adherent individuals have ever been described suggesting that it is likely that PrEP is more than 95% effective.

- Post‐exposure Prophylaxis (PEP) significantly decreases the possibility of HIV acquisition

PEP describes short‐term use of antiretroviral treatment by an HIV‐negative person after an exposure to HIV. If started within 72 hours of exposure and taken for 28 days with good adherence, PEP significantly reduces the likelihood of the person becoming HIV‐positive because it can stop HIV from establishing itself in a person's immune cells even after the virus has entered a person's body 51, 52. Although PEP is not 100% effective, high rates of success have been reported 51, 53, 54, 55, 56, 57, 58, 59, 60, 61, 62, 63, 64, 65, 66, 67 (e.g. 81% among patients using older‐style treatments 67 and up to 100% among patients using newer treatments 68). The effectiveness of PEP appears to be influenced by a number of factors, with effectiveness generally increasing the sooner PEP is commenced and as the amount of HIV entering a person's body decreases 68.

- Medical Male Circumcision decreases the possibility of HIV transmission from women to men

Medical male circumcision reduces the possibility of HIV transmission from HIV‐positive women to HIV‐negative men by approximately 50% 69. Circumcision may also decrease sexual transmission of HIV among men who have sex with men for HIV‐negative men who are exclusively the insertive partner, although studies are not conclusive 70.

- Risk reduction practices such as withdrawal or strategic positioning decrease the possibility of HIV transmission

Some people living with HIV use risk reduction practices such as withdrawal prior to ejaculation or strategic positioning (i.e. receptive‐only anal intercourse) when engaging in condomless sex with an HIV‐negative person or person of unknown serostatus 71, 72, 73. Such actions decrease the possibility of HIV transmission during sex where a possibility exists 71. For example, a 2010 study found that the likelihood of transmission during anal sex reduced by approximately two‐thirds when the HIV‐positive insertive partner did not ejaculate 73. The possibility of transmission is also known to be lower when an HIV‐positive partner is the receptive, rather than insertive, partner during anal sex 73, 74, 75.

- Sexually Transmitted Infections (STIs) can increase the possibility of HIV transmission in some circumstances

The presence of some untreated STIs, particularly ulcerative STIs, in either partner has been associated with an increased likelihood of HIV transmission during sexual activity when the person living with HIV does not have a low viral load 76. When genital ulcers are present in both partners, the risk is further increased 14. However, the presence of an STI does not increase the possibility of transmission if the HIV‐positive person is on effective antiretroviral therapy 30, *or* if the HIV‐negative person is taking PrEP 48, 49.

### 2.3 The possibility of HIV transmission through sex

HIV transmission through sex usually occurs as a result of bodily fluids containing enough HIV coming into contact with mucous membranes located in: the foreskin or urethra of the penis; the cervix or vagina; the anus; or the rectum. HIV transmission is also possible through contact with oral mucous membranes but these are much less vulnerable to HIV transmission 58.

#### 2.3.1 Oral sex, including oral‐penile sex and oral‐vaginal sex

▫ The possibility of HIV transmission from oral sex performed on an HIV‐positive person, including when the person does not have a low viral load and/or a condom is not used, varies from none to negligible depending on the context 77, 78.

Oral sex is promoted as a safer sex option for partners of different HIV status wanting to engage in intimate sexual acts, with its practice reportedly very common.

Oral sex is known to involve a much lower possibility of HIV transmission than vaginal or anal intercourse 79, 80. In fact, the risk of HIV transmission as a result of oral sex is so low that scientists have been unable to establish a statistically sound estimate.

The few clinical studies investigating transmission through oral sex have failed to find any cases of HIV transmission 74, 81, 82. A study of heterosexual couples and a study of lesbian couples found no transmission resulting from oral sex 81, 82. A third study involving men who have sex with men showed no seroconversions among participants who reported performing only fellatio (with ejaculation) on men who were HIV‐positive or of unknown HIV status 74. A statistical model applied to these findings concluded that the per‐contact risk from oral sex was between zero and 0.04% (4 in 10,000) 78 and these values are used in some reports 79, 80, 83. Given the study found no seroconversions, the upper bound of 0.04% can be understood as an upper boundary of possibility.

▫ There is no possibility of HIV transmission from oral sex performed on an HIV‐positive person when the HIV‐positive partner has a low viral load, **or** a condom is properly used, **or** the HIV‐negative partner is taking PrEP 78.

While there are no studies investigating the impact of antiretroviral therapy or PrEP on the possibility of transmission during oral sex, it is our expert opinion that there is no possibility of HIV transmission associated with oral sex performed on an HIV‐positive individual on antiretroviral therapy, or performed by a person taking PrEP. Similarly, correct condom use reduces the likelihood of HIV transmission to zero.

#### 2.3.2 Vaginal‐penile intercourse

▫ The possibility of HIV transmission from vaginal‐penile intercourse when the HIV‐positive partner does not have a low viral load **and** a condom is not used is low 84. The likelihood of transmission decreases further if no ejaculation occurs inside the HIV‐negative partner's body.

Two meta‐analyses of heterosexual couples 14, 84 found the likelihood of HIV transmission during one act of vaginal intercourse is low: 0.08% (8 in 10,000) in the absence of risk cofactors 5, 14, 41, 84. It is not clear whether the likelihood of transmitting HIV from a man to a woman during vaginal intercourse is higher than transmission from a woman to a man. Some studies have found no difference, while others suggest the possibility of HIV transmission from a man to a woman is about twice that of transmission from a woman to a man 14, 35, 83, 84.

▫ The possibility of HIV transmission from vaginal‐penile intercourse when the HIV‐positive partner has a low viral load **or** uses a condom **or** the HIV‐negative partner is taking PrEP varies from none to negligible depending on the context 29, 38.

Numerous studies, as discussed above, have shown that the possibility of HIV transmission from an HIV‐positive partner who has a low viral load during vaginal‐penile intercourse is none to negligible 29, 37, 38, 39, 85. There has not been a reported case of transmission through vaginal‐penile intercourse from a person with an undetectable viral load in any clinical trial.

HIV cannot be transmitted when a condom is used correctly because HIV cannot pass through intact latex or polyurethane. Similarly, there is no possibility of HIV transmission when a person has an undetectable viral load.

#### 2.3.3 Anal‐penile intercourse

▫ The possibility of HIV transmission when a condom is not used **and** the HIV‐positive partner does not have a low viral load is low, whether the receptive partner is male or female 86. The likelihood is lower where the HIV‐positive partner takes the receptive, rather than the insertive, role. It is also lower if the HIV‐positive insertive partner does not ejaculate inside the receptive partner.

Studies show that receptive condomless anal intercourse by heterosexual or same‐sex couples is associated with a higher likelihood of HIV transmission than receptive condomless vaginal intercourse 5, 87, 88. Individual studies have produced estimates of per‐act likelihood of HIV transmission for anal sex from 0.01% (1 in 10,000) to more than 3% (300 in 10,000) 20, 75, 84, 88, 89, 90, 91. The likelihood of transmitting from the insertive to the receptive partner is higher than the reverse 18, 75, 84.

Two systematic reviews (2010 and 2014) report a per‐act estimate of approximately 1.4% (140 in 10,000) for receptive anal sex (i.e. when the HIV‐positive person is the insertive partner) 5, 86. A 2010 prospective cohort study found that the likelihood fell from 1.43% (143 per 10,000) with ejaculation to 0.54% (54 per 10,000) with no ejaculation 89. Per‐act likelihood of transmission was estimated to be 0.11% (11 in 10,000) when the HIV‐negative person is the insertive partner 5.

▫ The possibility of HIV transmission through anal‐penile intercourse when the HIV‐positive partner has a low viral load, **or** uses a condom, **or** the HIV‐negative partner is taking PrEP varies from none to negligible depending on the context The likelihood is similar whether the receptive partner is male or female 85, 86.

There is negligible possibility of HIV transmission from an HIV‐positive partner who has a low viral load during anal‐penile intercourse. As discussed above, both the PARTNER study and the Opposites Attract study observed no transmission after approximately 39,000 acts of condomless anal sex when viral load was below 200 copies/mL 30, 92. In fact, there has not been a reported case of transmission from a person with an undetectable viral load in any clinical trial.

HIV cannot be transmitted when a condom is used correctly because HIV cannot pass through intact latex or polyurethane. Similarly, there is no possibility of HIV transmission when a person has an undetectable viral load.

### 2.4 The possibility of HIV transmission from casual contact, spitting and biting

#### 2.4.1 Casual contact

HIV cannot be transmitted via contact with an environmental surface such as a chair, bench or toilet; from food or drink; or from casual human contact such as hugging, sharing household objects or eating together.

HIV cannot survive long in air and is unable to penetrate intact skin. No case of HIV infection from contact with an environmental surface, food or drink or through casual human contact has ever been identified despite many scientific studies considering this possibility 93, 94, 95, 96, 97, 98.

#### 2.4.2 Biting and spitting

▫ There is no possibility of HIV transmission via contact with the saliva of an HIV‐positive person, including through kissing, biting or spiting.

Numerous studies have considered the possibility of HIV transmission via saliva but none has found any evidence, including a 1997 study of 34,000 cases in the UK 99. The absence of HIV transmission via saliva is attributed to two factors: saliva contains a very small amount of HIV 100, and several inhibitory components in oral secretions mean saliva acts to protect susceptible cells from HIV infection 101, 102, 103, 104, 105, 106.

▫ There is no possibility of HIV transmission from biting or spitting where the HIV‐positive person's saliva contains no, or a small quantity of, blood.

Current evidence suggests HIV cannot be transmitted even when saliva contains small quantities of blood. Despite early research suggesting a theoretical risk of transmission if saliva‐containing blood enters a person's body through contact with mucosal tissue (for example, landing in an eye or mouth), no cases of HIV transmission resulting from the spitting of blood have been reported 107. Consequently, it is our expert opinion that there is no possibility of HIV transmission from saliva containing small quantities of blood.

▫ The possibility of HIV transmission from biting where the HIV‐positive person's saliva contains a significant quantity of blood, **and** their blood comes into contact with a mucous membrane or open wound, **and** their viral load is not low or undetectable varies from none to negligible.

Many studies have detailed a large number of cases where bites have not resulted in HIV transmission 108, 109, 110, 111, 112 or found transmission to be unlikely 107, 109, 113, 114.

For transmission to be plausible in the case of biting, the HIV‐positive person must have blood in their mouth at the time of the bite, a sufficient amount of HIV must be present in the blood of the HIV‐positive person, and the bite must be deep enough to penetrate the HIV‐negative person's skin causing trauma and tissue damage 106, 107, 115. Even when all these conditions are present, the possibility of transmission during a single bite is negligible at most.

### 2.5 Significant improvements in life expectancy and quality of life for people living with HIV

The second section of this Consensus Statement considers the harms of HIV because persistent misconceptions exaggerating the harms of HIV infection appear to influence application of the criminal law 3. Criminal law takes into account the possible harms caused by a potential offence as well as the likelihood of the offence itself, thus, for example, definitions of bodily harm are distinct from grievous bodily harm, which are distinct from manslaughter or murder. Consequently, it is important to emphasize the huge changes in the outlook for people living with HIV that have been achieved over the past decades.

The natural course of untreated HIV infection varies widely from person to person 116. If untreated, most people experience an asymptomatic phase that lasts from two to 15 years, during which the virus replicates, gradually undermining their immune system. A small percentage of people with HIV have immune systems that block replication of the virus for an indefinite period 117, but the large majority of people eventually develop AIDS if untreated (approximately half within 10 years 118). AIDS is defined as the presence of specific laboratory markers and/or opportunistic infections and specific diseases which, if antiretroviral therapy is not commenced, eventually result in a person's death.

Antiretroviral therapies dramatically reduce HIV‐associated disease progression. Globally, treatment guidelines have been revised to recommend initiation of antiretroviral treatment immediately following diagnosis of HIV infection because most people on treatment will achieve an undetectable viral load and maintain a healthy immune system, will remain in good health, and will avoid the complications of long‐term HIV infection 119, 120. Even those who start treatment with a high viral load and adhere to therapy can expect a dramatic reduction in viral load, to a point where significant immune system recovery occurs so that they can enjoy good long‐term health 121. For many, effective treatment requires taking a single pill each day.

Studies from many countries have consistently shown that antiretroviral therapies have radically increased life expectancy, that life expectancy has continued to improve over time, and that the long‐term health and quality of life of people living with HIV has drastically improved 122, 123, 124, 125, 126, 127, 128, 129, 130, 131, 132, 133, 134, 135, 136, 137, 138, 139, 140, 141. Life expectancy for young people with HIV commencing antiretroviral therapy now approaches that of a young person in the general population 45, 132, 134, 135, 137. Furthermore, use of antiretroviral therapies has shifted cause of death of people living with HIV from traditional AIDS‐defining illnesses to non‐HIV‐related causes 142, 143 similar to those affecting the general population 144. Similarly, clinical management has shifted to include management and treatment of health issues associated with aging, including menopause and cardiovascular disease 143, 144, 145, 146, 147, 148, 149, 150, and interventions to influence “lifestyle choices” such as tobacco smoking 151. In some sub‐populations, ongoing clinical care has the potential to increase life expectancy of people living with HIV beyond that of their HIV‐negative counterparts 135.

Although HIV causes an infection that requires continuous treatment with antiretroviral therapy, people living with HIV can live long, productive lives including working, studying, travelling, having relationships, having and raising children, and contributing to society in various other ways.

### 2.6 Establishing proof of HIV transmission

The final section of this Consensus Statement recognizes the importance of the correct use of scientific and medical evidence in HIV‐related prosecutions where proof of actual transmission from one person to another is at issue.

International guidance on HIV in the context of the criminal law recommends that “proof of causation, in relation to HIV transmission, should always be based on evidence derived from a number of relevant sources, including medical records, rigorous scientific methods and sexual history” 1.

▫ Medical records can provide contextual information but cannot establish transmission between a complainant and a defendant.

The circumstances of the nature and timing of a sexual relationship or other potential sources of a person's HIV infection must be central to any case where sexual transmission of HIV is alleged. When available and lawfully obtained, medical records are valuable for identifying the last HIV‐negative and first HIV‐positive test of the complainant and the defendant. Considering the diagnostic window period of each test, this information can be used to establish the period during which the complainant acquired HIV and whether the defendant was HIV‐positive during this time. Importantly, whether the complainant or defendant was infected first cannot be based on who tested HIV‐positive first or which person brought charges against the other.

Information related to HIV viral load and CD4 counts included in medical records has sometimes been presented as evidence establishing the timing of HIV infection. However, viral loads and CD4 counts show considerable inter‐ and intra‐individual variation and therefore cannot be used to determine exactly when someone acquired HIV 152.

▫ Phylogenetic analysis can be used as a forensic tool. The results can be compatible with, but cannot conclusively prove, the claim that a defendant has infected a complainant. Importantly, phylogenetic results can exonerate a defendant when the results are not compatible with the allegation that the defendant infected the complainant.

Phylogenetic analysis compares the evolutionary relationship between different persons’ HIV, but results must be interpreted cautiously alongside other factual and medical evidence when used in criminal cases 153. The complexity of phylogenetic analysis arises, in part, from the fact that HIV is a fast‐evolving virus. Mutations of the virus occur repeatedly so that every person living with HIV has more than one virus variant 154. During transmission, a limited number of virus variants (one to a few) are transmitted, but these will also mutate to form new variants so that no two persons’ HIV is identical 155.

Phylogenetic analysis of HIV involves estimating the evolutionary relationships of HIV variants, for example, to investigate HIV transmission networks for public health purposes. In criminal cases, phylogenetic analysis involves investigating whether the complainant(s) and the defendant(s) are part of the same transmission network. The network is represented as a phylogenetic “tree.” Notably, the phylogenetic tree must be understood as an HIV gene tree, which may differ from the transmission history, because HIV variants may predate transmission or disappear after transmission 156 and because some persons in the transmission network may not have been diagnosed and/or sampled before constructing the tree.

HIV phylogenetics is very different from profiling of human DNA as, given the ongoing evolution of each person's HIV variants, phylogenetics cannot obtain an “exact match.” When there appears to be a “phylogenetic match” between two individuals’ HIV it means two or more variants are epidemiologically “linked”, not that they are the same 155, 157. HIV phylogenetic evidence *can* exonerate a defendant accused of transmitting HIV to a complainant because if the virus strains detected in the defendant and complainant are unrelated, the phylogenetic evidence conclusively contradicts the claim that the defendant was the source of the complainant's virus. 155, 158.

Recent advances in DNA sequencing and phylogenetics allow some consideration of direction and timing of transmission 159, 160, 161, 162, but these methods are currently neither precise nor accurate enough to prove who infected whom 155, 163. This is partly because there may always be unknown and undiagnosed individuals from the transmission network 155. Consequently, currently phylogenetic analysis cannot eliminate the possibilities that the complainant infected the defendant, that both were infected by a third party 158, 163, or more complex scenarios of transmission that have resulted in the defendant and complainant having HIV variants that are epidemiologically linked. The fact that having HIV does not protect against a subsequent “super”‐infection with a different variant adds complexity 158. In particular, confidence about the direction of infection is undermined when a defendant and complainant have engaged in numerous sexual acts which may have facilitated multiple transmission events back and forth 155.

Phylogenetic analysis is complex, and consequently it is important that HIV phylogenetics for forensic purposes is performed and interpreted by experts who fully understand the limitations of the technique and explicitly state these limitations in written reports and oral testimony. Interpretation of phylogenetic results for forensic purposes requires expertise about phylogenetics and the distinction between virus evolutionary trees and transmission histories. This is not straightforward and methodologies have not yet been standardized 155. The reliability of evidence derived from phylogenetic analysis depends on a number of methodological factors including use of adequate “local controls” 164, 165, 166 and database sequences 167, 168, 169 which must be selected using consistent selection criteria 155. International research shows that phylogenetic evidence used in criminal trials has not always satisfied these requirements 155.

## 3 Conclusions

Given the evidence presented in this document, we strongly recommend that more caution be exercised when considering criminal prosecution, including careful appraisal of current scientific evidence on HIV‐related risks and harms. This is instrumental to reduce stigma and discrimination and to avoid miscarriages of justice.

In this context, we hope this Consensus Statement will encourage governments and those working in the legal and judicial system to pay close attention to the significant advances in HIV science that have occurred over the last three decades, and make all efforts to ensure that a correct and complete understanding of current scientific knowledge informs any application of the criminal law in cases related to HIV.

## Competing interests

The authors have no competing interests to declare.

## Authors’ contributions

All authors participated in numerous rounds of discussions, writing and editing of this Consensus Statement.

## Supporting information


**Supplementary Material S1.** Endorsers of the Expert Consensus Statement.

**Supplementary Material S2.** Executive Summary Expert Consensus Statement.

**Supplementary Material S3.** Expert Consensus Statement FRENCH Translation.

**Supplementary Material S4.** Expert Consensus Statement RUSSIAN Translation.

**Supplementary Material S5.** Expert Consensus Statement SPANISH Translation.
