# Supplementary material for: Expert consensus statement on the science of HIV in the context of criminal law
Source: J Int AIDS Soc. 2018 Jul 25;21(7):e25161. doi: 10.1002/jia2.25161 (PMC6058263; doi:10.1002/jia2.25161)
Supplement: Supplementary file 1 — Supplementary Material S1. Endorsers of the Expert Consensus Statement. [file JIA2-21-e25161-s001.docx]

**Endorsers of The Expert Consensus Statement on the Science of HIV in the Context of Criminal Law**

The following expert HIV scientists are originating endorsers of the Expert Consensus Statement on the Science of HIV in the Context of Criminal Law.

| **Name** | **Affiliation** | **Country** |
| --- | --- | --- |
| Michel Alary | Laval University | Canada |
| Pascale Allotey | United Nations University International Institute for Global Health | Malaysia |
| Francisco Antunes | Universidade de Lisboa | Portugal |
| Vic Arendt | Centre Hospitalier de Luxembourg | Luxembourg |
| Jose Arribaz | Hospital Universitario La Paz | Spain |
| Raja Iskandar Shah Raja Azwa | University of Malaya | Malaysia |
| Henrique Barros | University of Porto | Portugal |
| Geoffrey Barrow | University of the West Indies | Jamaica |
| Josip Begovac | University of Zagreb | Croatia |
| Azizbek Boltaev | Human Research and Development Center | Uzbekistan |
| Carlos Brites | Federal University of Bahia | Brazil |
| Carlos F. Cáceres | Cayetano Heredia University | Peru |
| Mohamed Chakroun | University of Monastir | Tunisia |
| Roy Chan | National University of Singapore | Singapore |
| Dan Clutterbuck | University of Edinburgh | Scotland |
| Myron S. Cohen | University of North Carolina at Chapel Hill | USA |
| James Curran | Emory University | USA |
| François Dabis | ANRS: France Recherche Nord & sud Sida-hiv hépatites | France |
| Rémy Demeeste | BREACH Foundation | Belgium |
| Bavon Mukanya Dikuyi | Caritas Congo ASBL | D. R. of Congo |
| Daniel Elbirt | Kaplan Medical Center | Israel |
| Ana Espada de Sousa | University of Lisbon | Portugal |
| Eric Florence | Institute of Tropical Medicine Antwerp | Belgium |
| Jean-Christophe Goffard | Erasmus Hospital | Belgium |
| Deniz Gokengin | Ege University | Turkey |
| Angel Guerra-Marquez | Mexican Institute for Social Security | Mexico |
| Jean-Christophe Goffard | ULB Hôpital Erasme | Belgium |
| Fritz van Griensven | University of California, San Francisco | USA |
| Catherine A. Hankins | Amsterdam Institute for Global Health and Development | The Netherlands |
| Hakima Himmich | Medical School of Casablanca | Morocco |
| Bernard Hirschel | Geneva University Hospitals | Switzerland |
| John Idoko | University of Jos | Nigeria |
| Aikichi Iwamoto | University of Tokyo | Japan |
| Andreas Jahn | University of Washington | USA |
| Quarraisha Abdool Karim | Centre for the AIDS Program of Research in South Africa | South Africa |
| Mehdi Karkouri | University of Hassan II Casablanca | Morocco |
| Rupert Kaul | University of Toronto | Canada |
| Michel Kazatchkine | The Graduate Institute, Geneva | Switzerland |
| Tetiana Kiriazova | Ukrainian Institute of Public Health Policy | Ukraine |
| Marina Klein | McGill University | Canada |
| Itzchak Levy | Tel Aviv University | Israel |
| Sharon Lewin | The University of Melbourne | Australia |
| Jens Lundgren | University of Copenhagen | Denmark |
| Shuzo Matsushita | Kumamoto University | Japan |
| Kenneth Mayer | Harvard University | USA |
| Rak Nandwani | NHS Greater Glasgow and Clyde | Scotland |
| Ibrahim N’Doye | CRCF/ANRS CHU Hôspital fann Dakar | Senegal |
| Jean William Pape | Weill Cornell Medical College – Les Centres GHESKIO | Haiti |
| Nittaya Phanuphak | The Thai Red Cross AIDS Research Center | Thailand |
| Praphan Phanuphak | The Thai Red Cross AIDS Research Center | Thailand |
| Peter Piot | London School of Hygiene & Tropical Medicine | United Kingdom |
| Mario Poljak | University of Ljubljana | Slovenia |
| Anton Pozniak | International AIDS Society | United Kingdom |
| Flor Pujol | Venezuelan Institute for Scientific Research | Venezuela |
| Peter Reiss | University of Amsterdam | Netherlands |
| Armin Rieger | Medical University of Vienna | Austria |
| Matti Ristola | Helsinki University Hospital | Finland |
| Alison Rodger | University College London | United Kingdom |
| Horacio Salomon | University of Buenos Aires | Argentina |
| Peter Saxton | University of Auckland | New Zealand |
| Mauro Schechter | Federal University of Rio de Janeiro | Brazil |
| Izukanji Sikazwe | Centre for Infectious Disease Research in Zambia | Zambia |
| Bruno Spire | Aix-Marseille University | France |
| De Wit Stephane | Université libre de Bruxelles | Belgium |
| János Szlávik | Szent László Hospital | Hungary |
| Michael L. Tan | University of the Philippines Diliman | Philippines |
| Nuno Taveira | Instituto Universitário Egas Moniz | Portugal |
| Mark Tyndall | University of British Colombia | Canada |
| Francois Venter | Wits Reproductive Health and HIV Institute | South Africa |
| Zunyou Wu | National Center for AIDS/STD Control and Prevention | China |
| Robert Zangerle | Medical University of Innsbruck | Austria |

1 July 2018
