# Supplementary material for: Expert consensus statement on the science of HIV in the context of criminal law
Source: J Int AIDS Soc. 2018 Jul 25;21(7):e25161. doi: 10.1002/jia2.25161 (PMC6058263; doi:10.1002/jia2.25161)
Supplement: Supplementary file 2 — Supplementary Material S2. Executive Summary Expert Consensus Statement. [file JIA2-21-e25161-s002.pdf]

Executive Summary: Expert Consensus Statement on the Science of HIV in the Context of Criminal Law

Why was this consensus statement needed?

- At least 68 countries have laws that specifically criminalise HIV non-disclosure, exposure, or transmission and thirty-three countries are known to have applied other criminal laws in similar cases.
- People living with HIV continue to be accused, arrested, prosecuted and/or convicted for non-disclosure, (possible or perceived) exposure or transmission of HIV in cases where no harm was intended; where HIV transmission did not occur, was extremely unlikely or was not possible; and where transmission was neither alleged nor proven.
- Limited understanding of current HIV science reinforces stigma and can lead to miscarriages of justice while undermining efforts to address the HIV epidemic.

What are the take-home messages of the statement?

- The possibility of HIV transmission during a single act of vaginal or anal sex ranges from none to low.
- The possibility of HIV transmission during a single act of oral sex ranges from none to negligible.
- There is no possibility of HIV transmission during a single act of vaginal, anal or oral sex where a condom has been used correctly (i.e. it was worn throughout sex and its integrity was not compromised).
- There is no possibility of HIV transmission during a single act of vaginal, anal or oral sex when the HIV-positive partner has an undetectable viral load.
- The possibility of HIV transmission during a single act of vaginal or anal sex when the HIV-positive partner has a low viral load ranges from none to negligible.
- There is no possibility of HIV transmission through saliva even when it contains small quantities of blood.
- The possibility of HIV transmission from biting ranges from none to negligible.
- Modern antiretroviral therapies have improved the life expectancy of most people living with HIV who have access to care to a point similar to their HIV-negative counterparts, transforming HIV infection into a chronic manageable health condition.
- Phylogenetic analysis can be compatible with, but cannot conclusively prove, the claim that a defendant has infected a complainant. Importantly, phylogenetic results can exonerate a defendant when the results are not compatible with the allegation that the defendant infected the complainant.

What are the implications for policy, practice, and research of the evidence in the statement?

- Better understanding of recent scientific advances should help courts and their expert witnesses (clinicians, scientists, advocates) to provide clearer statements in situations where charges have been brought against an individual where HIV exposure or transmission is alleged.
