# Supplementary material for: Expert consensus statement on the science of HIV in the context of criminal law
Source: J Int AIDS Soc. 2018 Jul 25;21(7):e25161. doi: 10.1002/jia2.25161 (PMC6058263; doi:10.1002/jia2.25161)
Supplement: Supplementary file 3 — Supplementary Material S3. Expert Consensus Statement FRENCH Translation. [file JIA2-21-e25161-s003.pdf]

Déclaration de consensus d'experts sur la connaissance scientifique relative au VIH dans le contexte du droit pénal

Françoise Barré-Sinoussi[1], Salim S. Abdool Karim[2,3, 4], Jan Albert[5], Linda-Gail Bekker[6], Chris Beyrer[7], Pedro Cahn[8, 9, 10], Alexandra Calmy[11], Beatriz Grinsztejn[12], Andrew Grulich[13], Adeeba Kamarulzaman[14], Nagalingeswaran Kumarasamy[15], Mona Rafik Loutfy[16, 17, 18], Kamal Marhoum El Filali[19], Souleymane Mboup[20], Julio S.G Montaner[21,22], Paula Munderi[23], Vadim Pokrovsky[24,25], Anne-Mieke Vandamme[26, 27], Benjamin Young[28], Peter Godfrey-Faussett[§29,30]

1. Institut Pasteur, Paris, France
2. École de santé publique Mailman, Université Columbia, New York, États-Unis
3. Centre pour le programme de recherche sur le SIDA en Afrique du Sud, Université de KwaZulu-Natal, Durban, Afrique du Sud
4. Weill Medical College, Université Cornell, New York, États-Unis
5. Département de microbiologie, biologie tumorale et cellulaire, Institut Karolinska, Stockholm, Suède
6. Institut des maladies infectieuses et de médecine moléculaire, Université du Cap, Cape Town, Afrique du Sud
7. Département d'épidémiologie, Centre de recherche sur le sida et Centre de santé publique et des droits de l'homme, École de santé publique John Hopkins Bloomberg, Maryland, États-Unis
8. Unité des maladies infectieuses, Hôpital Juan A. Fernandez Buenos Aires, Argentine
9. École de médecine de l'Université de Buenos Aires, Buenos Aires, Argentine
10. Fundación Huésped, Buenos Aires, Argentine
11. Division des maladies infectieuses de l'Hôpital universitaire de Genève, Genève, Suisse
12. Institut national d'infectiologie Evandro Chagas-Fiocruz, Fiocruz, Rio de Janeiro, Brésil
13. Kirby Institute, Université de Nouvelle-Galles du Sud, Sydney, Australie
14. Faculté de médecine, Université de Malaisie, Kuala Lumpur, Malaisie
15. Centre médical YRGCARE, Services de santé volontaires, Chennai, Inde
16. Women's College Research Institute, Toronto, Canada
17. Women's College Hospital, Toronto, Canada
18. Département de médecine, Université de Toronto, Canada
19. Unité des maladies infectieuses, Hôpital Universitaire Ibn Rochd, Casablanca, Maroc
20. Institut de recherche en santé, de surveillance épidémiologique et de formation, Dakar, Sénégal
21. Faculté de médecine, Université de la Colombie-Britannique, Vancouver, Canada
22. Centre d'excellence de la Colombie-Britannique sur le VIH/sida, Vancouver, Canada
23. Association internationale des fournisseurs de soins contre le sida, Kampala, Ouganda
24. Université de l'amitié des peuples russes (RUDN-University), Moscou, Fédération de Russie
25. Institut central de recherche en épidémiologie, Service fédéral de protection des droits des consommateurs et de surveillance du bien-être humain, Moscou, Fédération de Russie
26. KU Leuven, Département de microbiologie et d'immunologie, Institut Rega de recherche médicale, Virologie clinique et épidémiologique, Louvain, Belgique
27. Centre de santé mondiale et de médecine tropicale, Unité de microbiologie, Institut d'hygiène et de médecine tropicale, Université de Lisbonne, Lisbonne, Portugal
28. Association internationale des fournisseurs de soins du sida, Washington, DC, États-Unis
29. ONUSIDA, Genève, Suisse

30. Département des maladies infectieuses et tropicales, London School of Hygiene and Tropical Medicine, Londres, Angleterre

[§] **Auteur principal** : Professeur Peter Godfrey-Faussett

Adresse : ONUSIDA, Avenue Appia 20, 1211 Genève, Suisse

Téléphone : + 41 22 791 4054

Courriel : godfreyp@unaids.org

Courriels des auteurs :

FB-S : francoise.barre-sinoussi@pasteur.fr

JA : Jan.Albert@ki.se

SSAK : Salim.AbdoolKarim@caprisa.org

L-GB : linda-gail.bekker@hiv-research.org.za

CB : cbeyrer@jhu.edu

PC : pedro.cahn@huesped.org.ar

AC : alexandra.calmy@hcuge.ch

PG-F : faussettp@unaids.org

BG : beatriz.grinsztejn@gmail.com

AG : agrulich@kirby.unsw.edu.au

AK : adeeba@ummc.edu.my

NM : kumarasamy@yrgcare.org

MRL : mona.loutfy@wchospital.ca

KMEF : mefkamal@hotmail.com

SM : souleymane.mboup@iressef.org

JSGM : jmontaner@cfenet.ubc.ca

PM : munderip@gmail.com

VP : pokrovsky.vad@yandex.ru

A-MV : annemie.vandamme@kuleuven.be

BY : byoung@iapac.org

Mots clés :

- droits de l'homme
- droit et politique
- facteurs de risque
- politique
- criminalisation
- droit pénal
- poursuites judiciaires

Résumé

Introduction

À l'échelle mondiale, les poursuites pour non-divulgation, exposition ou transmission du VIH sont souvent liées à des rapports sexuels, des cas de morsures ou de crachements. Certaines personnes ont été poursuivies alors qu’elles n’avaient aucune intention de causer un préjudice, qu’elles n’ont pas transmis le VIH et que la transmission était en soi extrêmement improbable ou impossible. Cela suggère que les poursuites pénales ne sont pas toujours guidées par les meilleures données scientifiques et médicales probantes disponibles.

Discussion

Vingt scientifiques de différentes régions du monde ont élaboré cette déclaration de consensus d'experts pour répondre aux problématiques liées à l’utilisation des données scientifiques relatives au VIH par le système de justice pénale. Les meilleures données scientifiques et médicales disponibles en matière de transmission du VIH, d'efficacité des traitements et de preuves phylogénétiques médico-légales ont fait l’objet d’une analyse détaillée et sont présentées ici de sorte à être mieux comprises dans un contexte pénal.

Cette déclaration de consensus se limite à la possibilité de transmission du VIH associée aux actes les plus souvent en cause dans les affaires pénales. La possibilité de transmission du VIH associée à un acte spécifique est décrite sur la base d’un continuum de risque sachant que cette possibilité dépend d'une série de facteurs croisés comme la charge virale, l'utilisation d’un préservatif et le recours à d’autres pratiques de réduction des risques. Les données probantes actuelles suggèrent que la possibilité de transmission du VIH au cours d'un seul acte sexuel, de morsure ou de crachement, varie entre aucune possibilité et une faible possibilité.

D'autres recherches ont examiné l'effet positif des thérapies antirétrovirales modernes sur la santé. Les traitements ont permis d’améliorer l'espérance de vie de la plupart des personnes vivant avec le VIH au point qu’elle est désormais similaire à celle des personnes séronégatives, transformant ainsi l'infection par le VIH en maladie chronique gérable. Enfin, l'examen de l'utilisation de preuves scientifiques devant les tribunaux a révélé que l'analyse phylogénétique ne peut, à elle seule, prouver au-delà d’un doute raisonnable qu'une personne en a infecté une autre. En revanche, elle peut être utilisée pour disculper l’accusé.

Conclusions

L'utilisation des données scientifiques probantes les plus récentes dans le cadre des affaires pénales peut limiter les poursuites et les condamnations injustes. Les auteurs recommandent la prudence lorsqu’une poursuite pénale est envisagée et encouragent les gouvernements et les acteurs de la justice à prêter une attention particulière aux progrès importants réalisés dans le domaine du VIH au cours des trente dernières années afin que l’application de la loi, dans des cas liés au VIH, soit effectivement fondée sur les données scientifiques probantes actuelles.

Introduction

Au moins 68 pays ont des lois qui criminalisent spécifiquement la non-divulgation, l'exposition ou la transmission du VIH. Trente-trois pays ont déjà appliqué d'autres dispositions de droit pénal dans des cas similaires (données non publiées, HIV Justice Network, 2018). La plupart des poursuites sont liées au risque perçu de transmission du VIH associé aux rapports sexuels, mais des poursuites ont également été engagées pour des cas de morsure ou de crachement (données non publiées, HIV Justice Network, 2018). Ces lois et poursuites n'ont pas toujours été guidées par les meilleures données scientifiques et médicales disponibles [1]. Elles n'ont pas évolué pour refléter les nouvelles connaissances sur le VIH et son traitement, et elles peuvent être influencées par la stigmatisation et les peurs associées au VIH qui persistent dans nos sociétés [2]. Le VIH continue de faire l'objet d'un traitement particulier, avec des poursuites criminelles dans des cas où il n'y avait aucune intention de causer un préjudice; où il n'y a eu aucune transmission; où la transmission était impossible ou était extrêmement improbable ; et dans des cas où la transmission n'était ni alléguée ni prouvée [1,3].

Dans ce contexte, vingt scientifiques, de différentes régions du monde, ayant une expertise reconnue dans le domaine de la recherche scientifique, de l'épidémiologie et des soins aux patients ont élaboré cette déclaration de consensus — inquiets que le droit pénal soit parfois appliqué de manière incompatible avec les données probantes médicales et scientifiques contemporaines, et notamment d'une manière exagérant à la fois le risque de transmission du VIH et l'impact du VIH sur la santé et le bien-être. Cette compréhension limitée des données scientifiques actuelles sur le VIH renforce la stigmatisation et peut conduire à des erreurs judiciaires. Elle peut également compromettre les efforts déployés pour lutter contre l'épidémie de VIH [4]. En plus des vingt auteurs, la déclaration de consensus a été approuvée par d'autres scientifiques du monde entier (voir document supplémentaire S1) et par la International AIDS Society, the International Association of Providers of AIDS Care et le Programme commun des Nations Unies sur le VIH / sida (un résumé de la déclaration est disponible, voir document supplémentaire S2).

Cette déclaration de consensus vise à aider les experts scientifiques appelés à témoigner dans des affaires pénales de non-divulgation, d'exposition (apparente ou possible) ou de transmission alléguée du VIH. Elle offre une opinion d'experts sur les dynamiques de transmission du VIH au niveau individuel (c.-à-d. la « possibilité » de transmission), les conséquences à long terme d'une infection chronique au VIH (c.-à-d. le « préjudice »), et l'utilisation de l'analyse phylogénétique comme preuve de la transmission. La déclaration de consensus décrit la possibilité de transmission du VIH associée à un acte spécifique entre

individus à un moment donné et dans des circonstances précises – puisque c'est ce qui est généralement en cause dans des affaires pénales — et cherche à communiquer les données scientifiques actuelles relatives au VIH d'une manière qui soit compréhensible pour une audience non scientifique. La déclaration de consensus a été traduite, à partir de l'anglais, en français, en russe et en espagnol (voir les documents supplémentaires S3, S4, S5).

Discussion

La première partie de cette déclaration porte sur la possibilité de transmission du VIH associée à des actes spécifiques généralement en cause dans les poursuites criminelles : rapports sexuels, morsure ou crachement [3]. Elle ne porte pas sur d'autres modes éventuels de transmission du VIH comme la transfusion sanguine, une piqûre d'aiguille, l'injection de drogues ou l'allaitement.

Lors d'une première réunion à Seattle (février 2017) les auteurs se sont mis d'accord sur le contenu et le cadre de la déclaration de consensus. Une analyse détaillée de la littérature a été réalisée à partir d'une recherche d'études publiées en anglais jusqu'en avril 2017 à l'aide de la base de données en ligne PubMed. Des termes de recherche spécifiques relatifs à la possibilité de transmission du VIH ont été utilisés, notamment « *HIV and viral load* » (VIH et charge virale), « *HIV sexual transmission risk per act* » (risque de transmission sexuelle du VIH par acte), « *oral sex HIV transmission* » (sexe oral transmission du VIH), « *anal sex HIV transmission* » (sexe anal transmission du VIH, « *vaginal sex HIV transmission condom per act* » (sexe vaginal transmission du VIH par acte préservatif), « *anal sex HIV transmission condom per act* » (sexe anal transmission du VIH par acte préservatif), et « *anal sex HIV transmission circumcision per act* » (sexe anal transmission du VIH par acte circoncision). Des articles clés ont été utilisés pour rechercher d'autres articles connexes. La préférence a été accordée aux méta-analyses, aux revues et aux études importantes. D'autres sources ont été identifiées par les auteurs experts. Des résumés de conférences scientifiques ont été utilisés au besoin.

Les auteurs ont ensuite participé à plusieurs cycles de rédaction et de révision du texte, en tenant compte des meilleures données de la recherche scientifique et médicale disponibles sur la base de la hiérarchie suivante : revue systématique des essais cliniques randomisés ; essais cliniques randomisés ; et études comparatives (c.-à-d., études de cohorte, études cas-témoins et études de contrôle historiques). Deux téléconférences ont été organisées pour discuter d'un avant-projet de déclaration de consensus, suivi de trois cycles de révision du texte par

correspondance électronique par tous les auteurs. Des experts juridiques nationaux et internationaux, y compris des membres du personnel de l'ONUSIDA, ont été consultés sur l'application du droit pénal dans des affaires liées au VIH. Une deuxième réunion en personne a été organisée à Paris (juillet 2017) pour discuter de certaines questions relatives à l'analyse des données. Cette réunion a été suivie d'une nouvelle série de commentaires et de modifications par les auteurs visant à s'assurer que tous étaient d'accord que la déclaration de consensus transmettait fidèlement les données scientifiques actuelles sur la transmission du VIH, le préjudice et l'utilisation de preuves scientifiques devant les tribunaux.

Les auteurs ont examiné les résultats numériques et les estimations statistiques de toutes les études citées ici, y compris les résumés de données provenant de rapports présentés sous forme systématique ou sous forme de tableau (par exemple, les travaux de Patel et al. [5]). Les données probantes établissant une estimation de la possibilité de transmission du VIH associée à différents actes varient en termes de type (de données) et de qualité. Les auteurs en ont tenu compte dans leur évaluation de la possibilité de transmission associée à différents actes. Les auteurs ont réparti les données probantes concernant la transmission du VIH associées à différents actes en trois catégories (Tableau 1).

Tableau 1 : Échelle de qualité des données probantes concernant la possibilité de transmission du VIH

Les actes pour lesquels la possibilité de transmission peut être estimée avec un certain degré de certitude parce que plusieurs études de cohorte ont été réalisées (par exemple, des actes tels que le sexe vaginal ou anal).
Les actes pour lesquels la possibilité de transmission peut être estimée avec moins de certitude à partir de rapports de cas isolés, de plausibilité biologique ou de modèles mathématiques (par exemple, des actes tels que le sexe oral ou la transmission via un liquide pré-éjaculatoire).
Les actes pour lesquels la transmission est biologiquement improbable car les conditions requises pour la transmission ne sont pas présentes (par exemple, le crachement).

Pour présenter les données probantes, les auteurs ont cherché à utiliser des concepts scientifiques d'une manière qui soit utile dans le contexte du droit pénal. Par exemple, le concept statistique d'intervalles de confiance est conçu pour traiter l'incertitude inhérente aux résultats découlant de l'échantillonnage d'un sous-ensemble d'une population. Les intervalles de confiance revêtent une importance particulière en cas de probabilités équivalentes ou proches de zéro, car le fait que quelque chose n'ait pas été observé au cours d'une étude ne suffit pas à prouver qu'il ne pourrait jamais se réaliser. Plus l'étude est de grande envergure, plus les auteurs pourront estimer avec précision que la probabilité est effectivement de zéro. Une probabilité nulle calculée à partir de données d'études est donc toujours associée à un

intervalle de confiance allant de zéro à une petite probabilité. Il est important que le calcul des intervalles de confiance ne soit pas mal interprété, ce qui pourrait exagérer des possibilités de transmission en réalité peu probables et d'ordre théorique.

Trois catégories de possibilités de transmission, situées le long d'un continuum, ont été développées sur la base de ces considérations méthodologiques et des études citées dans la déclaration de consensus, afin de présenter pour chaque acte spécifique, la possibilité de transmission du VIH y afférente (tableau 2).

Tableau 2 : Définir la possibilité de transmission du VIH au cours d'un acte unique et spécifique

**Terminologie pour cette déclaration**	**Possibilité de transmission par acte**
Faible possibilité	La transmission pendant un seul acte est possible mais la probabilité est faible.
Possibilité négligeable	La transmission lors d'un acte unique est extrêmement improbable, rare ou sa probabilité est minime.
Aucune possibilité	La possibilité de transmission au cours d'un même acte est soit biologiquement invraisemblable soit effectivement nulle.

Il est important de noter que cette déclaration de consensus ne se veut pas un document de santé publique visant à informer les messages et les programmes de prévention du VIH, de traitement ou de soins. Son approche, fondée sur le risque au niveau individuel, est pertinente dans le cadre de la justice pénale. Elle se distingue de la description du risque au niveau de la population utilisée dans le domaine de la santé publique et qui décrit généralement le risque associé aux rapports sexuels comme allant du « risque faible » au « risque élevé ». Les différences entre les catégories utilisées dans le domaine de la santé publique et celles utilisées dans cette déclaration de consensus reflètent à la fois des considérations d'ordre historique et de contexte. Premièrement, les catégories de santé publique utilisées pour décrire les risques de transmission du VIH ont été élaborées au début de l'épidémie de VIH, avant l'émergence des données récentes sur la transmission du VIH. Deuxièmement, ces catégories décrivent le risque relatif (et non le risque absolu) de transmission du VIH dans le but d'aider les gens à prendre des mesures pour réduire la possibilité de transmission du VIH en comparant différents actes.

Bien que le recours à cette terminologie simplifiée de santé publique visait, au départ, à faciliter la promotion de campagnes efficaces de prévention, les catégories généralisées qui en résultent, compliquent aujourd'hui la formulation de messages de santé publique fondés sur les données les plus récentes [6] - y compris les données relatives aux différents facteurs

de risques associés à certains actes spécifiques comme la charge virale par exemple. Dans certains cas, la caractérisation du risque associé à certains actes sexuels, telle que communiquée par la terminologie utilisée par la santé publique, a été mal employée dans le contexte pénal, comme illustré par le cas canadien de Mabior [7,8]. C'est pourquoi, bien que les rapports sexuels soient un mode de transmission du VIH courant au niveau de la population mondiale, cette déclaration de consensus reconnaît que la possibilité de transmission du VIH associée à un seul rapport sexuel varie entre aucune possibilité et une possibilité faible, tandis que la possibilité de transmission associée au crachement ou à la morsure varie entre aucune possibilité et une possibilité négligeable. L'approche adoptée dans cette déclaration de consensus, pertinente dans le contexte pénal, est similaire à celle utilisée dans d'autres déclarations de consensus scientifiques au niveau national en Australie [9], au Canada [10], en Suède [11] et en Suisse [12].

Possibilité de transmission : vue d'ensemble

Le VIH ne se transmet pas facilement d'une personne à l'autre. C'est un virus relativement fragile qui se transmet par des voies bien spécifiques. Il ne se transmet pas par l'air, par gouttelettes, par fomite, par contact ou par transmission vectorielle et ne peut pas pénétrer la peau humaine intacte [13].

Pour qu'il y ait transmission, certaines conditions de base doivent être réunies :

- Il doit y avoir une quantité suffisante de virus dans des fluides corporels bien particuliers (qui sont le sang, le sperme, le liquide pré-éjaculatoire, les sécrétions rectales et vaginales et le lait maternel).
- Une quantité suffisante d'au moins un de ces fluides corporels doit entrer en contact direct avec certaines parties du corps d'une personne séronégative constituant une porte d'entrée où l'infection peut être initiée. Ce sont généralement les membranes muqueuses, les tissus endommagés ou les ulcères enflammés ; mais pas la peau intacte.
- Le virus doit ensuite vaincre les défenses immunitaires afin que l'infection puisse s'installer et se propager.

La plupart des activités du quotidien ne comportent aucun risque de transmission du VIH parce que les conditions précitées ne sont pas réunies. Laissant de côté la question de la transmission verticale du VIH, un contact intime, tel qu'un rapport sexuel, est généralement nécessaire pour qu'il y ait transmission. Mais même dans ce cas, la probabilité de

transmission, par acte, varie de zéro à faible (avec des estimations allant de 0 à 1,4 % par acte) [5].

Facteurs influençant la possibilité de transmission du VIH

La possibilité de transmission du VIH au niveau individuel varie en fonction d'une gamme de facteurs croisés. Lorsque plusieurs de ces facteurs se croisent, leur effet peut être soit minimisé ou amplifié à différents degrés [14].

- ### L'utilisation correcte d'un préservatif empêche la transmission du VIH

Utilisé correctement, un préservatif (masculin ou féminin) empêche la transmission du VIH car la porosité du préservatif protège contre les plus petits agents pathogènes sexuellement transmissibles, y compris le VIH [15]. Les préservatifs en latex et en polyuréthane agissent comme une barrière physique imperméable à travers laquelle le VIH ne peut passer. Une utilisation correcte du préservatif implique que le préservatif ne soit pas endommagé et qu'il soit porté pendant toute la durée de l'acte sexuel en question. La transmission du VIH n’est pas possible lorsqu’un préservatif est utilisé correctement.

Des études au niveau de la population ont montré que l'utilisation constante du préservatif lors de rapports sexuels anaux ou vaginaux réduit considérablement la possibilité de transmission du VIH et ce, même en tenant compte de cas d'utilisation incorrecte ou de rupture du préservatif [16-21]. Par exemple, une méta-analyse de 14 études a montré que l'utilisation constante, sur une longue période, de préservatifs masculins lors de rapports sexuels vaginaux, réduit la possibilité de transmission du VIH d'au moins 80 % [22]. Cependant, des recherches plus récentes suggèrent que ce pourcentage pourrait être sous-estimé [23]. En effet, la méta-analyse mentionnée ci-dessus inclut des méthodes d'analyse de données qui ne sont pas standards et qui pourraient avoir conduit à un effet de recrutement et d'autres biais, susceptibles d'abaisser le niveau de prévention observé. [22,23].

La recherche au niveau de la population n'est pertinente qu’en cas d’actes sexuels multiples et si on ne peut savoir si un préservatif a été correctement utilisé pour chacun de ces actes. Par ailleurs, l'estimation au niveau de la population de l'efficacité du préservatif de l’ordre de 80 % n’est pas, en soit, une estimation du risque de transmission du VIH mais doit être appliquée au risque associé à différents actes sexuels. Par exemple, si le risque estimé de transmission du VIH d'un homme séropositif à une femme lors d'un seul rapport sexuel vaginal sans préservatif est de 0,08 % [5], alors le risque de transmission avec préservatif

peut être compris comme *au moins* 80 % plus bas, soit de 0,016 % (moins de 2 sur 10 000) [5]. Il est important de noter qu'en présence d'autres facteurs réduisant les risques de transmission (une charge virale faible ou un retrait avant l'éjaculation), la possibilité de transmission du VIH, même en cas d'utilisation incorrecte du préservatif, est encore réduite.

Encore une fois, le VIH ne peut pas se transmettre lorsqu'un préservatif est utilisé correctement (c'est-à-dire, qu'il a été porté tout au long de l'acte sexuel en question et qu'il n'a pas été endommagé). Les estimations au niveau de la population s'appliquent uniquement aux situations comprenant plusieurs cas d'utilisation du préservatif y compris des cas occasionnels d'utilisation incorrecte ou de rupture.

- Une charge virale faible ou « indétectable » réduit considérablement ou élimine la possibilité de transmission du VIH

  Peu de temps après une infection par le VIH, la charge virale est très élevée, mais elle diminue généralement au cours des premières semaines, à mesure que le système immunitaire réagit. Si une personne ne commence pas de traitement, sa charge virale restera stable pendant un certain temps, tandis que son système immunitaire s'affaiblira progressivement. Au stade avancé de l'infection au VIH, la charge virale augmente généralement de nouveau.

  La thérapie antirétrovirale empêche le VIH de se répliquer, réduisant ainsi considérablement la charge virale présente dans les fluides corporels. Lorsqu'une personne commence un traitement antirétroviral efficace, sa charge virale chute habituellement en quelques semaines ou mois à un niveau qui ne peut être détecté par les tests sanguins de laboratoire couramment utilisés à l'heure actuelle (« indétectable »). La disponibilité des tests de charge virale et les seuils de détection varient selon les régions du monde, avec des seuils de détection allant d'environ 20 copies virales/ml à 400 copies / ml. Un faible pourcentage de personnes vivant avec le VIH (souvent appelées asymptomatiques à long terme) ont une charge virale faible sans traitement antirétroviral parce que leur système immunitaire est capable de contrôler le virus [24-28].

  La réduction de la charge virale améliore la fonction immunitaire et diminue considérablement la probabilité à long terme de maladie et de décès. Elle réduit

également considérablement la possibilité de transmission du VIH [29-31]. La réduction de la charge virale est associée à une diminution concomitante de la probabilité de transmission du VIH [32-35], ce qui signifie que beaucoup de personnes sous traitement ne peuvent pas transmettre le VIH.

De récentes études clés (à savoir, HPTN052, PARTNER et Opposites Attract) impliquant des couples sérodiscordants hétérosexuels et masculins n'ont identifié aucun cas de transmission sexuelle lorsque le partenaire séropositif avait une charge virale indétectable [29,30,36,37]. Ces résultats ont transformé les messages de santé publique. Par exemple, les Center for Disease Control and Prevention aux États-Unis décrivent désormais la possibilité de transmission du VIH lorsqu'une personne séropositive a une charge virale indétectable (à la suite d'un traitement antirétroviral efficace) comme comportant « dans les faits », « aucun risque » [6].

En 2011, l'étude HPTN052 (menée au Botswana, au Brésil, en Inde, au Kenya, au Malawi, en Afrique du Sud, en Thaïlande, aux États-Unis et au Zimbabwe), évaluant l'impact d'une initiation précoce de traitement, n'a révélé aucune transmission à partir de 1763 personnes séropositives sous traitement antirétroviral ayant une charge virale stable inférieure à 400 copies/ml. Les partenaires des participants séropositifs au VIH ont été suivis pour une durée correspondant à 8509 années-personnes. Il n'a y eu transmission à partir de personnes sous traitement que lorsque celles-ci étaient en début de traitement (avant que la charge virale ne soit stabilisée à moins de 400 copies) ou que leur charge virale était supérieure à 1000 copies/ml pendant deux visites consécutives [29,37].

Les études PARTNER et Opposites Attract n'ont identifié aucune transmission du VIH à partir de personnes ayant une charge virale inférieure à 200 copies/ml après plus de 75 000 rapports sexuels vaginaux ou anaux sans préservatif [18,30,38]. Dans l'étude PARTNER, les couples hétérosexuels ont fait état d'environ 36 000 actes sexuels sans préservatif et les couples masculins homosexuels d'environ 22 000 [30]. Aucune transmission du VIH n'a été observée entre les partenaires ayant participé à l'étude. Il y a eu onze nouvelles infections au VIH. Cependant, l'analyse phylogénétique a révélé qu'à chaque fois, ces infections résultaient de contacts sexuels avec un partenaire autre que le partenaire régulier. L'étude Opposites Attract portait sur près de 17 000 actes sexuels sans préservatif entre hommes. Aucune

transmission du VIH n'a été signalée entre les partenaires de l'étude. Trois cas de nouvelle infection par le VIH ont été signalés. Ces nouvelles infections résultaient de contacts sexuels avec un partenaire autre que le partenaire sexuel régulier [18].

Une revue systématique et une méta-analyse de 2013 n'ont pas non plus identifié de transmission lorsque la charge virale était inférieure à un seuil compris entre 50 et 500 copies/ml (selon l'étude) [39]. Une autre étude n'a rapporté aucune transmission lorsque la charge virale était inférieure à 400 copies/mL [40]. Un certain nombre d'autres études ont fourni la preuve qu'une charge virale faible (mais détectable) diminue considérablement (et pourrait éliminer) la possibilité de transmission. Par exemple, les premières études réalisées auprès de participants qui ne prenaient pas de traitement antirétroviral n'ont identifié aucun cas de transmission parmi les couples où l'un des deux partenaires vivait avec le VIH et avait une charge virale faible mais détectable : moins de 1500 copies/ml (Ouganda) [32], moins de 1094 copies/ml (Thaïlande) [33] et moins de 1000 copies/ml (Zambie) [34]. L'étude ougandaise a identifié une probabilité de transmission de 1 sur 10 000 en cas de rapport sexuel vaginal avec une charge virale inférieure à 1700 copies/ml [41]

Alors que de nombreuses personnes, suivant leur traitement, peuvent connaître une augmentation de courte durée de leur charge virale connue sous le nom de « blips » [42,43], cette augmentation ne signifie pas que le traitement « ne fonctionne pas ». Elle n'est pas considérée comme cliniquement significative, et il n'a pas été prouvé qu'elle accroîtrait la possibilité de transmission du VIH dans le cadre de rapports sexuels [44,45]. Les études à grandes échelles menées auprès de couples sérodiscordants ont inclus de nombreux participants ayant connu des « blips » au niveau de leur charge virale au cours des études. Par conséquent, de tels « blips » ont bien été pris en compte dans les réductions observées de la possibilité de transmission du VIH.

- La prophylaxie pré-exposition (PrEP) diminue considérablement la possibilité de contamination par le VIH.
  La prophylaxie pré-exposition se définit comme l'utilisation de médicaments antirétroviraux par des personnes séronégatives avant une exposition au VIH et ce, dans le but de prévenir l'infection [46-50]. Une étude récente a montré que la prophylaxie pré-exposition était efficace jusqu'à 95 % chez les utilisateurs qui

observent le traitement [50]. Cependant, étant donné que seuls quelques cas d'échec chez des patients observants ont été décrits, la prophylaxie pré-exposition est, en réalité, probablement efficace à plus de 95%.

- La prophylaxie post-exposition (PPE) diminue considérablement la possibilité de contamination par le VIH.
  La prophylaxie post-exposition se définit comme l'utilisation, à court terme, d'un traitement antirétroviral par une personne séronégative après avoir été exposée au VIH. Débutée dans les 72 heures suivant l'exposition et prise pendant 28 jours avec une bonne observance, la prophylaxie post-exposition réduit de manière significative la probabilité de devenir séropositif car elle peut empêcher le VIH de s'établir dans les cellules immunitaires même après que le virus est entré dans le corps. [51-52]. Bien que la prophylaxie post-exposition ne soit pas efficace à 100 %, les taux de succès observés sont élevés. [51,53-67] (par ex. , 81 % chez les patients suivant des protocoles de traitements moins récents [67] et jusqu'à 100 % chez les patients utilisant des protocoles plus récents [68]). L'efficacité de la prophylaxie post-exposition semble être influencée par un certain nombre de facteurs, avec une efficacité qui augmente généralement le plus tôt le traitement est démarré et à mesure que la quantité de VIH entrant dans le corps d'une personne diminue [68].

- La circoncision masculine médicale réduit la possibilité de transmission du VIH de la femme à l’homme.
  La circoncision médicale masculine réduit d'environ 50 % la possibilité de transmission du VIH d’une femme séropositive à un homme séronégatif [69]. La circoncision peut également réduire la transmission sexuelle du VIH chez les hommes ayant des rapports sexuels avec d’autres hommes si le partenaire séronégatif est uniquement le partenaire pénétrant, bien que les études ne soient pas concluantes [70].

- Les pratiques de réduction des risques telles que le retrait ou le positionnement stratégique diminuent la possibilité de transmission du VIH.
  Certaines personnes vivant avec le VIH ont recours à des pratiques de réduction des risques telles que le retrait avant l'éjaculation ou le positionnement stratégique (c.-à-d. dans la position de partenaire pénétré uniquement) en cas des rapports sexuels sans préservatif avec une personne séronégative ou de statut inconnu [71-73]. De telles pratiques réduisent la possibilité de transmission du VIH pendant l’acte sexuel

lorsqu'une telle possibilité existe [71]. Par exemple, une étude réalisée en 2010 a révélé que la probabilité de transmission associée au sexe anal diminuait de deux tiers environ si le partenaire pénétrant séropositif n'éjaculait pas [73]. La possibilité de transmission est également connue pour être plus faible quand le partenaire séropositif est le partenaire pénétré plutôt que le partenaire pénétrant, dans le cadre d’un rapport sexuel anal [73-75].

- Les infections sexuellement transmissibles (IST) peuvent augmenter la possibilité de transmission du VIH dans certaines circonstances.
  La présence de certaines IST non traitées, en particulier des IST ulcéreuses, chez l'un ou l'autre des partenaires a été associée à une probabilité accrue de transmission sexuelle du VIH lorsque le partenaire séropositif n’a pas de charge virale faible [76]. Lorsque les deux partenaires ont des ulcères génitaux, le risque est encore accru [14]. Cependant, la présence d'une infection sexuellement transmissible n'augmente pas la possibilité de transmission lorsque le partenaire séropositif est sous traitement antirétroviral efficace [30], *ou* que le partenaire séronégatif prend la PrEP [48,49].

Possibilité de transmission du VIH par voie sexuelle

La transmission du VIH par voie sexuelle se produit généralement à la suite d’une mise en contact entre des fluides corporels contenant suffisamment de VIH et les muqueuses situées dans : le prépuce ou l'urètre du pénis ; le col de l'utérus ou le vagin ; l'anus ; ou le rectum. La transmission du VIH est également possible par contact avec les muqueuses buccales, mais celles-ci sont beaucoup moins vulnérables à la transmission du VIH [58].

Sexe oral, y compris le sexe oral-pénien et le sexe oral-vaginal

- La possibilité de transmission du VIH en cas de sexe oral pratiqué sur une personne séropositive y compris lorsque sa charge virale n'est pas faible et en l’absence de préservatif varie de nulle à négligeable selon le contexte [77,78].

Le sexe oral est promu comme une pratique sexuelle plus sûre pour les partenaires sérodiscordants souhaitant avoir des rapports sexuels intimes, et sa pratique serait très courante.

La possibilité de transmission en cas de sexe oral est connue pour être beaucoup plus faible que la possibilité de transmission associée aux rapports sexuels vaginaux ou anaux [79,80]. En fait, le risque de transmission du VIH associé au sexe oral est tellement faible que les

scientifiques n'ont pas été en mesure de donner une estimation du risque de transmission statistiquement fiable.

Les quelques études cliniques portant sur la transmission du VIH par voie orale n'ont trouvé aucun cas de transmission [74,81,82]. Une étude portant sur des couples hétérosexuels et une étude portant sur des couples de lesbiennes n'ont identifié aucune transmission résultant de rapports sexuels oraux [81,82]. Une troisième étude portant sur des hommes ayant des rapports sexuels avec d'autres hommes n'a montré aucune séroconversion parmi les participants ayant déclaré pratiquer uniquement des fellations (avec éjaculation) sur des hommes séropositifs ou dont le statut VIH n'était pas connu [74]. Un modèle statistique appliqué à ces résultats a conclu que le risque, par contact, associé au sexe oral se situait entre zéro et 0,04 % (4 sur 10 000) [78]. Ces chiffres sont référencés dans certains rapports [79,80,83] Compte tenu du fait que l'étude n'a identifié aucune séroconversion, la limite supérieure de 0,04 % peut être comprise comme une limite supérieure de possibilité.

- Il n'y a aucune possibilité de transmission du VIH en cas de sexe oral pratiqué sur une personne séropositive lorsque le partenaire séropositif a une charge virale faible **ou** qu'un préservatif est correctement utilisé **ou** que le partenaire séronégatif prend la PrEP [78].

Bien qu'il n'existe pas d'étude sur l'effet du traitement antirétroviral ou de la prophylaxie pré-exposition sur la possibilité de transmission lors de rapports sexuels oraux, notre opinion d'expert est qu'il n'existe aucune possibilité de transmission du VIH associée au sexe oral pratiqué sur un partenaire séropositif sous traitement antirétroviral, ou pratiqué par partenaire prenant la PrEP. De même, l'utilisation correcte du préservatif réduit la probabilité de transmission du VIH à zéro.

Rapports sexuels vaginaux-péniens

- La possibilité de transmission du VIH en cas de rapports sexuels vaginaux-péniens sans préservatif **et** alors que le partenaire séropositif n'a pas de charge virale faible, est faible [84]. La probabilité de transmission est encore plus faible en l'absence d'éjaculation à l'intérieur du partenaire séronégatif.

Deux méta-analyses de couples hétérosexuels [14,84] ont montré que la probabilité de transmission du VIH associée à un rapport sexuel vaginal est faible : 0,08 % (8 sur 10 000) en l'absence de cofacteurs de risque [5,14,41,84]. Il n'est pas clair que la probabilité de

transmission du VIH d'un homme à une femme lors d'un rapport sexuel vaginal soit plus élevée que la transmission d'une femme à un homme. Certaines études n'ont trouvé aucune différence, tandis que d'autres suggèrent que la possibilité de transmission du VIH d'un homme à une femme est deux fois plus élevée que la possibilité de transmission d'une femme à un homme [14,35,83,84].

- La possibilité de transmission du VIH en cas de rapports sexuels vaginaux-péniens lorsque le partenaire séropositif a une charge virale basse **ou** utilise un préservatif **ou** que le partenaire séronégatif prend la PrEP varie de nulle à négligeable selon le contexte [29,38].

De nombreuses études, comme discuté ci-dessus, ont montré que la possibilité de transmission du VIH en cas de rapports sexuels vaginaux-péniens varie de nulle à négligeable lorsque le partenaire séropositif a une charge virale faible [29,37-39,85]. De même, aucun cas de transmission en cas de rapports sexuels vaginaux-péniens n'a jamais été signalé dans aucun essai clinique en présence d'une charge virale indétectable.

Le VIH ne peut pas se transmettre quand un préservatif est utilisé correctement parce que le VIH ne peut pas passer à travers le latex ou le polyuréthane intact. De même, il n'y a aucune possibilité de transmission du VIH lorsqu'une personne a une charge virale indétectable.

Rapports sexuels anaux-péniens

- La possibilité de transmission du VIH en cas de rapport sexuels anaux-péniens sans préservatif **et** alors que le partenaire séropositif n'a pas de charge virale faible, est faible que le partenaire pénétré séronégatif soit un homme ou une femme [86]. La probabilité de transmission est plus faible lorsque le partenaire séropositif est celui qui est pénétré plutôt que celui qui pénètre son partenaire. Elle est également plus faible si la personne séropositive pénétrant son partenaire n'éjacule pas à l'intérieur de son partenaire.

Des études montrent que les rapports anaux « réceptifs » sans préservatif de couples hétérosexuels ou homosexuels sont associés à une probabilité plus élevée de transmission du VIH que les rapports vaginaux « réceptifs » sans préservatif [5,87,88]. Des études individuelles ont estimé une probabilité de transmission du VIH associée à un rapport sexuel anal allant de 0,01 % (1 sur 10 000) à plus de 3 % (300 sur 10 000) [20,75,84,88-91]. La probabilité de transmission du partenaire pénétrant au partenaire pénétré est plus élevée que le contraire [18,75,84].

Deux revues systématiques (2010 et 2014) rapportent une estimation par acte d'environ 1,4 % (140 sur 10 000) pour les relations sexuelles anales « réceptives » (c.-à-d. lorsque la personne séropositive est le partenaire pénétrant) [5,86]. Une étude de cohorte prospective de 2010 a montré que la probabilité tombe de 1,43 % (143 pour 10 000) avec éjaculation à 0,54 % (54 pour 10 000) sans éjaculation [89]. La probabilité de transmission par acte a été estimée à 0,11 % (11 sur 10 000) lorsque la personne séronégative est le partenaire pénétrant [5].

- La possibilité de transmission du VIH en cas de rapports sexuels anaux-péniens lorsque le partenaire séropositif a une charge virale basse **ou** utilise un préservatif **ou** que le partenaire séronégatif prend la PrEP varie de nulle à négligeable selon le contexte. La probabilité est la même que le partenaire séronégatif pénétré soit un homme ou une femme. [85,86].

La possibilité de transmission du VIH, en cas de rapports sexuels anaux-péniens, est négligeable lorsque le partenaire séropositif a une charge virale basse. Comme indiqué ci-dessus, l'étude PARTNER et l'étude Opposites Attract n'ont observé aucune transmission de VIH après environ 39 000 actes de sexe anal sans préservatif et avec une charge virale inférieure à 200 copies / ml [30,92]. En fait, aucun cas de transmission n'a jamais été signalé dans aucun essai clinique en présence d’une charge virale indétectable.

Le VIH ne peut pas se transmettre quand un préservatif est utilisé correctement parce que le VIH ne peut pas passer à travers le latex ou le polyuréthane intact. De même, il n'y a aucune possibilité de transmission du VIH lorsqu'une personne a une charge virale indétectable.

Possibilité de transmission du VIH par simple contact, crachement et morsure

Simples contacts

Le VIH ne se transmet pas au contact de surfaces exposées à l’air libre telle qu'une chaise, un banc ou des toilettes ; de la nourriture ou de la boisson ; ou à travers un simple contact humain comme se serrer dans les bras, partager des objets ménagers ou manger ensemble.

Le VIH ne peut pas survivre longtemps dans l'air et il est incapable de pénétrer la peau intacte. Aucun cas d'infection au VIH par contact avec une surface, un aliment ou une boisson, ou par simple contact humain n'a jamais été identifié malgré de nombreuses études scientifiques envisageant cette possibilité [93-98].

Morsures et crachements

- Il n'y a aucune possibilité de transmission du VIH par contact avec la salive d'une personne séropositive, que ce soit au cours de baisers, à la suite d'une morsure ou d'un crachement.

De nombreuses études ont examiné la possibilité de transmission du VIH à travers la salive, mais aucune n'a trouvé de preuve de transmission, y compris une étude de 1997 portant sur 34 000 cas d'exposition au Royaume-Uni [99]. L'absence de transmission du VIH à travers la salive est attribuée à deux facteurs : la salive contient une très petite quantité de VIH [100], et l'existence de plusieurs composants inhibiteurs dans les sécrétions orales permet à la salive de protéger des cellules sensibles contre l'infection au VIH [101-106].

- Il n'y a aucune possibilité de transmission du VIH en cas de morsure ou de crachement lorsque la salive d'une personne séropositive ne contient pas ou peu de sang.

Les données actuelles suggèrent que le VIH ne peut pas non plus se transmettre lorsque la salive contient de petites quantités de sang. Malgré des recherches préliminaires suggérant un risque théorique de transmission dans le cas où la salive (contenant du sang) entrerait dans le corps d'une personne à travers le tissu muqueux (par exemple, en atterrissant dans l'œil ou la bouche), aucun cas de transmission du VIH n'a été rapporté [107]. Par conséquent, notre opinion d'expert est qu'il n'y a aucune possibilité de transmission du VIH à travers la salive contenant de petites quantités de sang.

- La possibilité de transmission du VIH en cas de morsure lorsque la salive d'une personne séropositive contient une quantité importante de sang, que le sang entre en contact avec une membrane muqueuse ou une plaie ouverte **et** que la charge virale de la personne séropositive n'est ni basse ni indétectable varie de nulle à négligeable.

De nombreuses études ont présenté un grand nombre de cas de morsures n'ayant entraîné aucune transmission du VIH [108-112] ou ont estimé que la transmission était improbable [107,109,113,114].

Pour que la transmission soit plausible en cas de morsure, la personne séropositive doit avoir du sang dans sa bouche au moment de la morsure, une quantité suffisante de VIH dans son sang, et la morsure doit être suffisamment profonde et pénétrer la peau de la personne séronégative, causant des traumatismes et des lésions tissulaires [106,107,115]. Même lorsque toutes ces conditions sont réunies, la possibilité de transmission lors d'un seul cas de morsure est tout au plus négligeable.

Améliorations significatives de l'espérance de vie et de la qualité de vie des personnes vivant avec le VIH

La deuxième section de cette déclaration de consensus examine le préjudice associé à l'infection du VIH parce que certaines idées fausses qui persistent dans nos sociétés exagèrent les conséquences de l'infection et semblent influencer l'application du droit pénal [3]. Le droit pénal prend en compte, non seulement, la probabilité que l'infraction se matérialise mais aussi le préjudice qui pourrait résulter de l'infraction. Ainsi, par exemple, les éléments constitutifs de l'infraction de « lésions corporelles » sont différents de ceux de l'infraction de « lésions corporelles graves », qui se distinguent de ceux de l' « homicide involontaire » ou du « meurtre ». C'est pourquoi, il est important de souligner les énormes changements en termes de perspectives d'avenir pour les personnes vivant avec le VIH qui ont eu lieu au cours des dernières décennies.

L'évolution naturelle de l'infection par le VIH non traitée varie considérablement d'une personne à l'autre [116]. Si l'infection n'est pas traitée, la plupart des personnes connaissent une phase asymptomatique qui dure de deux à 15 ans, au cours de laquelle le virus se reproduit, sapant progressivement leur système immunitaire. Un petit pourcentage de personnes infectées par le VIH ont des systèmes immunitaires qui bloquent la réplication du virus pour une durée indéterminée [117], mais la grande majorité des personnes finissent par développer le sida si elles ne sont pas traitées (environ la moitié des personnes dans une période de 10 ans [118]). Le sida se définit par la présence de marqueurs de laboratoire spécifiques ou d'infections opportunistes et de maladies spécifiques qui, si la thérapie antirétrovirale n'est pas entamée, finissent par entraîner la mort.

Les thérapies antirétrovirales réduisent considérablement la progression de l'infection par le VIH vers le sida. Globalement, les recommandations thérapeutiques ont été révisées pour recommander de commencer un traitement antirétroviral immédiatement après le diagnostic de l'infection au VIH car la plupart des personnes sous traitement auront une charge virale indétectable, garderont un système immunitaire sain, resteront en bonne santé et éviteront les complications de l'infection à VIH à long terme [119-120]. Même ceux qui commencent un traitement - alors que leur charge virale est élevée - peuvent s'attendre, en suivant leur traitement, à une réduction étonnante de leur charge virale au point de récupérer un système immunitaire leur permettant de jouir d'une bonne santé à long terme [121]. Pour beaucoup, un traitement efficace requiert une seule pilule par jour.

Des études menées dans de nombreux pays ont montré que les traitements antirétroviraux ont radicalement augmenté l'espérance de vie, que l'espérance de vie a continué à s'améliorer avec le temps et que la santé et la qualité de vie, à long terme, des personnes vivant avec le VIH se sont considérablement améliorées [122-141]. L'espérance de vie de jeunes séropositifs qui commencent aujourd'hui un traitement antirétroviral approche celle des jeunes de la population générale [45,132,134,135,137]. En outre, avec les thérapies antirétrovirales les causes de décès des personnes vivant avec le VIH sont passées de maladies liées au sida à des causes non liées au VIH [142,143] et similaires à celles qui touchent la population générale [144]. De même, la prise en charge clinique des patients a évolué pour inclure maintenant le traitement de problèmes de santé liés au vieillissement, comme la ménopause, et les maladies cardiovasculaires [143-150], et des interventions visant à influencer les « modes de vie » comme le tabagisme [151]. Dans certaines sous-populations, les soins cliniques en cours ont le potentiel d'augmenter l'espérance de vie des personnes vivant avec le VIH au-delà de celle de leurs homologues séronégatifs [135].

Bien que le VIH provoque une infection nécessitant un traitement continu avec des antirétroviraux, les personnes vivant avec le VIH peuvent mener une vie longue et productive, travailler, étudier, et voyager. Elles peuvent avoir des relations amoureuses, faire et élever des enfants et contribuer à la vie sociale de bien d'autres manières.

Établir une preuve de transmission du VIH

La dernière section de cette déclaration de consensus reconnaît l'importance d'utiliser correctement les données scientifiques et médicales dans les cas de poursuites pénales liées au VIH où la transmission effective du VIH est en cause.

Les directives internationales sur le VIH dans le contexte du droit pénal recommandent que « la preuve du lien de causalité, en matière de transmission du VIH, soit toujours fondée sur des preuves provenant de plusieurs sources pertinentes, incluant notamment les dossiers médicaux, des méthodes scientifiques rigoureuses et des informations relatives aux antécédents sexuels » [1].

- Les dossiers médicaux peuvent fournir des informations contextuelles mais ne permettent pas d'établir une transmission entre un plaignant et un défendeur.

Les circonstances liées à la nature de la relation sexuelle, au moment où celle-ci a eu lieu, ainsi que les informations relatives à d'autres sources potentielles d'infection sont des éléments fondamentaux qui doivent être pris en considération dans tous les cas où la transmission sexuelle du VIH est alléguée. Lorsqu'ils sont disponibles et légalement obtenus,

les dossiers médicaux peuvent être utiles pour déterminer la date des derniers tests négatifs et des premiers tests positifs au VIH du plaignant et du défendeur. En prenant en compte la période de latence sérologique associée à chaque test, cette information peut être utilisée pour identifier la période au cours de laquelle le plaignant a contracté le VIH et savoir si le prévenu était séropositif à ce moment là. Fait important : le fait d'avoir fait le test de dépistage en premier ou d'avoir été la personne à porter les accusations, ne permet pas, de déterminer qui, du plaignant ou du défendeur, était le premier à être infecté.

Les informations relatives à la charge virale du VIH et au nombre de CD4 présentes dans les dossiers médicaux ont parfois été présentées comme des éléments de preuve permettant d'établir le moment de l'infection. Cependant, la charge virale et le nombre de CD4 varient considérablement autant au niveau inter qu'intra-individuel et ne peuvent donc pas être utilisés pour déterminer exactement quand quelqu'un a contracté le VIH [152].

- L'analyse phylogénétique peut être utilisée comme un outil médico-légal. Les résultats peuvent être compatibles avec l'allégation selon laquelle un défendeur aurait infecté un plaignant, mais ils ne peuvent pas le prouver de façon concluante. Fait important, les résultats phylogénétiques peuvent disculper un accusé lorsque les résultats ne sont pas compatibles avec l'allégation selon laquelle le défendeur aurait infecté le plaignant.

L'analyse phylogénétique compare la relation évolutive du VIH chez différentes personnes, mais les résultats doivent être interprétés avec prudence lorsqu'ils sont utilisés dans les affaires pénales en complément d'autres éléments de preuves factuelles et médicales [153]. La complexité de l'analyse phylogénétique est en partie liée au fait que le VIH est un virus qui évolue rapidement. Le virus est sujet à des mutations répétées, de sorte que chaque personne vivant avec le VIH a plus d'une variante du virus [154]. Au cours de la transmission, un nombre limité de variantes du virus (entre une et quelques variantes) seront transmises, mais celles-ci vont de nouveau muter pour former de nouvelles variantes de sorte que le virus du VIH entre deux personnes n'est jamais identique [155].

L'analyse phylogénétique du VIH consiste à évaluer les relations évolutives entre différentes variantes du VIH dans le but, par exemple, d'étudier les réseaux de transmission du VIH à des fins de santé publique. Dans les affaires pénales, l'analyse phylogénétique consiste à déterminer si le ou les plaignants et le ou les défendeurs font bien partie d'un même réseau de transmission. Le réseau est représenté comme un " arbre " phylogénétique. Il convient de noter que l'arbre phylogénétique doit être compris comme un arbre génétique du VIH. Cet arbre peut différer de l'historique de la transmission car les variantes du VIH peuvent

précéder la transmission ou disparaître après la transmission [156] et parce que certaines personnes d’un même réseau de transmission peuvent ne pas avoir été diagnostiquées ou avoir été échantillonnées avant la construction de l'arbre.

La phylogénétique du VIH est très différente du profilage de l'ADN humain car, étant donné l'évolution continue des variantes du VIH pour chaque personne, la phylogénétique ne peut pas obtenir de « correspondance exacte ». Quand il semble y avoir une « correspondance phylogénétique » entre le VIH de deux individus, cela signifie que deux variantes ou plus sont épidémiologiquement « liées », et non pas qu'elles sont identiques [155,157]. En revanche la preuve phylogénétique du VIH *peut* exonérer une personne accusée d’avoir transmis le VIH car si les souches de virus détectées, à la fois chez le défendeur et le plaignant, ne sont pas liées, alors les preuves phylogénétiques contredisent effectivement et de manière concluante l’allégation selon laquelle que le défendeur serait à la source du virus du plaignant. [155,158].

Des avancées récentes dans le séquençage de l'ADN et la phylogénétique permettent désormais de tenir compte du sens et du moment de la transmission [159-162]. Toutefois, ces méthodes ne sont actuellement ni suffisamment détaillées, ni suffisamment précises pour prouver qui a infecté qui [155,163]. C'est en partie parce qu'il peut toujours y avoir des individus inconnus et non diagnostiqués au sein du réseau de transmission [155]. Par conséquent, à l’heure actuelle, l'analyse phylogénétique ne peut pas permettre d’éliminer la possibilité que le plaignant ait lui-même infecté le défendeur, que les deux aient été infectés par un tiers [158,163] ou que d’autres scénarios plus complexes de transmission expliquent pourquoi les variantes de VIH du défendeur et du plaignant sont liées épidémiologiquement. Le fait que le VIH ne protège pas contre une « super » infection ultérieure avec une variante différente ajoute encore de la complexité [158]. En particulier, la fiabilité des informations portant sur le sens de l'infection est compromise lorsque le défendeur et le plaignant se sont livrés à de nombreux actes sexuels, ce qui a peut-être facilité de multiples contaminations et recontaminations [155].

L'analyse phylogénétique est complexe, et par conséquent, il est important que la phylogénétique du VIH à des fins médico-légales soit effectuée et interprétée par des experts qui en comprennent pleinement les limites et les énoncent explicitement dans leurs rapports écrits et au cours de leurs témoignages. L'interprétation des résultats phylogénétiques à des fins médico-légales nécessite une expertise en phylogénétique et dans la distinction entre les arbres évolutifs de virus et les historiques de transmission. Cette interprétation n'est pas aisée et les méthodologies n'ont pas encore été standardisées [155]. La fiabilité des données dérivées de

l'analyse phylogénétique dépend d'un certain nombre de facteurs méthodologiques, incluant l'utilisation de « contrôles locaux » adéquats [164-166] et de séquences de bases de données [167-169], qui doivent être sélectionnés sur la base de critères de sélection uniformes [155]. La recherche, au niveau international, montre que les preuves phylogénétiques utilisées dans les procès pénaux n'ont pas toujours satisfait à ces exigences [155].

Conclusion

Compte tenu des éléments présentés dans ce document, nous recommandons fortement de faire preuve de plus de prudence lorsqu'une poursuite pénale est envisagée, et notamment, d'évaluer minutieusement les dernières données scientifiques sur les risques de transmission et les conséquences de l'infection. Ceci est essentiel pour réduire la stigmatisation, la discrimination et éviter les erreurs judiciaires.

Dans ce contexte, nous espérons que cette déclaration de consensus encouragera les gouvernements et les acteurs de la justice à prêter une attention particulière aux importantes avancées scientifiques réalisées au cours des trente dernières années dans le domaine du VIH et de faire tout ce qui est en leur pouvoir pour garantir que toute application du droit pénal, dans les affaires relatives au VIH, soit fondée sur une compréhension exacte et complète des données scientifiques probantes actuelles.

Intérêts concurrents

Peter Godfrey-Faussett est employé à la London School of Hygiene and Tropical Medicine et détaché à plein temps à l'ONUSIDA. Tous les autres auteurs déclarent qu'ils n'ont aucun intérêt concurrent.

Remerciements

Nous remercions Sally Cameron, Edwin Bernard, Luisa Cabal, Stéphanie Claivaz-Loranger, Patrick Eba, Richard Elliott, Cécile Kazatchkine, David McLay, Kevin Osborne, Mariangela Simão, et Laurel Sprague

Financement

Ce travail a été soutenir par le financement de International AIDS Society (IAS), International Association of Providers of AIDS Care (IAPAC), le Robert Carr Fund pour les réseaux de la société civile et l'ONUSIDA.

Contribution des auteurs

Tous les auteurs ont participé à de nombreux cycles de discussion, rédaction et révision de cette déclaration de consensus.

Références

1. Programme commun des Nations Unies sur le VIH / sida (ONUSIDA). Note d'orientation de l'ONUSIDA sur l'éradication de la criminalisation trop générale du VIH. Genève: ONUSIDA; 2013.
2. Commission mondiale sur le VIH et la loi. VIH et la loi : Risques, droits et santé. Genève: PNUD; 2012.
3. Bernard EJ, Cameron S. Advancing HIV Justice 2: Building momentum in global advocacy against HIV criminalization. HIV Justice Network, Global Network of People Living with HIV; Avril 2016.
4. Organisation mondiale de la santé. Santé sexuelle, droits de l'homme et Loi. Genève: Organisation mondiale de la santé; 2015.
5. Patel P, Borkowf CB, Brooks JT, Lasry A, Lansky A, Mermin J. Estimating per-act HIV transmission risk: a systematic review. AIDS. 2014 Juin 19 ; 28 (10) : 1509-19.
6. Centers for Disease Control and Prevention. CDC Information Undetectable Viral Load and HIV Transmission Risk. Octobre 2017. Disponible à l'adresse https://www.cdc.gov/hiv/pdf/risk/art/cdc-hiv-uvl-transmission.pdf
7. 2008 MBQB 201, Canada.
8. R v Mabior. SCC 47. 2012; Canada.
9. Boyd M, Cooper D, Crock E, Crooks L, Giles M, Grulich A, et al. Sexual transmission of HIV and the law: an Australian medical consensus statement. Med J Aust. 2016; 205 (9) : 409-12.
10. Loutfy M, Tyndall M, Baril J-G, Montaner J, Kaul R, Hankins C. Canadian consensus statement on HIV and its transmission in the context of the criminal law. Can J Infect Dis Med Microbiol. 2014;25(3) :135-40.
11. Albert J, Berglund T, Gisslén, M, Gröön P, Sönnerborg A, Tegnell A, et al. Risk of HIV transmission from patients on antiretroviral therapy: a position statement from the Public Health Agency of Sweden and the Swedish Reference Group for Antiviral Therapy. Scandinavian Journal of Infectious Diseases. 2014;46(10):673-7.
12. Vernazza P, Hirschel B, Bernasconi E, Flepp M. Les personnes séropositives ne souffrant d'aucune autre MST et suivant un traitment antirétroviral efficace ne transmettent pas le VIH par voie sexuelle. Bulletin des médecins suisses. 2008 30; 89 (5), 165-9. Français.
13. Vandamme AM, Van Laethem K, JC Schmit, Van Wijngaerden E, Reynders M, Debyser Z, et al. Long-term stability of human immunodeficiency virus viral load and infectivity in whole blood. European Journal of Clinical Investigation. 1999; 29:445-52.
14. Powers K, Poole C, Pettifor A, Cohen M. Rethinking the heterosexual infectivity of HIV-1: a systematic review and meta-analysis. Lancet Infect Dis. Septembre 2008; 8 (9): 553-63.
15. Lytle CD. An in vitro evaluation of condoms as barriers to a small virus. Sex Transm Dis. 1997;24 :161-4.
16. Vittinghoff E, Douglas J, Judson F, McKirnan D, MacQueen K et Buchbinder SP. Per-contact risk of human immunodeficiency virus transmission between male sexual partners. Am J Epidemiol. 1er août 1999; 150 (3): 306-11.
17. Weller S, Davis K. Condom effectiveness in reducing heterosexual HIV transmission. Base de données Cochrane Syst Rev. [internet]. 2001 [cité le 6 juin 2018] Disponible à: http ://onlinelibrary.wiley.com

18. Macdonald N, Elam G, F Hickson, Imrie J, CA McGarrigle, Fenton KA, et al. Factors associated with HIV seroconversion in gay men in England at the start of the 21st century. Sex Transm Infect. Fév 2008; 84(1): 8-13.

19. Lavoie E, Alary M, Remis RS, Otis J, Vincelette J, Turmel B. Determinants of HIV seroconversion among men who have sex with men living in a low HIV incidence population in the era of highly active antiretroviral therapies. Sex Transm Dis. Janvier 2008; 35(1): 25-9.

20. Scott HM, Vittinghoff E, R Irvin, Sachdev D, Liu A, Gurwith M et al. Age, Race/Ethnicity, and Behavioral Risk Factors Associated with Per-Contact Risk of HIV Infection Among Men Who Have Sex with Men in the United States. J Acquir Immune Defic Syndr. 1er janvier 2014; 65 (1): 115-121.

21. Smith DK, Herbst JH, Zhang X, Rose CE. Condom effectiveness for HIV prevention by consistency of use among men who have sex with men in the United States. J Acquir Immune Defic Syndr. 1er mars 2015; 68 (3): 337-44. Référencé dans doi : 10.1097 / QAI.0000000000000461.

22. Weller S, Davis-Beaty K. Condom effectiveness in reducing heterosexual HIV transmission. Base de données Cochrane Syst Rev 2002; (1): CD003255.

23. Crosby R, Bounse S. Condom effectiveness: where are we now?. Sex Health. 2012; 9 : 10-17

24. Buchbinder S, Katz M, Hessol N, O'Malley P, Homberg S. Long-term HIV-1 infection without immunologic progression. AIDS. 1994; 8 :1123-8.

25. Madec Y, F Boufassa, Avettand-Fenoel V, S Hendou, Melard A, Boucherit S, et al. Early control of HIV-1 infection in long-term nonprogressors followed since diagnosis in the ANRS SEROCO/HEMOCO cohort. J Acquir Immune Defic Syndr. 2009; 50 :19-26.

26. Poropatich K, Sullivan DJ. Human immunodeficiency virus type 1 long-term non-progressors: the viral, genetic and immunological basis for disease non-progression. Journal de la virologie générale. 2010;2(2) :247-68.

27. Learmont J, Geczy A, Mills J, Ashton L, C Raynes-Greenow, R Garsia, et al. Immunologic and virologic status after 14 to 18 years of infection with an attenuated strain of HIV-1. A report from the Sydney Blood Bank Cohort. N Engl J Med 1999; 340 : 1715-22.

28. Rhodes DI, Ashton L, Salomon A, Carr A, Cooper D, Kaldor J, et al. Characterization of three nef-defective human immunodeficiency virus type 1 strains associated with long-term nonprogression. Australian Long-Term Nonprogressor Study Group. J. Virol. Novembre 2000 : 74 (22) : 10581-8.

29. Cohen MS, Chen YQ, McCauley M, Gamble T, Hosseinipour MC, Kumarasamy N, et al. Prevention of HIV-1 infection with early antiretroviral therapy. N Engl J Med. 11 août 2011; 365 : 493-505.

30. Rodger AJ, Cambiano V, Bruun T, Vernazza P, Collins S, van Lunzen J, et al. Sexual activity without condoms and risk of HIV transmission in serodifferent couples when the HIV-positive partner is using suppressive antiretroviral therapy. JAMA. 2016; 316 :171-81.

31. Montaner JS, Hogg R, Bois E, Kerr T, M Tyndall, Levy AR, et al. The case for expanding access to highly active antiretroviral therapy to curb the growth of the HIV epidemic. Lancet. 2006;368(9534) :531-6.

32. Quinn TC, Wawer MJ, N Sewankambo, Serwadda D, Li C, Wabwire-Mangen F, et al. Viral load and heterosexual transmission of human immunodeficiency virus type 1. Groupe d'étude du projet Rakai. N Engl J Med. 2000;342(13) :921-9.

33. Tovanabutra S, Robison V, Wongtrakul J, Sennum S, Suriyanon V, Kingkeow D, et al. Male viral load and heterosexual transmission of HIV-1 subtype E in northern Thailand. J Acquir Immune Defic Syndr. 1er mars 2002 ; 29 (3) : 275-83.

34. Fideli US, Allen SA, Musonda R, Trask S, Hahn BH, Weiss H, et al. Virologic and immunologic determinants of heterosexual transmission of human immunodeficiency virus type 1 in Africa. AIDS Res Hum Retroviruses. 1er juil. 2001; 17 (10) : 901-10.

35. Hughes JP, Baeten JM, Lingappa JR, Magaret AS, Wald A, de Bruyn G. Determinants of per-coital-act HIV-1 infectivity among African HIV-1-serodiscordant couples. J Infect Dis. 1er février 2012 ; 205 (3) : 358-65.

36. Grulich A, B Bavinton, Jin F, Prestage G, Zablotska, Grinsztejn B, et al. HIV transmission in male serodiscordant couples in Australia, Thailand and Brazil. Résumé de la conférence 2015 sur les rétrovirus et les infections opportunistes, Seattle, États-Unis, 2015.

37. MS Cohen, Chen YQ, M McCauley, Gamble T, M Hosseinipour, N Kumarasamy, et al. Antiretroviral Therapy for the Prevention of HIV-1 Transmission. N Engl J Med. 1er septembre 2016 ; 375 (9) : 830-9.

38. Supervie V, Viard J-P, Costagliola D, Breban R. Heterosexual risk of HIV transmission per sexual act under combined antiretroviral therapy: systematic review and Bayesian modeling. Clin Infect Dis Off Publ Infect Dis Soc Am. 2014 Jul 1;59(1):115-22.

39. Loutfy MR, W Wu, Letchumanan L, Bondy L, Antoniou T, Margolese S, et al. Systematic Review of HIV Transmission between Heterosexual Serodiscordant Couples where the HIV Positive Partner Is Fully Suppressed on Antiretroviral Therapy. PLoS ONE. 13 février 2012 ; 8 (12).

40. Anglemyer A, Rutherford GW, Baggaley RC, Egger M, Siegfried N. Antiretroviral therapy for prevention of HIV transmission in HIV-discordant couples. Base de données Cochrane Syst. Rev. 30 Avril 2013 ; (4).

41. Mastro TD, de Vincenzi I.Probabilities of sexual HIV-1 transmission. AIDS.1996.10 Suppl A : S75-82.

42. Gray RH, Wawer MJ, Brookmeyer R, Sewankambo NK, Serwadda D, Wabwire-Mangen F, et al. Probability of HIV-1 transmission per coital act in monogamous, heterosexual, HIV-1-discordant couples in Rakai, Uganda. Lancet. 14 avril 2001 357 (9263) : 1149-53.

43. Young J, Rickenbach M, Calmy A, Bernasconi E, C Staehelin, Schmid P, et al. Transient detectable viremia and the risk of viral rebound in patients from the Swiss HIV Cohort Study. BMC Infect Dis. 2015 ; 15 (1) : 382.

44. Sörstedt E, Nilsson S, Blaxhult A, Gisslén M, Flamholc L, Sönnerborg A, et al. Viral blips during suppressive antiretroviral treatment are associated with high baseline HIV-1 RNA levels. BMC Infectious Diseases. 2016; 16 : 305.

45. Van Sighem A, Zhang S, P Reiss, Gras L, M van der Ende, Kroon F, et al. Immunologic, virologic, and clinical consequences of episodes of transient viremia during suppressive combination antiretroviral therapy. J Acquir Immune Defic Syndr. 1er mai 2008 ; 48 : 104-8.

46. Teira R, F Vidal, P Muñoz-Sánchez, P Geijo, Viciana P, Ribera E, et al. Very low level viraemia and risk of virological failure in treated HIV-1-infected patients. HIV Medicine, édition en ligne. HIV Med. Mars 2017 ;18(3) :196-203.

47. Fonner V, Dalglish S, Kennedya C, Baggaley R, O'Reilly K, Koechlinb F, et al. Effectiveness and safety of oral HIV preexposure prophylaxis for all populations. AIDS. 2016;30 :1973-83.

48. Molina JM, Capitant C, Spire B, Pialoux G, Cotte L, Charreau I, et al. On-Demand Preexposure Prophylaxis in Men at High Risk for HIV-1 Infection. N Engl J Med. 3 dec 2015 ;373 :2237-46.

49. McCormack, S, Dunn D, Desai M, Dolling D, Gafos M, Gilson R, et al. Pre-exposure prophylaxis to prevent the acquisition of HIV-1 infection (PROUD): effectiveness results from the pilot phase of a pragmatic open-label randomised trial. The Lancet. Sept 9 2015 ;387(10013) :53-60.

50. Grant RM, Liegler T, Defechereux P, Kashuba AD, Taylor D, Abdel-Mohsen M, et al. Drug resistance and plasma viral RNA level after ineffective use of oral pre-exposure prophylaxis in women. AIDS. 2015; 29 :331-7.

51. Schechter M, do Lago R, Mendelsohn A, Moreira R, Moulton L, Harrison L, et alBehavioral impact, acceptability, and HIV incidence among homosexual men with access to postexposure chemoprophylaxis for HIV. JAIDS. 15 april 2004 ;35(5) :519-25.

52. Pinkerton SD, Martin J, Roland M, Katz M, Coates T, Kahn J, et al. Cost-effectiveness of postexposure prophylaxis after sexual or injection-drug use exposure to human immunodeficiency virus. Arch Intern Med. 2004; 164 :46-54.

53. Jochimsen EM. Échecs de zidovudine après la prophylaxie. Am J Med. 19 mai 1997 ;102(5) Sup2 :52-55.

54. Lot F, Abiteboul D. Occupational infections with HIV in France among health-care personnel. Bull Epi Hebdom. 1999;18:69-70. French.

55. Beltrami EM, Luo CC, de la Torre N, Cardo DM. Transmission of drug-resistant HIV after an occupational exposure despite postexposure prophylaxis with a combination drug regimen. Infect Control Hosp Epidemiols. 2002;23 :345-48, 2002.

56. Hawkins DA, Asboe D, Barlow K, Evans B. Seroconversion to HIV-1 following a needlestick injury despite combination post-exposure prophylaxis. J Infect. 2001; 43 :12-15.

57. Wulfsohn A, Venter WDF, Schultze D, M Levey, Sanne IM.. Post-exposure prophylaxis after sexual assault in South Africa. Actes de la dixième Conférence sur les rétrovirus et les infections opportunistes; février 2003; Boston, États-Unis : résumé 42.

58. Lunding S et al. Danish postexposure prophylaxis (PEP) registry: use and failure of antiretroviral chemoprophylaxis following sexual exposure to HIV. Seizième Conférence internationale sur le sida, Toronto, résumé TUPE0433, 2006.

59. Donnell D, Mimiaga MJ, Mayer K, Chesney M, Koblin B, Coates T. Use of non-occupational post-exposure prophylaxis does not lead to an increase in high risk sex behaviors in men who have sex with men participating in the EXPLORE trial. SIDA Behav. 2010;14(5) :1182-1189.

60. Sonder GJB, Prins JM, Regez RM, et al. Comparison of two HIV postexposure prophylaxis regimens among men who have sex with men in Amsterdam: adverse effects do not influence compliance. Sex Transm Dis. 2010;37(11) :681-686.

61. McAllister J, Read P, McNulty A, Tong WW, Ingersoll A, Carr A. Raltegravir-emtricitabine-tenofovir as HIV nonoccupational post-exposure prophylaxis in men who have sex with men: safety, tolerability and adherence. HIV Med. 2014;15(1) :13-22.

62. Jain S, Oldenburg CE, Mimiaga MJ, Mayer KH. Subsequent HIV infection among men who have sex with men who used non-occupational post-exposure prophylaxis at a Boston community health center: 1997-2013. AIDS Patient Care STDS. 2015;29(1) :20-25.

63. Foster R, McAllister J, Lisez TR, et al. Single-tablet emtricitabine-rilpivirine-tenofovir as HIV postexposure prophylaxis in men who have sex with men. Clin Infect Dis. 2015 : 1-5.

64. Linden JA, Oldeg P, Mehta SD, McCabe KK, LaBelle C. HIV postexposure prophylaxis in sexual assault: current practice and patient adherence to treatment recommendations in a large urban teaching hospital. Acad Emerg Med. 2005;12(7) :640-646.

65. Griffith WF, Ackerman GE, Zoellner CL, Sheffield JS. Sexual assault: a report on human immunodeficiency virus postexposure prophylaxis. Obstet Gynecol Int. 2010 (196963) : 1-6.

66. Olshen E, Hsu K, Woods ER, Harper M, Harnisch B, échantillons CL. Use of human immunodeficiency virus postexposure prophylaxis in adolescent sexual assault victims. Arc de Pediat Adolesc Med. 2006;160(7) :674-680.

67. Cardo D, Culver D, Ciesielski C, Srivastava P, Marcus R, Abiteboul D, et al. A Case–Control Study of HIV Seroconversion in Health Care Workers after Percutaneous Exposure. N Engl J Med. 20 nov. 1997 ; 337 : 1485-90.

68. Poynten IM, Smith DE, Cooper DA, Kaldor JM, Grulich AE. The public health impact of widespread availability of nonoccupational postexposure prophylaxis against HIV. Médecine VIH. 8 septembre 2007 ; (6) : 374-381.

69. Siegfried N, Muller M, Deeks JJ, Volmink J. Male circumcision for prevention of heterosexual acquisition of HIV in men. Base de données Cochrane Syst Rev. 2009 Apr 15; (2) : CD003362.

70. Millett GA, Flores SA, Marks G, Reed JB, Herbst JH. Circumcision status and risk of HIV and sexually transmitted infections among men who have sex with men: a meta-analysis. JAMA. 8 octobre 2008; 300 (14) : 1674-84. Erratum dans : JAMA. 18 mars 2009 ; 301 (11) : 1126-9.

71. Crepaz N, Marques G, Liau A, MM Mullins, Aupont LW, Marshall KJ et al. Prevalence of unprotected anal intercourse among HIV-diagnosed MSM in the United States: a meta-analysis. AIDS. 24 août 2009; 23 (13) : 1617-29.

72. Van De Ven P, Kippax S, Crawford J, Rawstorne P, Prestage G, Grulich A et al. In a minority of gay men, sexual risk practice indicates strategic positioning for perceived risk reduction rather than unbridled sex. Soins du SIDA. Août 2002; 14 (4) : 471-80.

73. Jin F, J Crawford, G Prestage, Zablotska I, J Imrie, Kippax S, et al. Unprotected anal intercourse, risk reduction behaviours, and subsequent HIV infection in a cohort of homosexual men. AIDS. 14 janvier 2009 ; 23 (2) : 243-52.

74. Baggaley RF, Boily M-C, White RG, Alary M. Risk of HIV-1 transmission for parenteral exposure and blood transfusion: a systematic review and meta-analysis. AIDS. 2006; 20 :805-12.

75. Vittinghoff E, Douglas J, F Judson, McKirnan D, MacQueen K, Buchbinder SP. Per-contact risk of human immunodeficiency virus transmission between male sexual partners. Am J Epidemiol.1999 1er août; 150 : 306-11.

76. Sexton J, Garnett G, Røttingen J-A. Metaanalysis and metaregression in interpreting study variability in the impact of sexually transmitted diseases on susceptibility to HIV infection. Sex Transm Dis. Juin 2005 ; 32 (6) : 351-7.

77. Campo J, Perea MA, del Romero J, Cano J, Hernando V, Bascones A. Oral transmission of HIV, reality or fiction? Une mise à jour. Oral Dis. Mai 2006 ; 12 (3) : 219-28.

78. Baggaley RF, White RG, Boily MC. Systematic review of orogenital HIV 1 transmission probabilities. Int J Epidemiol.2008 décembre; 37 (6) : 1255-65.

79. Morrow G, Vachot L, Vagenas P and Robbiani M. Current concepts of HIV transmission. Curr HIV/AIDS Rep. Fév 2007 ; 4 (1) : 29-35.).

80. Centers for Disease Control and Prevention. Oral sex and HIV Risk. CDC HIV / AIDS Facts, juin 2009.

81. del Romero J, B Marincovich, Castilla J, S Garcia, J Campo, Hernando V,. Evaluating the risk of HIV transmission through unprotected orogenital sex. SIDA 14 juin 2002 ; 16 (9) : 1296-97.

82. Raiteri R, Fora R, Sinicco A. No HIV-1 transmission through lesbian sex. Lancet 1994; 344 : 270.

83. European Study Group on Heterosexual Transmission of HIV. Comparison of female to male and male to female transmission of HIV in 563 stable couples. BMJ 28 mars 1992 ; 304 (6830) : 809-13.

84. Boily MC, Baggaley RF, Wang L, Masse B, White RG, Hayes RJ, et al. Heterosexual risk of HIV-1 infection per sexual act: systematic review and meta-analysis of observational studies. Lancet Infect Dis 9 fév. 2009 ; (2) : 118-29.

85. Rodger A, Cambiano V, Bruun T, Vernazza P, Collins S, Estrada V, et al. HIV transmission risk through condomless sex if the HIV positive partner is on suppressive ART: Étude PARTNER. Presentation at CROI, 2014 Mar 3-6;Boston, U.S..

86. Baggaley RF, White RG, Boily M-C. HIV transmission risk through anal intercourse: systematic review, meta-analysis and implications for HIV prevention. Int J Epidemiol. Août 2010; 39 (4) : 1048-63.

87. Halperin DT, Shiboski SC, Palefsky JM et Padian NS. High level of HIV-1 infection from anal intercourse: a neglected risk factor in heterosexual AIDS prevention. Document présenté à la XIVe Conférence internationale sur le sida. 2002; Barcelone, Espagne.

88. Leynaert B, Downs AM, de Vincenzi I. Heterosexual transmission of human immunodeficiency virus: variability of infectivity throughout the course of infection. European Study Group on Heterosexual Transmission of HIV. Am J Epidemiol. 1er juillet 1998 ; 148 (1) : 88-96

89. Jin F, Jansson J, Loi M, Prestage GP, Zablotska I, Imrie JC, et al. Per-contact probability of HIV transmission in homosexual men in Sydney in the era of HAART. AIDS. 27 mars 2010 ; 24 (6) : 907-13.

90. DeGruttola V, Seage GR 3rd, Mayer KH and Horsburgh CR Jr. Infectiousness of HIV between male homosexual partners. J Clin Epidemiol.1989; 42 (9) : 849-56.

91. Jacquez JA, Koopman JS, Simon CP and Longini IM Jr. Role of the primary infection in epidemics of HIV infection in gay cohorts. J Acquir Immune Defic Syndr. Novembre 1994 ; 7 (11) : 1169-84.

92. Bavinton B, B. Grinsztejn, N. Phanuphak, F. Jin, I. Zablotska, G. Prestage, et al. HIV treatment prevents HIV transmission in male serodiscordant couples in Australia, Thailand and Brazil. 9th International AIDS Society Conference on HIV Science, Paris, abstract no TUAC0506LB, July 2017.

93. Berthier A, R Fauchet, N Genetet, J Fonlupt, Genetet N, Gueguen M, et al. Transmissibility of human immunodeficiency virus in haemophilic an non-haemophilic children living in a private school in France. Lancet 13 sep. 1986 ; 2 (8507) : 598-601.

94. Fischl MA, Dickinson GM, Scott GE, et al. Evaluation of heterosexual partners, children and household contacts of adults with AIDS. JAMA 1987; 257 : 640-4.

95. Friedland G, Kahl P, Saltzman B, Rogers M, Feiner C, Mayers M, et al. Additional evidence for lack of transmission of HIV infection by close interpersonal (casual) contact. AIDS. 1er juil. 1990; 4 (7) : 639-44.

96. Rogers MF, White CR, R Sanders, C Schable, TE Ksell, Wasserman RL, et al. Lack of transmission of human immunodeficiency virus from infected children to their household contacts. Pédiatrie. Fév 1990 ; 85 (2) : 210-14.).

97. Courville TM, Caldwell B, Brunell P. Lack of Evidence of Transmission of HIV-1 to Family Contacts of H1V-1 Infected Children. Pédiatrie clinique. Mars 1998 ;37(3) :175-8.

98. Lusher JM, Operskalski EA, Lee H, Mosley JW, Aledort LM, Dietrich SL, et al. Risk of human immunodeficiency virus type 1 infectioninfection among sexual and nonsexual household contacts of persons with congenital clotting disorders. Pédiatrie. Août 1991; 88 (2) : 242-9.

99. Gilbart VL. Unusual HIV transmissions through blood contact: analysis of cases reported in the United Kingdom to December 1997. Communicable Disease and Public Health. Juin 1998 ; 1 (2) : 108-13.

100. Yeung SC, F Kazazi, Randle CG, et al. Patients infected with human immunodeficiency virus type 1 have low levels of virus in saliva even in the presence of periodontal disease. J Infect Dis. Avril 1993 ; 167 (4) : 803-9.

101. Shine N, Konopka K , Düzgüneş N : The anti-HIV-1 activity associated with saliva. J Dent Res. Fév 1997 ; 76 (2) : 634-640.).

102. Shugars DC, Schock DC, Patton J. HIV-1 RNA load in blood plasma, saliva and crevicular fluid. J Dent Res. 1998; 77 (numéro spécial A) : 285.

103. Shugars DC, Wahl S. The role of the oral environment in HIV-1 transmission. JADA. Juillet 1998 ; 129 (7) : 851-8.

104. Malamud D, Friedman HM. HIV in the oral cavity: virus, viral inhibitory activity, and antiviral antibodies. Crit Rev Oral Biol Med. 1993; 4 (3-4) : 461-6.

105. Archibald DW, Cole GA. In vitro inhibition of HIV-1 infectivity by human salivas. AIDS Res Hum Retroviruses, déc. 1990; 6 (12) : 1425-32.

106. Yeh CK, Handelman B, Fox PC, Baum BJ. Further studies of salivary inhibition of HIV-1 infectivity. J Acquir Immune Defic Syndr. 1992;5(9) :898-903.

107. Cresswell FV, Ellis J, Hartley J, CA Sabin, Orkin C, Churchill DR. A systematic review of risk of HIV transmission through biting or spitting: implications for policy. Médecine VIH. 23 avril 2018. Disponible au https://onlinelibrary.wiley.com/doi/abs/10.1111/hiv.12625

108. Tsoukas C et al. Lack of transmission of HIV through human bites and scratches. JAIDS 1988; 1 (5) : 505-7.

109. Richman KM, Richman LS. The potential for transmission of human immunodeficiency virus through human bites. J Acquir Immune Defic Syndr. 1993; 64 :40-46.

110. Shirley LR, Ross SA, Risk of transmission of human immunodeficiency virus by bite of an infected toddler. Le Journal de Pédiatrie. Mars 1989 ; 114 (3) : 425-7.

111. Drummond R. Seronegative 18 months after being bitten by a patient with AIDS. JAMA. 7 novembre 1986 ; 256 (17) : 2342-3.

112. Romea S, Alkiza ME, Ramon JM, Oromí J. Risk for occupational transmission of HIV infection among health care workers. European Journal of Epidemiology. Avril 1995 ; 11 (2) : 225-9.

113. Verrusio AC, Risk of transmission of the human immunodeficiency virus to health care workers exposed to HIV-infected patients: a review, JADS. Mars 1989 ; 118 (3) : 339-42.

114. Henderson DK, Fahey BJ, M Willy, Schmitt JM, Carey K, Koziol DE, et al. Risk for Occupational Transmission of Human Immunodeficiency Virus Type 1 (HIV-1) Associated with Clinical Exposures: A Prospective Evaluation. Ann Intern Med. 15 novembre 1990 ; 113 (10) : 740-746.

115. Tereskerz PM, Bentley M, Jagger J. Risk of HIV-1 infection after human bites. Lancet. 30 nov; 1996 ; 348 (9040) : 1512.

116. Sabin CA, Lundgren JD. The natural history of HIV infection. Current Opinion in HIV and AIDS. 2013;8(4) :311-317.

117. Okulicz JF, Marconi VC, Landrum ML, Wegner S, Weintrob A, Ganesan A, et al. Clinical Outcomes of Elite Controllers, Viremic Controllers, and Long-Term Nonprogressors in the US Department of Defense HIV Natural History Study. J Infect Dis. 1er décembre 2009; 200 (11) : 1714-23.

118. McManus H, O'Connor CC, Boyd M, Broom J, Russell D, Watson K, et al. Long-term survival in HIV positive patients with up to 15 Years of antiretroviral therapy. PLoS One. 2012 7 novembre; 7 (11).

119. Lee FJ, Amin J, Carr A. Efficacy of initial antiretroviral therapy for HIV-1 infection in adults: a systematic review and meta-analysis of 114 studies with up to 144 weeks' follow-up. PLoS One. 15 mai 2014 ; 9 (5) : e97482.

120. Groupe d'étude INSIGHT START. Initiation of Antiretroviral Therapy in Early Asymptomatic HIV Infection. N Engl J Med. 27 août 2015 ; 373 (9) : 795-807.

121. Stephan C, Hill A, Sawyer W, van Delft Y, Moecklinghoff C. Impact of baseline HIV-1 RNA levels on initial highly active antiretroviral therapy outcome: a meta-analysis of 12,370 patients in 21 clinical trials. HIV Med. Mai 2013 ; 14 (5) : 284-92.

122. Trickey A, May M, JJ Vehreschild, Obel N, Gill MJ, Crane H, et al. Survival of HIV-positive patients starting antiretroviral therapy between 1996 and 2013: a collaborative analysis of cohort studies. The Lancet HIV. 4 août 2017 ; (8) : e349-56.

123. Patterson S, Céscon A, Samji H, Chan K, Zhang W, Raboud J, et al. Life expectancy of HIV-positive individuals on combination antiretroviral therapy in Canada. BMC Infect Dis. 2015 juil 17; 15 : 274.

124. Zhu H, S Napravnik, JJ Eron, Cole SR, Ma Y, Wohl DA, et al. Decreasing excess mortality of HIV-infected patients initiating antiretroviral therapy: comparison with mortality in general population in China, 2003-2009. J Acquir Immune Defic Syndr. 15 août 2013 ; 63 (5) : e150-7.

125. Teeraananchai S, Kerr SJ, J Amin, Ruxrungtham K, Law MG. Life expectancy of HIV-positive people after starting combination antiretroviral therapy: a meta-analysis. HIV Med. Avril 2017 ; 18 (4) : 256-66.

126. Lohse N, Obel N. Update of Survival for Persons with HIV Infection in Denmark. Ann Intern Med. 15 novembre 2016 ; 165 (10) : 749-750.

127. Prix AJ, Glynn J, Chihana M, N Kayuni, Floyd S, Slaymaker E, et al. Sustained 10-year gain in adult life expectancy following antiretroviral therapy roll-out in rural Malawi: Juillet 2005 à juin 2014. Int J Epidemiol. 2017;46(2) :479-91.

128. Nsanzimana S, Remera E, S Kanters, Chan K, Ford Forrest, Ford N, et al. Life expectancy among HIV-positive patients in Rwanda: a retrospective observational cohort study. Lancet Glob Health. 2015 mars; 3 (3) : e169-77.

129. Johnson LF, J Mossong, RE Dorrington, Schomaker M, CJ Hoffmann, Keizer O, et al. International Epidemiologic Databases to Evaluate AIDS Southern Africa Collaboration. Life expectancies of South African adults starting antiretroviral treatment: collaborative analysis of cohort studies. PLoS Med. 9 avril 2013 ; 10 (4) : e1001418.

130. Reniers G, Blom S, Calvert C, Martin-Onraet A, AJ Herbst, Eaton JW, et al. Trends in the burden of HIV mortality after roll-out of antiretroviral therapy in KwaZulu-Natal, South Africa: an observational community cohort study. Lancet VIH. Mars 2017 ; 4 (3) : e113-e121.

131. Gueler A, Moser A, Calmy A, Günthard HF, Bernasconi E, Furrer H, et al. Life expectancy in HIV-positive persons in Switzerland: matched comparison with general population. AIDS. 28 janvier 2017 ; 31 (3) : 427-36.

132. Teeraananchai S, Chaivooth S, Kerr SJ, Bhakeecheep S, Avihingsanon A, Teeraratkul A, Sirinirund P, Law MG, Ruxrungtham K. Life expectancy after initiation of combination antiretroviral therapy in Thailand. Antivir Ther. 5 janv 2017.

133. Asiki G, Reniers G, Newton R, Baisley K, Nakiyingi-Miiro J, Slaymaker E, et al. Adult life expectancy trends in the era of antiretroviral treatment in rural Uganda (1991-2012). AIDS. 28 janvier 2016 ; 30 (3) : 487-93.

134. Mai MT, Gompels M, Delpech V, Porter K, Orkin C, Kegg S, et al. Impact on life expectancy of HIV-1 positive individuals of CD4+ cell count and viral load response to antiretroviral therapy. AIDS. 15 mai 2014 ; 28 (8) : 1193-202.

135. Samji H, Céscon A, Hogg RS, Modur SP, Althoff KN, Buchacz K, et al. Closing the gap: increases in life expectancy among treated HIV-positive individuals in the United States and Canada. PLoS One. 18 décembre 2013 ; 8 (12) : e81355.

136. Siddiqi AE, Hall HI, Hu X, Song R. Population-Based Estimates of Life Expectancy After HIV Diagnosis: États-Unis 2008 - 2011. J Acquir Immune Defic Syndr. 1er juin 2016 ; 72 (2) : 230-6.

137. Marcus JL, CR Chao, Leyden WA, Xu L, Quesenberry CP Jr, Klein DB, et al. Narrowing the Gap in Life Expectancy Between HIV-Infected and HIV-Uninfected Individuals With Access to Care. J Acquir Immune Defic Syndr. 1er sept. 2016 ; 73 (1) : 39-46.

138. Furuya-Kanamori L, Kelly MD, McKenzie SJ. Co-morbidity, ageing and predicted mortality in antiretroviral treated Australian men: a quantitative analysis. PLoS One. 25 octobre 2013; 8 (10) : e78403.

139. van Sighem AI, Gras LA, Reiss P, Brinkman K, de Wolf F. Life expectancy of recently diagnosed asymptomatic HIV-infected patients approaches that of uninfected individuals. AIDS. 19 Juin 2010 ; 24 (10) : 1527-35.

140. Nglazi M, West S, Dave J, Levitt N, Lambert E. Quality of life in individuals living with HIV/AIDS attending a public sector antiretroviral service in Cape Town, South Africa. BMC Santé publique. 3 Juillet 2014 ; 14 : 676.

141. Wandeler G, Johnson LF, Egger M. Trends in life expectancy of HIV-positive adults on antiretroviral therapy across the globe: comparisons with general population. Curr Opin HIV SIDA. Septembre 2016; 11 (5) : 492-500.

142. Palella FJ Jr, Baker RK, Moorman AC, Chmiel JS, Wood KC, Brooks JT, et al. Mortality in the highly active antiretroviral therapy era: changing causes of death and disease in the HIV outpatient study. J Acquir Immune Defic Syndr. Septembre 2006; 43 (1) : 27-34.

143. Crum NF, RH Riffenburgh, S Wegner, Agan BK, Tasker SA, Spooner KM, et al. Comparisons of causes of death and mortality rates among HIV-infected persons: analysis of the pre-, early, and late HAART (highly active antiretroviral therapy) eras. J Acquir Immune Defic Syndr. 1er février 2006 ; 41 (2) : 194-200.

144. Deeks SG, Phillips AN. HIV infection, antiretroviral treatment, ageing, and non-AIDS related morbidity. BMJ. 31 janvier 2009 ; 338 : 288-92.

145. Kojic EM, Wang CC, Cu-Uvin S. (2007). HIV and menopause: a review. J Womens Health (Larchmt). Dec. 16 (10) : 1402-11.

146. Manfredi R. HIV disease and advanced age: an increasing therapeutic challenge. Drugs Aging. 2002;19(9) :647-69.

147. Manfredi R. HIV infection and advanced age emerging epidemiological, clinical, and management issues. Ageing Res Rev. 2004;Jan;3(1):31-54.

148. Serrano-Villar S, F Gutiérrez, Miralles C, J Berenguer, Rivero A, Martínez E, et al. Human Immunodeficiency Virus as a Chronic Disease: Evaluation and Management of Nonacquired Immune Deficiency Syndrome-Defining Conditions. Open Forum Infect Dis. 12 mai 2016 ; 3 (2) : ofw097.

149. Narayan KM, Miotti PG, PN Anand, Kline LM, Harmston C, R Gulakowski 3e, et al. HIV and noncommunicable disease comorbidities in the era of antiretroviral therapy: a vital agenda for research in low- and middle-income country settings. J Acquir Immune Defic Syndr. 1er septembre 2014 ; 67 Suppl 1 : S2-7.

150. Bloomfield GS, P Khazanie, Morris A, C Rabadán-Diehl, Benjamin LA, Murdoch D, et al. HIV and noncommunicable cardiovascular and pulmonary diseases in low- and middle-income countries in the ART era: what we know and best directions for future research. J Acquir Immune Defic Syndr. 1er septembre 2014 ; 67 Suppl 1 : S40-53.

151. Helleberg M, Afzal S, Kronborg G, Larsen CS, Pedersen G, Pedersen C, et al.Mortality Attributable to Smoking Among HIV-1-Infected Individuals: A Nationwide, Population-Based Cohort Study. Clin Infect Dis. Mars 2013 ;56(5) :727-34.

152. Rodriguez B, Sethi AK, Cheruvu VK, Mackay W, Bosch RJ, Kitahata M, et al. Predictive value of plasma HIV RNA level on rate of CD4 T-cell decline in untreated HIV infection. Journal de l'American Medical Association, 27 septembre 2006, 296 (12) : 1498-1506.

153. Bernard E, Azad Y, Delpech V, Geretti AM. VIH Forensics II : Estimating the likelihood of recent HIV infection: Implications for criminal prosecution. National AIDS Trust. Londres. Juillet 2011.

154. Goodenow M, Huet T, Saurin W, Kwok S, Sninsky J, Wain-Hobson S. HIV-1 isolates are rapidly evolving quasispecies: evidence for viral mixtures and preferred nucleotide substitutions. J Acquir Immune Defic Syndr 1989; 2 : 344-352.

155. Abecasis A, Pingarilho M, Vandamme A-M. Phylogenetic analysis as a forensic tool in HIV transmission investigations: a literature review. AIDS 2017 26-31 déc. 2017.

156. Romero-Severson E, Skar H, Bulla I, Albert J, Leitner T. Timing and order of transmission events is not directly reflected in a pathogen phylogeny. Mol Biol Evol 2014; 31 : 2472-2482.

157. Rambaut A, Posada D, Crandall KA, Holmes CE. The causes and consequences of HIV evolution. Nat Rev Genet 2004; 5 : 52-61.

158. Bernard EJ, Azad Y, AM Vandamme, Weait M, Geretti AM. HIV forensics: pitfalls and acceptable standards in the use of phylogenetic analysis as evidence in criminal investigations of HIV transmission. HIV Med. Septembre 2007 ; 8 (6) : 382-387.

159. Romero-Severson EO, Bulla I, Leitner T. Phylogenetically resolving epidemiologic linkage. Proc Natl Acad Sci USA. 8 mars 2016 ; 113 (10) : 2690-5.

160. Huelsenbeck J, Hillis DM. Success of phylogenetic methods in the four-taxon case. Syst Biol 1993; 247-264.

161. Wertheim JO, Sanderson MJ, Worobey M, Bjork A. Relaxed molecular clocks, the bias-variance trade-off, and the quality of phylogenetic inference. Syst Biol 2010; 59 : 1-8.

162. González-Candelas F, Bracho MA, Wróbel B, Moya A. Molecular evolution in court: analysis of a large hepatitis C virus outbreak from an evolving source. BMC Biol 2013; 11h76.

163. Abecasis AB, Geretti AM, Albert J, Power L, Weait M, Vandamme A-M. Science in court: the myth of HIV fingerprinting. Lancet Infect Dis. Fév. 2011 11 (2) : 78-9.).

164. Ou CY, CA Ciesielski, G Myers, CI Bandea, Luo CC, Korber BT, et al. Molecular epidemiology of HIV transmission in a dental practice. Science 1992; 256 : 1165-1171.

165. Smith TF, Waterman MS. The continuing case of the Florida dentist. Science 1992; 256 : 1155-1156.

166. DeBry RW, Abele LG, Weiss SH, Hill MD, Bouzas M, Lorenzo E, et al. Dental HIV transmission? Nature 1993; 361 : 691.

167. Lemey P, S Van Dooren, Van Laethem K, Schrooten Y, Derdelinckx I, Goubau P, et al. Molecular testing of multiple HIV-1 transmissions in a criminal case. AIDS Lond Engl 2005; 19 : 1649-1658.

168. Goedhals D, Rossouw I, Hallbauer U, Mamabolo M, de Oliveira T. The tainted milk of human kindness. Lancet Lond Engl 2012; 380 : 702.

169. Paraschiv S, Banica L, Nicolae I, Niculescu I, Abagiu A, Jipa R, et al. Epidemic dispersion of HIV and HCV in a population of co-infected Romanian injecting drug users. PloS One 2017; 12.
