# Supplementary material for: Expert consensus statement on the science of HIV in the context of criminal law
Source: J Int AIDS Soc. 2018 Jul 25;21(7):e25161. doi: 10.1002/jia2.25161 (PMC6058263; doi:10.1002/jia2.25161)
Supplement: Supplementary file 4 — Supplementary Material S4. Expert Consensus Statement RUSSIAN Translation. [file JIA2-21-e25161-s004.pdf]

Заявление об экспертном консенсусе в отношении научных данных о ВИЧ- инфекции в контексте уголовного права

Франсуаза Барре-Синуси[1], Салим С. Абдул Карим[2, 3, 4], Ян Альберт[5], Линда-Гейл Беккер[6], Крис Бейер[7], Педро Канн[8, 9, 10], Александра Кальми[11], Беатрис Гринцтейн[12], Эндрю Грулич[13], Адебе Камарулзаман[14], Нагалингесваран Кумарасамы[15], Мона Рафик Лаутфи[16, 17, 18], Камал Марум Эль Филали[19], Сулейман Мбуп[20], Хулио С.Г. Монтанер[21, 22], Паула Мундери[23], Вадим Покровский[24, 25], Энн-Мике Вандамм[26, 27], Бенджамин Янг[28], Питер Годфри-Фауссет[§29, 30]

1. Институт Пастера, Париж, Франция
2. Mailman School of Public Health, Колумбийский университет, Нью-Йорк, США
3. Centre for the AIDS Program of Research in South Africa, Университет Квазулу-Натал, Дурбан, ЮАР
4. Weill Medical College, Корнеллский университет, Нью-Йорк, США
5. Department of Microbiology, Tumor and Cell Biology, Каролинский институт, Стокгольм, Швеция
6. Institute of Infectious Disease and Molecular Medicine, Кейптаунский университет, Кейптаун, ЮАР
7. Department of Epidemiology, Center for AIDS Research and Center for Public Health and Human Rights, John Hopkins Bloomberg School of Public Health, Мэриленд, США
8. Infectious Diseases Unit, Juan A. Fernandez Hospital, Буэнос-Айрес, Аргентина
9. Buenos Aires University Medical School, Буэнос-Айрес, Аргентина
10. Fundación Huésped, Буэнос-Айрес, Аргентина
11. Infectious Diseases, Geneva University Hospital, Женева, Швейцария
12. Instituto Nacional de Infectologia Evandro Chagas-Fiocruz, Fiocruz, Рио-де-Жанейро, Бразилия
13. Kirby Institute, Университет Нового Южного Уэльса, Сидней, Австралия
14. Faculty of Medicine, Университет Малайя, Куала-Лумпур, Малайзия
15. YRGCARE Medical Centre, Voluntary Health Services, Ченнай, Индия
16. Women's College Research Institute, Торонто, Канада
17. Women’s College Hospital, Торонто, Канада
18. Department of Medicine, Университет Торонто, Канада
19. Infectious Diseases Unit, Ibn Rochd Universtiy Hospital, Касабланка, Марокко
20. Institut de Recherche en Santé, de Surveillance Epidemiologique et de Formations, Дакар, Сенегал
21. Faculty of Medicine, Университет Британской Колумбии, Ванкувер, Канада
22. BC Centre for Excellence in HIV/AIDS, Ванкувер, Канада
23. Международная ассоциация поставщиков услуг по уходу в сфере СПИДа, Кампала, Уганда
24. Российский университет дружбы народов (Университет РУДН), Москва, Российская Федерация
25. Центральный НИИ эпидемиологии, Федеральная служба по надзору в сфере защиты прав потребителей и благополучия человека, Москва, Российская Федерация
26. KU Leuven, Department of Microbiology and Immunology, Rega Institute for Medical Research, Clinical and Epidemiological Virology, Лёвен, Бельгия
27. Center for Global Health and Tropical Medicine, Unidade de Microbiologia, Instituto de Higiene e Medicina Tropical, Universidade Nova de Lisboa, Лиссабон, Португалия

28. Международная ассоциация поставщиков услуг по уходу в сфере СПИДа, Вашингтон, округ Колумбия, США
29. ЮНЭЙДС, Женева, Швейцария
30. Department of Infectious and Tropical Diseases, Лондонская школа гигиены и тропической медицины, Лондон, Англия

[§] **Автор для корреспонденции:** Проф. Питер Годфри-Фауссет

Адрес: ЮНЭЙДС, Avenue Appia 20, 1211 Женева, Швейцария

Телефон: + 41 22 791 4054

Эл. почта: godfreyp@unaids.org

Адреса электронной почты авторов:

FB-S: francoise.barre-sinoussi@pasteur.fr

JA: Jan.Albert@ki.se

SSAK: Salim.AbdoolKarim@caprisa.org

L-GB: linda-gail.bekker@hiv-research.org.za

CB: cbeyrer@jhu.edu

PC: pedro.cahn@huesped.org.ar

AC: alexandra.calmy@hcuge.ch

PG-F: faussettp@unaids.org

BG: beatriz.grinsztejn@gmail.com

AG: agrulich@kirby.unsw.edu.au

AK: adeeba@ummc.edu.my

NM: kumarasamy@yrgcare.org

MRL: mona.loutfy@wchospital.ca

KMEF: mefkamal@hotmail.com

SM: souleymane.mboup@iressef.org

JSGM: jmontaner@cfenet.ubc.ca

PM: munderip@gmail.com

VP: pokrovsky.vad@yandex.ru

A-MV: annemie.vandamme@kuleuven.be

BY: byoung@iapac.org

Ключевые слова:
- права человека
- закон и политика
- факторы риска
- политика
- криминализация
- криминализация
- уголовное право
- судебное преследование

Аннотация

Введение

В глобальном масштабе судебные преследования за сокрытие информации о наличии ВИЧ, поставление другого лица в опасность передачи ВИЧ или передача ВИЧ часто связаны с сексуальной активностью, кусанием или плеванием. Это включает и те случаи, когда не было причинено никакого вреда, передача ВИЧ не происходила, и она была крайне маловероятной или невозможной. Таким образом, судебные преследования не всегда руководствуются наилучшими имеющимися научными и медицинскими доказательствами.

Обсуждение

Двадцать ученых из разных регионов мира разработали это Заявление об экспертном консенсусе в отношении использования научных данных о ВИЧ в системе уголовного правосудия. Был проведен и описан подробный анализ наилучших имеющихся данных научных и медицинских исследований о передаче ВИЧ, эффективности лечения и судебно-филогенетических доказательств, позволяющий лучше понять их в уголовно-правовом контексте.

Описание возможности передачи ВИЧ ограничивалось действиями, наиболее часто рассматриваемыми в уголовных делах. Возможность передачи ВИЧ во время одного, конкретного акта была расположена по убыванию вероятного риска (с указанием на то, что возможность передачи ВИЧ варьируется в зависимости от целого ряда пересекающихся факторов, включая вирусную нагрузку, использование презерватива и другие методы снижения риска). Текущие доказательства свидетельствуют, что возможность передачи ВИЧ в течение одного эпизода секса, кусания или плевания находится в диапазоне от невозможности до низкой вероятности.

Дальнейшие исследования рассматривали положительное влияние на здоровье современных антиретровирусных препаратов, которые повысили ожидаемую продолжительность жизни большинства людей, живущих с ВИЧ, до значения, характерного для ВИЧ-отрицательных людей, превратив ВИЧ-инфекцию в хроническое управляемое состояние здоровья. Наконец, рассмотрение использования научных доказательств в суде показало, что сам по себе филогенетический анализ не может преодолеть обоснованное сомнение в том, что один человек заразил другого, хотя его можно использовать для оправдания обвиняемого.

Заключение

Применение современных научных доказательств в уголовных делах может ограничить несправедливые судебные преследования и осуждения. При применении закона в делах, связанных с ВИЧ, авторы рекомендуют проявлять осторожность при возбуждении судебного преследования и рекомендуют представителям власти, сотрудникам правоохранительных и судебных систем, использовать современные научные знания на основе значительных достижений в области науки о ВИЧ последних тридцати лет .

Введение

По меньшей мере 68 стран имеют законы, которые специально предусматривают уголовную ответственность за сокрытие информации о наличие ВИЧ-инфекции от своего партнера по сексу, поставление другого лица в опасность передачи ВИЧ или передачу ВИЧ. Известно, что тридцать три страны применяли другие уголовно-правовые положения в подобных случаях (Неопубликованные данные, HIV Justice Network, 2018 г.). Большинство судебных преследований по поводу предполагаемого риска заражения ВИЧ, связаны с сексуальной активностью, но также были судебные преследования за такие действия, как кусание и плевание (Неопубликованные данные, HIV Justice Network, 2018 г.). Эти законы и судебные преследования не всегда исходили и продолжают исходить из наилучших имеющихся научных и медицинских доказательств [1], не отражают прогресса в области знаний о ВИЧ-инфекции и ее лечении, на них могут оказывать влияние социальная стигма и страх в связи с ВИЧ [2]. ВИЧ по-прежнему остается выделенным в отдельную категорию, при этом судебные преследования происходят в случаях, когда не было причинено никакого вреда; когда передача ВИЧ не происходила, была невозможной или крайне маловероятной; и когда передача не была ни вменяемой в вину, ни доказанной [1,3].

В этом контексте двадцать ученых в области ВИЧ с опытом в сфере научных исследований, эпидемиологии и ухода за пациентами из регионов по всему миру, разработали это Заявление о консенсусе. Это было вызвано опасениями, что уголовное право иногда применяется в противоречии с современными медицинскими и научными доказательствами: в том числе, завышая как риск передачи ВИЧ, так и потенциальный вред здоровью и благополучию человека. Такое ограниченное понимание текущей науки о ВИЧ способствует усилению стигмы и может привести к судебным ошибкам. Это также может подрывать усилия по борьбе с эпидемией ВИЧ [4]. Заявление о консенсусе было одобрено и другими учеными со всего мира (см. Дополнительный материал Д1), а также Международным обществом борьбы со СПИДом, Международной ассоциацией поставщиков услуг по уходу в сфере СПИДа и Объединенной программой Организации Объединенных Наций по ВИЧ/СПИДу (Резюме этого Заявления включено в Дополнительный материал Д2).

Это Заявление о консенсусе направлено на оказание помощи научным экспертам, рассматривающим отдельные уголовные дела, в которых предполагается сокрытие ВИЧ, (предполагаемое или возможное) поставление другого лица в опасность передачи ВИЧ или передача ВИЧ. В нем представлено экспертное заключение относительно индивидуальной динамики передачи ВИЧ (т. е. «возможности»

передачи), долгосрочного воздействия хронической ВИЧ-инфекции (т. е. «вреда» ВИЧ) и применения филогенетического анализа в качестве доказательства. В нем описывается возможность передачи ВИЧ между людьми, которые участвуют в конкретном действии в определенное время при определенных обстоятельствах, поскольку это, как правило, является предметом уголовных дел, и сообщаются существующие научные доказательства, касающиеся ВИЧ, таким образом, чтобы это было понятно людям, не являющимся учеными. Заявление о консенсусе было переведено с английского языка на французский, русский и испанский языки (см. Дополнительный материал Д3, Д4, Д5)

Обсуждение

Первая часть этого Заявления фокусируется на возможности передачи ВИЧ во время конкретных действий, которые обычно рассматриваются в судебных преследованиях: сексуальная активность, кусание или плевание [3]. В нем не рассматриваются другие способы возможной передачи ВИЧ, например через переливание крови, травму в результате укола иглы, употребление инъекционныех наркотиков или грудное вскармливание.

В ходе первой встречи в Сиэтле (в феврале 2017 года) было принято решение о содержании и структуре этого Заявления о консенсусе. Подробный обзор литературы был подготовлен на основе поиска литературы, опубликованной на английском языке, с использованием онлайн-базы данных PubMed до апреля 2017 года. Были использованы конкретные поисковые термины, касающиеся возможности передачи ВИЧ, в том числе «ВИЧ и вирусная нагрузка», «риск передачи ВИЧ половым путем во время одного акта», «передача ВИЧ при оральном сексе», «передача ВИЧ при анальном сексе», «передача ВИЧ при вагинальном сексе с презервативом во время одного акта», «передача ВИЧ при анальном сексе с презервативом во время одного акта» и «передача ВИЧ при анальном сексе при обрезании во время одного акта». Ключевые статьи были использованы для поиска связанных статей. Предпочтение было отдано метаанализу, обзорам и важным исследованиям. Другие источники были идентифицированы авторами-экспертами. При необходимости использовались тезисы научных конференций.

Затем авторы участвовали в нескольких раундах написания и переработки документа и, учитывая наилучшие имеющиеся данные научных и медицинских исследований в соответствии со следующей иерархией: систематический обзор рандомизированных

клинических испытаний; рандомизированные клинические исследования; и сравнительные исследования (т. е. когортные исследования, исследования случай-контроль и исследования исторического контроля). Были проведены две телеконференции для обсуждения предварительного проекта документа, за которым последовали три раунда его переработки в ходе электронной переписки между всеми авторами. Были проведены консультации с национальными и международными экспертами по правовым вопросам, в том числе сотрудниками ЮНЭЙДС, по применению уголовного права в делах, связанных с ВИЧ. Второе очное совещание было созвано в Париже (в июле 2017 года) для решения неурегулированных вопросов, связанных с анализом данных. Авторы провели дальнейшие раунды комментирования и переработки документа, чтобы прийти к согласию о том, что Заявление о консенсусе точно передает текущие научные исследования, связанные с передачей, вредом ВИЧ и использованием научных доказательств в суде.

Авторы рассмотрели численные результаты и статистические оценки из всех упомянутых здесь исследований, включая сводные данные из отчетов, представленных в систематической или табличной форме (например, работ Patel et al. [5]). Доказательства, устанавливающие оценки возможности передачи ВИЧ через различные действия, отличаются как по типу, так и по качеству; авторы учли эти соображения в своей оценке возможности в связи с различными действиями. Авторы считают, что доказательства передачи через различные действия делятся на три категории (таблица 1).

Таблица 1. Качественная шкала доказательств возможности передачи ВИЧ

Действия, для которых возможность передачи может быть оценена с некоторой степенью уверенности, поскольку были проведены множественные когортные исследования (например, такие действия, как вагинальный или анальный секс).
Действия, для которых возможность передачи может быть оценена с меньшей степенью достоверности из отдельных описанных случаев, биологической вероятности или математических моделей (например, такие действия, как оральный секс или передача через предсеменную жидкость).
Действия, для которых осуществление передачи биологически неправдоподобно, поскольку условия, необходимые для передачи, отсутствуют (например, плевание).

При описании доказательств авторы стремились использовать научные концепции способами, полезные в контексте уголовного права. Например, статистическая концепция доверительных интервалов предназначена для устранения

неопределенности, присущей результатам, полученным из выборки подмножества населения. Когда дело касается вероятностей, которые равны или приближаются к нулю, доверительные интервалы приобретают особое значение, поскольку тот факт, что что-то не наблюдалось во время исследования, не может доказать, что этого никогда не произойдет. Чем больше исследование, тем точнее авторы могут оценить, что вероятность равна нулю. Соответственно, нулевая вероятность, вычисленная по данным исследования, ассоциируется с доверительным интервалом от нуля до небольшой положительной вероятности. Важно, чтобы расчеты доверительных интервалов не были неверно истолкованы, чтобы не преувеличить отдаленные теоретические возможности.

Рассмотрение методологии и результатов исследований, упомянутых в этом Заявлении о консенсусе, привело к разработке трех расположенных по убыванию степени риска описательных термина , характеризующих возможности передачи ВИЧ во время одного, конкретного акта (таблица 2).

Таблица 2. Определение возможности передачи ВИЧ во время одного, конкретного акта

**Терминология этого заявления**	**Возможность передачи во время одного акта**
Низкая возможность	Передача во время одного акта возможна, но вероятность низкая.
Незначительная возможность	Передача во время одного акта является крайне маловероятной, редкой или удаленной.
Невозможность	Возможность передачи во время одного акта либо биологически неправдоподобна, либо фактически равна нулю.

Важно отметить, что это Заявление о консенсусе не предназначено для использования в качестве документа общественного здравоохранения, для информирования о профилактике, лечении и уходе в связи с ВИЧ, либо для целей планирования или подготовки программ действий в связи профилактикой, лечением и уходом при ВИЧ. Его подход, основанный на риске на индивидуальном уровне, который может быть применен в условиях уголовного правосудия, отличается от описаний рисков на уровне населения, которые используются в контексте общественного здравоохранения и часто описывают половые акты в диапазоне от «низкого» до «высокого» риска. Различия между описательными терминами общественного здравоохранения и описательными терминами, используемыми в этом Заявление о консенсусе, отражают как историю вопроса, так и контекст. Во-первых, определения общественного здравоохранения, используемые для описания риска передачи ВИЧ, были разработаны в начале

эпидемии ВИЧ, до появления последних доказательств о передаче ВИЧ. Во-вторых, они описывают относительный риск (а не абсолютный риск) как средство, помогающее людям уменьшить возможность передачи ВИЧ путем сравнения различных действий.

Хотя простота такой терминологии общественного здравоохранения изначально была предназначена для поддержки эффективных широкомасштабных просветительских кампаний в области общественного здравоохранения для профилактики ВИЧ, ее обобщенные категории в настоящее время создают реальные проблемы для тех, кто разрабатывает текущие сообщения о ВИЧ в рамках оздоровительных кампаний на основе современных научных доказательств [6], включая доказательства со стороны различных переменных, которые изменяют риск, связанный с конкретными действиями, таких как вирусная нагрузка. В некоторых случаях понимание рискованности некоторых половых актов, сообщаемое характеристиками общественного здравоохранения, также было неправильно применено в контексте уголовного судопроизводства, например канадского дела Мабиор [7,8]. Соответственно, хотя передача половым путем является распространенной формой передачи ВИЧ на уровне населения всего мира, это Заявление о консенсусе признает, что вероятность передачи ВИЧ во время одного полового контакта варьирует от невозможности до низкой возможности; при этом она варьирует от невозможности до незначительной возможности в случае плевания или кусания. Такой подход к науке о ВИЧ в контексте уголовного права аналогичен тому, который использовался в национальных научных заявлениях о консенсусе, полученных от Австралии [9], Канады [10], Швеции [11] и Швейцарии [12].

Возможность передачи: обзор

ВИЧ не передается легко от одного человека другому. Это относительно нестойкий вирус, который передается определенными хорошо описанными путями. Он не передается воздушно-капельным, капельным, контактным, трансмиссивным путем или через фомиты и не может проникать в неповрежденную кожу человека [13].

Для передачи ВИЧ должны существовать определенные основные условия:

- должно быть достаточное количество вируса в конкретных жидкостях организма (то есть в крови, сперме, предсеменной жидкости, ректальных жидкостях, вагинальных жидкостях или грудном молоке);
- достаточное количество, по меньшей мере, одной из этих жидкостей организма должно вступить в непосредственный контакт с участками в организме ВИЧ-

отрицательного человека, где может быть начато заражение. Это обычно слизистые оболочки, поврежденная ткань или воспаленные язвы, но не неповрежденная кожа;

- вирус должен преодолеть врожденную иммунную защиту человека, чтобы инфекция могла закрепиться и распространиться.

Большинство повседневных действий не сопряжены с риском передачи ВИЧ, поскольку для этого нет условий. Оставляя в стороне парентеральный или вертикальный пути передачи, для передачи обычно требуется интимный контакт, такой как половой акт. Даже в этих случаях вероятность передачи во время одного акта варьируется от нулевой до низкой (оценочно от 0 до 1,4% во время одного акта) [5].

Факторы, влияющие на возможность передачи ВИЧ

Возможность передачи ВИЧ, связанная с отдельными действиями, варьируется в зависимости от ряда пересекающихся факторов. При наличии нескольких пересекающихся факторов их действие минимизируется или усиливается в различной степени [14].

- Правильное использование презерватива предотвращает передачу ВИЧ

Правильное использование презерватива (мужского или женского) предотвращает передачу ВИЧ, поскольку пористость презервативов такова, что она обеспечивает защиту даже от самых маленьких патогенов, передающихся половым путем, включая ВИЧ [15]; латексные и полиуретановые презервативы действуют как непроницаемый физический барьер, через который ВИЧ пройти не может. Правильное использование презерватива означает, что его целостность не нарушена и презерватив используется на протяжении всего соответствующего полового акта. Правильное использование презерватива во время секса означает, что передача ВИЧ невозможна.

Исследования на уровне населения показали, что последовательное использование презервативов для анального или вагинального секса значительно снижает возможность передачи ВИЧ даже при таких факторах, как случаи неправильного использования или повреждения [16-21]. Например, метаанализ 14 исследований показал, что длительные периоды последовательного использования мужских презервативов во время вагинального секса уменьшают возможность передачи ВИЧ по меньшей мере на 80% [22]. Однако более поздние исследования показывают, что это

может быть недооценено [23], так как описанный метаанализ включал нестандартные методы анализа данных, которые могли привести к отклонению, вызванному набором участников исследований, и к другим отклонениям, способным снизить наблюдаемый уровень предотвращения [22,23].

Исследования на уровне населения применимы только в случаях, когда имели место множественные половые акты, и неизвестно, правильно ли использовались презервативы в каждом случае. Оценка на уровне населения, дающая 80% эффективность презерватива, не является независимой оценкой риска передачи ВИЧ, а должна применяться в отношении риска, связанного с различными половыми актами. Например, если оцениваемый риск передачи ВИЧ от ВИЧ-положительного мужчины женщине во время одного эпизода вагинального секса без презерватива составляет 0,08% [5], тогда риск передачи при использовании презерватива может быть понят как *по меньшей мере* на 80% ниже, то есть 0,016% (менее 2 на 10 000) [5]. Важно отметить, что, когда присутствуют другие факторы снижения риска (например, низкая вирусная нагрузка или прерывание полового акта до эякуляции), возможность передачи ВИЧ, даже в случае неправильного использования презерватива, еще больше снижается.

Повторимся, что ВИЧ не может передаваться в случаях, когда презерватив используется правильно (т. е. он используется на протяжении всего соответствующего полового акта, и его целостность не нарушена). Оценки на уровне населения могут применяться только в ситуациях, когда имеют место множественные случаи использования презервативов, включая происходящие иногда случаи неправильного использования и повреждения.

- Вирусная нагрузка, являющаяся низкой или «неопределяемой», значительно снижает или исключает возможность передачи ВИЧ

  Сразу после приобретения человеком ВИЧ его вирусная нагрузка очень высока, но она обычно снижается в течение первых нескольких недель, когда реагирует иммунная система человека. Если человек не начинает лечение, его вирусная нагрузка остается довольно стабильной в течение некоторого времени, тогда как иммунная система постепенно истощается. При прогрессирующей ВИЧ-инфекции вирусная нагрузка обычно снова увеличивается до более высоких уровней.

Антиретровирусная терапия предотвращает репликацию ВИЧ, тем самым значительно уменьшая вирусную нагрузку в жидкостях организма человека. С началом эффективной антиретровирусной терапии вирусная нагрузка обычно падает до уровней, не обнаруживаемых с помощью существующих стандартных лабораторных анализов крови в течение нескольких недель или месяцев. Доступность анализов и нижние пределы обнаружения различаются в разных частях мира, при этом нижние пределы обнаружения варьируются от примерно 20 копий вируса/мл до 400 копий /мл. Небольшой процент людей, живущих с ВИЧ (часто называемых длительными непрогрессорами), имеют низкую вирусную нагрузку без антиретровирусной терапии, потому что их иммунная система способна контролировать ВИЧ [24-28].

Уменьшенная вирусная нагрузка улучшает иммунную функцию и значительно снижает долгосрочную вероятность заболевания и смерти. Это также значительно снижает вероятность передачи ВИЧ [29-31]. Снижение вирусной нагрузки связано с сопутствующим снижением вероятности передачи ВИЧ [32-35], что означает, что многие люди, получающие лечение, не могут передавать ВИЧ.

Недавние анализы в рамках ключевых исследований (а именно HPTN052, PARTNER и Opposites Attract), в которых участвовали как гетеросексуальные, так и мужские пары с различным статусом ВИЧ, не выявили случаев передачи половым путем от человека с неопределяемой вирусной нагрузкой [29,30,36,37]. Эти результаты изменили содержание сообщений от организаций общественного здравоохранения. Например, Центры по контролю и профилактике заболеваний в США теперь описывают предполагаемую возможность передачи ВИЧ от ВИЧ-положительного человека с неопределяемой вирусной нагрузкой (в результате эффективного антиретровирусного лечения) как «фактически без риска» [6].

В 2011 году в исследовании HPTN052 (проведенном в Ботсване, Бразилии, Индии, Кении, Малави, ЮАР, Таиланде, США и Зимбабве), в котором исследовалось влияние раннего начала лечения, не наблюдалась передача ВИЧ от 1763 человек, проходящих антиретровирусную терапию, которые имели стабильную вирусную нагрузку ниже 400 копий/мл. Были прослежены партнеры ВИЧ-положительных участников в эквиваленте 8509 человеко-лет.

Единственная передача от людей, проходящих лечение, произошла либо в начале лечения (до того, как вирусная нагрузка стабилизировалась на уровне ниже 400 копий), либо когда вирусная нагрузка была выше 1000 копий/мл в ходе двух последовательных визитов [29,37].

Исследования PARTNER и Opposites Attract не выявили передачи ВИЧ от людей с вирусной нагрузкой ниже 200 копий/мл после более чем 75 000 актов вагинального или анального секса без презерватива [18,30,38]. В исследовании PARTNER гетеросексуальные пары сообщили примерно о 36 000 половых актах без презерватива, а гомосексуальные мужские пары сообщили о 22 000 половых актов без презерватива [30]. Передача ВИЧ между партнерами в исследовании не произошла. Тем не менее было одиннадцать случаев нового ВИЧ-инфицирования, однако филогенетический анализ обнаружил, что во всех случаях инфекция была вызвана половым контактом с кем-то, кроме постоянного полового партнера. Исследование Opposites Attract включало в себя около 17 000 половых актов без презерватива между мужчинами. Не сообщалось о передаче ВИЧ между партнерами, участвовавшими в исследовании, при этом три случая нового ВИЧ-инфицирования были вызваны половым контактом с кем-то, кроме постоянного полового партнера человека [18].

В систематическом обзоре и метаанализе 2013 года также не было обнаружено никакой передачи, когда вирусная нагрузка падала ниже порогового значения от 50 до 500 копий/мл (в зависимости от исследования) [39]. В другом исследовании сообщалось об отсутствии передачи, когда вирусная нагрузка была ниже 400 копий/мл [40]. Ряд других исследований показал, что низкая (но обнаруживаемая) вирусная нагрузка значительно снижает (и может исключить) возможность передачи. Например, ранние исследования с участием лиц, которые не проходили антиретровирусную терапию, не выявили случаев передачи среди пар, в которых один из партнеров жил с ВИЧ и имел низкую, но обнаруживаемую вирусную нагрузку: ниже 1500 копий/мл (Уганда) [32], ниже 1094 копий/мл (Таиланд) [33] и ниже 1000 копий/мл (Замбия) [34]. Угандийское исследование показало, что вероятность передачи через вагинальный половой акт, когда вирусная нагрузка была ниже 1700 копий/мл, составила 1 на 10 000 [41].

Хотя у многих людей, проходящих антиретровирусную терапию [42,43], наблюдаются непродолжительные увеличения вирусной нагрузки небольшой амплитуды, известные как «всплески», они не свидетельствуют о том, что ВИЧ-терапия «терпит неудачу»; не считаются клинически значимыми; и не было показано, что они увеличивают возможность передачи ВИЧ во время секса [44,45]. Крупномасштабные исследования среди пар с различным статусом ВИЧ включали много ВИЧ-положительных участников, которые в ходе исследования испытали всплески своей вирусной нагрузки. Соответственно, такие всплески были учтены в наблюдаемом снижении количества передач.

Доконтактная профилактика (ДКП) значительно снижает возможность заражения ВИЧ.
ДКП относится к использованию антиретровирусных препаратов ВИЧ-отрицательными людьми до подвергания риску ВИЧ, чтобы предотвратить передачу ВИЧ [46-50]. Одно недавнее исследование показало, что ДКП эффективна до 95% среди придерживающихся ее пользователей [50], однако было описано только небольшое количество случаев неудачи ДКП у тех, кто придерживался ее, а это дает основания предполагать, что эффективность ДКП, вероятно, превышает 95%.

- Постконтактная профилактика (ПКП) значительно снижает возможность приобретения ВИЧ
  ПКП относится к кратковременному использованию антиретровирусного лечения ВИЧ-отрицательным человеком после подвергания риску ВИЧ. Если ПКП началась в течение 72 часов после подвергания риску, проводилась в течение 28 дней и хорошо соблюдалась, она значительно уменьшает вероятность того, что человек станет ВИЧ-положительным, потому что она может остановить закрепление ВИЧ в иммунных клетках человека даже после того, как вирус проник в его организм [51,52]. Хотя ПКП не эффективна на 100%, сообщалось о высоких показателях успеха [51,53-67] (например, 81% среди пациентов, использовавших более старые методы лечения [67], и до 100% среди пациентов, использовавших более новые методы лечения [68]). Эффективность ПКП, по-видимому, зависит от ряда факторов; при этом

эффективность, как правило, повышается при более раннем начале ПКП, так как количество ВИЧ, попадающего в организм человека, уменьшается [68].

- Обрезание мужчин по медицинским показаниям снижает возможность передачи ВИЧ от женщин мужчинам
  Обрезание мужчин по медицинским показаниям снижает возможность передачи ВИЧ от ВИЧ-положительных женщин ВИЧ-отрицательным мужчинам примерно на 50% [69]. Обрезание также может снизить передачу ВИЧ половым путем среди мужчин, имеющих секс с мужчинами, для ВИЧ-отрицательных мужчин, которые являются исключительно проникающими партнерами, хотя исследования не являются окончательными [70].

- Методы снижения риска, такие как прерывание полового акта или стратегическое позиционирование, снижают возможность передачи ВИЧ
  Некоторые люди, живущие с ВИЧ, используют методы снижения риска, такие как прерывание полового акта до эякуляции или стратегическое позиционирование (т. е. анальный половой акт только в принимающей позиции) при занятии сексом без презерватива с ВИЧ-отрицательным человеком или человеком неизвестного серостатуса [71-73]. Такие действия уменьшают вероятность передачи ВИЧ во время секса, когда возможность передачи существует [71]. Например, исследование 2010 года показало, что вероятность передачи во время анального секса уменьшалась примерно на две трети, когда ВИЧ-позитивный проникающий партнер не эякулировал [73]. Также известно, что возможность передачи ниже, когда ВИЧ-положительный партнер является принимающим, а не проникающим партнером во время анального секса [73-75].

- Инфекции, передающиеся половым путем (ИППП), могут в некоторых случаях увеличивать возможность передачи ВИЧ
  Наличие некоторых нелеченных ИППП, особенно язвенных ИППП, у любого из партнеров связано с повышенной вероятностью передачи ВИЧ во время сексуальной активности, когда человек, живущий с ВИЧ, не имеет низкой вирусной нагрузки [76]. Когда генитальные язвы присутствуют у обоих партнеров, риск еще больше увеличивается [14]. Однако наличие ИППП не увеличивает возможность передачи, если ВИЧ-положительный человек

проходит эффективную антиретровирусную терапию [30] *или* если ВИЧ-отрицательный человек проходит ДКП[48,49].

Возможность передачи ВИЧ при сексуальном контакте

Передача ВИЧ при сексуальном контакте обычно происходит в результате контактирования жидкостей организма, содержащих достаточно ВИЧ, со слизистыми оболочками, расположенными в: крайней плоти или уретре полового члена; шейке матки или влагалище; анусе; или прямой кишке. Передача ВИЧ также возможна при контакте со слизистыми оболочками ротовой полости, но они гораздо менее уязвимы для передачи ВИЧ [58].

Оральный секс, включая орально-пенильный и орально-вагинальный секс

- Вероятность передачи ВИЧ через оральный секс, выполняемый для ВИЧ-положительного человека, в том числе, когда человек не имеет низкой вирусной нагрузки и/или не используется презерватив, варьируется от невозможности до незначительной возможности в зависимости от контекста [77,78].

Оральный секс пропагандируется как вариант безопасного секса для партнеров с различным статусом ВИЧ, желающих участвовать в интимных половых актах; причем его практика, как сообщается, очень распространена.

Известно, что оральный секс имеет гораздо более низкую возможность передачи ВИЧ, чем вагинальный или анальный половой акт [79,80]. Фактически риск передачи ВИЧ в результате орального секса настолько низок, что ученые не смогли сделать статистически обоснованную оценку.

Несколько клинических исследований, изучавших передачу при оральном сексе, не смогли найти ни одного случая передачи ВИЧ [74,81,82]. Исследование гетеросексуальных пар и исследование лесбийских пар не обнаружило передачи в результате орального секса [81,82]. Третье исследование, в котором участвовали мужчины, имевшие секс с мужчинами, не выявило сероконверсии среди участников, сообщавших о выполнении только фелляции (с эякуляцией) с мужчинами, которые были ВИЧ-положительными или имели неизвестный ВИЧ-статус [74]. Статистическая модель, примененная к этим результатам, показала, что риск при контакте с оральным сексом находится в диапазоне от нуля до 0,04% (4 на 10 000) [78], и эти значения используются в некоторых отчетах [79,80,83]. Учитывая, что исследование не выявило

сероконверсии, верхняя граница 0,04% может быть понята как верхняя граница возможности.

- Не существует возможности передачи ВИЧ при оральном сексе, выполняемом для ВИЧ-положительного человека, когда ВИЧ-положительный партнер имеет низкую вирусную нагрузку **или** презерватив используется правильно, **или** ВИЧ-отрицательный партнер проходит ДКП[78].

Несмотря на то, что нет исследований, посвященных изучению влияния антиретровирусной терапии или ДКПна возможность передачи во время орального секса, по нашему экспертному мнению, не существует возможности передачи ВИЧ в связи с оральным сексом, выполняемым для ВИЧ-положительного человека, проходящего антиретровирусную терапию, или выполняемым лицом, проходящим ДКП. Аналогичным образом, правильное использование презерватива снижает вероятность передачи ВИЧ до нуля.

Пенильно-вагинальный половой акт

- Возможность передачи ВИЧ через пенильно-вагинальный половой акт, когда ВИЧ-положительный партнер не имеет низкой вирусной нагрузки **и** не используется презерватив, является низкой [84]. Вероятность передачи уменьшается еще больше, если у ВИЧ-отрицательного партнера не происходит эякуляции.

Два метаанализа гетеросексуальных пар [14,84] обнаружили, что вероятность передачи ВИЧ во время одного вагинального полового акта является низкой: 0,08% (8 на 10 000) при отсутствии сопутствующих факторов риска [5,14,41,84]. Неясно, выше ли вероятность передачи ВИЧ от мужчины женщине во время вагинального полового акта, чем вероятность передачи от женщины к мужчине. Некоторые исследования не обнаружили разницы, в то время как другие предполагают, что возможность передачи ВИЧ от мужчины к женщине примерно в два раза выше, чем передача от женщины к мужчине [14,35,83,84].

- Вероятность передачи ВИЧ через пенильно-вагинальный половой акт, когда ВИЧ-положительный партнер имеет низкую вирусную нагрузку **или** использует презерватив **или** ВИЧ-отрицательный партнер проходит ДКП, варьируется от невозможности до незначительной возможности в зависимости от контекста [29,38].

Многочисленные исследования, которые обсуждались выше, показали, что вероятность передачи ВИЧ от ВИЧ-положительного партнера, имеющего низкую вирусную нагрузку, через пенильно-вагинальный половой акт варьируется от невозможности до незначительной возможности [29,37-39,85]. Ни в одном клиническом исследовании не сообщалось о случае передачи через пенильно-вагинальный половой акт от человека с необнаруживаемой вирусной нагрузкой.

ВИЧ не может передаваться при правильном использовании презерватива, потому что ВИЧ не может проходить через неповрежденный латекс или полиуретан. Аналогичным образом, невозможно передать ВИЧ, если человек имеет необнаруживаемую вирусную нагрузку.

Пенильно-анальный половой акт

- Возможность передачи ВИЧ, когда не используется презерватив **и** ВИЧ-положительный партнер не имеет низкой вирусной нагрузки, является низкой [86]. Риск не зависит от того, является ли принимающий партнер мужчиной или женщиной. Риск ниже, когда ВИЧ-положительный человек принимает роль принимающего, а не проникающего партнера. Риск также ниже, если ВИЧ-положительный проникающий партнер не эякулирует внутри принимающего партнера.

Исследования показывают, что принимающий анальный половой акт без презерватива у гетеросексуальных или однополых пар связан с более высокой вероятностью передачи ВИЧ, чем принимающий вагинальный половой акт без презерватива [5,87,88]. Согласно оценкам отдельных исследований, вероятность передачи ВИЧ во время одного акта анального секса варьируется от 0,01% (1 на 10 000) до более чем 3% (300 на 10 000) [20,75,84,88-91]. Вероятность передачи принимающему партнеру от проникающего выше, чем наоборот [18,74,84].

Согласно оценкам двух систематических обзоров (2010 года и 2014 года), вероятность передачи во время одного акта составляет приблизительно 1,4% (140 на 10 000) для принимающего анального секса (т. е., когда ВИЧ-положительный человек является проникающим партнером) [5,86]. Проспективное когортное исследование 2010 года показало, что вероятность снижается с 1,43% (143 на 10 000) с эякуляцией до 0,54% (54 на 10 000) без эякуляции [89]. Согласно оценкам, вероятность передачи во время одного акта составляет 0,11% (11 на 10 000), когда ВИЧ-отрицательный человек является проникающим партнером [5].

- Вероятность передачи ВИЧ через пенильно-анальный половой акт, когда ВИЧ-положительный партнер имеет низкую вирусную нагрузку **или** использует презерватив **или** ВИЧ-отрицательный партнер проходит ДКП, варьируется от невозможности до незначительной возможности в зависимости от контекста [85,86].

Существует незначительная возможность передачи ВИЧ от ВИЧ-положительного партнера, имеющего низкую вирусную нагрузку, во время пенильно-анального полового акта. Как обсуждалось выше, как в исследовании PARTNER, так и в исследовании Opposites Attract не наблюдалось никакой передачи после приблизительно 39 000 актов анального секса без презерватива, когда вирусная нагрузка была ниже 200 копий/мл [30,92]. Фактически ни в одном клиническом исследовании не сообщалось о случае передачи от человека с неопределяемой вирусной нагрузкой.

ВИЧ не может передаваться при правильном использовании презерватива, потому что ВИЧ не может проходить через неповрежденный латекс или полиуретан. Аналогичным образом, невозможно передать ВИЧ, если человек имеет неопределяемую вирусную нагрузку.

Возможность передачи ВИЧ через повседневный контакт, плевание и кусание

Повседневный контакт

ВИЧ не может передаваться через контакт с такой поверхностью в окружающей среде, как стул, скамейка или туалет; от еды или напитка; или от повседневных контактов с людьми, таких как обнимание, совместное использование предметов домашнего обихода или совместное питание.

ВИЧ не может долго выжить на воздухе и не способен проникать в неповрежденную кожу. Ни один случай ВИЧ-инфицирования через контакт с поверхностью в окружающей среде, едой или напитком или через повседневный контакт с человеком никогда не был идентифицирован, несмотря на многие научные исследования, рассматривающие эту возможность [93-98].

Кусание и плевание

- Не существует возможности передачи ВИЧ через контакт со слюной ВИЧ-положительного человека, в том числе через поцелуи, кусание или плевание.

В многочисленных исследованиях была рассмотрена возможность передачи ВИЧ через слюну, но ни одно из них не обнаружило никаких доказательств, включая

исследование 34 000 случаев в Великобритании в 1997 году [99]. Отсутствие передачи ВИЧ через слюну объясняется двумя факторами: слюна содержит очень небольшое количество ВИЧ [100], а несколько ингибирующих компонентов в секретах ротовой полости означают, что слюна защищает восприимчивые клетки от ВИЧ-инфекции [101-106].

- Не существует возможности передачи ВИЧ при кусании или плевании, когда слюна ВИЧ-положительного человека не содержит крови или содержит небольшое количество крови.

Текущие доказательства свидетельствуют, что ВИЧ не может передаваться даже тогда, когда слюна содержит небольшое количество крови. Несмотря на ранние исследования, предполагающие теоретический риск передачи при попадании содержащей кровь слюны в организм человека через контакт со слизистой тканью (например, при ее попадании в глаз или рот), не сообщалось о случаях передачи ВИЧ вследствие плевка кровью [107]. Соответственно, по нашему экспертному мнению, невозможно пер едать ВИЧ через слюну, содержащую небольшое количество крови.

- Вероятность передачи ВИЧ через кусание, когда слюна ВИЧ-положительного человека содержит значительное количество крови и его кровь вступает в контакт со слизистой оболочкой или открытой раной, и при этом его вирусная нагрузка не является низкой или неопределяемой, варьируется от невозможности до незначительной возможности.

Во многих исследованиях было подробно описано большое количество случаев, когда укусы не привели к передаче ВИЧ [108-112], или было обнаружено, что передача маловероятна [103,109,113,114].

Для того чтобы передача в случае кусания была правдоподобной, ВИЧ-положительный человек должен иметь кровь во рту во время укуса, в крови ВИЧ-положительного человека должно присутствовать достаточное количество ВИЧ, а укус должен быть достаточно глубоким, чтобы проникнуть в кожу ВИЧ-отрицательного человека, вызвав травму и повреждение тканей [106,107,115]. Даже когда все эти условия присутствуют, возможность передачи при одном укусе не больше, чем незначительная.

Значительные улучшения ожидаемой продолжительности жизни и качества жизни людей, живущих с ВИЧ

Во втором разделе этого Заявление о консенсусе рассматривается вред, связанный с ВИЧ, потому что стойкие заблуждения, преувеличивающие вред ВИЧ-инфекции, по-

видимому, влияют на применение уголовного права [3]. Уголовное право принимает во внимание возможный вред, причиняемый потенциальным правонарушением, а также вероятность самого правонарушения; таким образом, например, определения вреда здоровью отличаются от определений тяжкого вреда здоровью, которые отличаются от определений причинения смерти по неосторожности или убийства.. Соответственно, важно подчеркнуть огромное изменение перспектив для людей, живущих с ВИЧ, которые были достигнуты за последние десятилетия.

Естественный ход нелеченной ВИЧ-инфекции широко варьируется в зависимости от конкретного человека [116]. При отсутствии лечения большинство людей испытывают бессимптомную фазу длительностью от двух до 15 лет, во время которой вирус реплицируется, постепенно подрывая их иммунную систему. Небольшой процент людей с ВИЧ имеют иммунную систему, которая блокирует репликацию вируса на неопределенное время [117], но у большинства людей при отсутствии лечения в конечном итоге развивается СПИД (примерно у половины в течение 10 лет [118]). СПИД определяется как наличие определенных лабораторных маркеров и/или оппортунистических инфекций и конкретных заболеваний, которые, если антиретровирусная терапия не начата, в конечном итоге приводят к смерти человека.

Антиретровирусная терапия значительно снижает прогрессирование ВИЧ-ассоциированных заболеваний. В глобальном масштабе руководства по лечению были пересмотрены таким образом, чтобы рекомендовать начало антиретровирусного лечения сразу после диагностики ВИЧ-инфекции, поскольку у большинства людей при лечении будет достигаться неопределяемая вирусная нагрузка, поддерживаться здоровая иммунная система, сохраняться здоровье и будут предотвращаться осложнения длительной ВИЧ-инфекции [119,120]. Даже те, кто начинают лечение с высокой вирусной нагрузкой и прдерживаются терапии, могут ожидать значительного снижения вирусной нагрузки до такой степени, что происходит значительное восстановление иммунной системы, позволяющее им наслаждаться хорошим здоровьем в течение длительного периода времени [121]. Для многих эффективное лечение требует приема по одной таблетке каждый день.

Исследования, проведенные во многих странах, последовательно показывают, что антиретровирусная терапия значительно увеличивает ожидаемую продолжительность жизни, что ожидаемая продолжительность жизни и далее улучшается с течением

времени и что здоровье и качество жизни людей, живущих с ВИЧ, в долгосрочной перспективе значительно улучшились [122-141]. Ожидаемая продолжительность жизни молодых людей с ВИЧ, начинающих антиретровирусную терапию, теперь приближается к ожидаемой продолжительности жизни молодого человека в общем населении [45,132,134,135,137]. Кроме того, использование антиретровирусной терапии сместило причину смерти людей, живущих с ВИЧ, от традиционных болезней, определяющих СПИД, в сторону факторов, не связанных с ВИЧ [142,143], аналогичных тем, которые затрагивают население в целом [144]. Аналогичным образом, клинический менеджмент сместился в сторону менеджмента и лечения проблем со здоровьем, связанных со старением, включая менопаузу и сердечно-сосудистое заболевание [143-150], а также вмешательства, влияющие на «выбор образа жизни», такие как табакокурение [151]. В некоторых подгруппах населения постоянный клинический уход имеет потенциал увеличения ожидаемой продолжительности жизни людей, живущих с ВИЧ, сверх ожидаемой продолжительности жизни ВИЧ-отрицательных людей [135].

Хотя ВИЧ вызывает инфекцию, которая требует непрерывного лечения с помощью антиретровирусной терапии, люди, живущие с ВИЧ, могут жить долго и продуктивно, в том числе работать, учиться, путешествовать, иметь отношения, иметь и воспитывать детей, а также вносить вклад в общество разными другими способами.

Установление доказательств передачи ВИЧ

В заключительном разделе этого Заявления о консенсусе признается важность правильного использования научных и медицинских доказательств в связанных с ВИЧ судебных преследованиях, где речь идет о доказательстве фактической передачи от одного человека другому.

Международное руководство по ВИЧ в контексте уголовного права рекомендует, что «доказательство причинной связи в отношении передачи ВИЧ всегда должно основываться на доказательствах, полученных из многочисленных соответствующих источников, включая медицинские записи, строгие научные методы и сексуальную историю» [1].

- Медицинские записи могут предоставлять контекстуальную информацию, но не могут установить передачу между заявителем и обвиняемым.

Обстоятельства характера и сроков сексуальных отношений или других потенциальных источников ВИЧ-инфекции человека должны занимать центральное

место в любом деле, где предполагается передача ВИЧ половым путем. Когда медицинские записи доступны и получены законно, они ценны для определения последнего ВИЧ-отрицательного и первого ВИЧ-положительного теста заявителя и обвиняемого. Рассматривая период диагностического окна каждого теста, эту информацию можно использовать для установления периода, в течение которого заявитель приобрел ВИЧ, и был ли обвиняемый ВИЧ-положительным в течение этого времени. Важно отметить, что то, кто был инфицирован первым: заявитель или обвиняемый, не может быть основано на том, у кого первого был положительный тест на ВИЧ или кто предъявил обвинения другому.

Информация, относящаяся к вирусной нагрузке ВИЧ и подсчетам CD4, содержащаяся в медицинских записях, иногда представлялась как доказательство установления сроков ВИЧ-инфицирования. Однако вирусная нагрузка и подсчеты CD4 показывают значительную межиндивидуальную и внутрииндивидуальную изменчивость и поэтому не могут быть использованы для точного определения того, когда кто-то приобрел ВИЧ [152].

- Филогенетический анализ может использоваться в качестве судебного инструмента. Результаты могут быть совместимы с утверждением, но не могут окончательно доказать утверждение, что обвиняемый инфицировал заявителя. Важно отметить, что филогенетические результаты могут оправдать обвиняемого, когда результаты несовместимы с утверждением, что обвиняемый инфицировал заявителя.

Филогенетический анализ сравнивает эволюционные отношения между ВИЧ разных людей, но результаты следует интерпретировать осторожно наряду с другими фактическими и медицинскими доказательствами, когда они используются в уголовных делах [153]. Сложность филогенетического анализа возникает, в частности, из-за того, что ВИЧ является быстроразвивающимся вирусом. Мутации вируса происходят неоднократно, так что каждый человек, живущий с ВИЧ, имеет более одного варианта вируса [154]. Во время передачи передается ограниченное количество вариантов вируса (от одного до нескольких), но они также будут мутировать, формируя новые варианты, так что не существует двух людей с идентичным ВИЧ [155].

Филогенетический анализ ВИЧ включает в себя оценку эволюционных отношений вариантов ВИЧ, например, в рамках исследования сетей передачи ВИЧ для целей общественного здравоохранения. В уголовных делах филогенетический анализ

включает в себя исследование того, являются ли заявитель(и) и обвиняемый(е) частью одной и той же сети передачи. Сеть представляется в виде филогенетического «дерева». Примечательно, что филогенетическое дерево следует понимать как дерево гена ВИЧ, которое может отличаться от истории передачи, поскольку варианты ВИЧ могут предшествовать передаче или исчезнуть после передачи [156] и поскольку некоторые люди в сети передачи, возможно, не проходили диагностику и/или у них не брались пробы перед построением дерева.

Филогенетика ВИЧ очень отличается от профилирования ДНК человека, поскольку, учитывая продолжающуюся эволюцию вариантов ВИЧ каждого человека, филогенетика не может получить «точное соответствие». Когда, по-видимому, существует «филогенетическое соответствие» между ВИЧ двух людей, это означает, что два или более вариантов эпидемиологически «связаны», а не то, что они одинаковы [155,157]. Филогенетические доказательства ВИЧ *могут* оправдать обвиняемого, обвиненного в передаче ВИЧ заявителю, потому что, если вирусные штаммы, обнаруженные у обвиняемого и заявителя, не связаны, филогенетические доказательства окончательно противоречат утверждению, что обвиняемый был источником вируса заявителя. [155,158].

Недавние достижения в секвенировании ДНК и филогенетике позволяют высказывать некоторые соображения в отношении направления и сроков передачи [159-162], но эти методы в настоящее время не являются ни тщательными, ни достаточно точными для доказательства того, кто кого инфицировал [155,163]. Это отчасти потому, что в сети передачи всегда могут быть неизвестные и недиагностированные люди [155]. Соответственно, в настоящее время филогенетический анализ не может исключить возможность того, что заявитель заразил обвиняемого или что оба они были инфицированы третьим лицом [158,163], или более сложные сценарии передачи, которые привели к тому, что обвиняемый и заявитель имели варианты ВИЧ, которые связаны эпидемиологически. Сложности добавляет тот факт, что наличие ВИЧ не защищает от последующего «суперинфицирования» другим вариантом [158]. В частности, доверие к направлению заражения подрывается, когда обвиняемый и заявитель участвуют в многочисленных половых актах, которые могут способствовать многократным событиям передачи туда и обратно [155].

Филогенетический анализ является сложным, и, соответственно, важно, чтобы филогенетика ВИЧ для судебных целей выполнялась и интерпретировалась экспертами, которые полностью понимают ограничения техники и ясно указывают на эти

ограничения в письменных отчетах и устных показаниях. Интерпретация филогенетических результатов для судебных целей требует экспертных знаний в области филогенетики и различий между деревьями эволюции и историями передачи вируса. Это не так просто, и методологии еще не стандартизированы [155]. Надежность доказательств, полученных в результате филогенетического анализа, зависит от ряда методологических факторов, включая использование адекватных «локальных элементов контроля» [164-166] и последовательностей баз данных [167-169], которые должны выбираться с использованием последовательных критериев отбора [155]. Международные исследования показывают, что филогенетические доказательства, используемые в уголовных процессах, не всегда удовлетворяют этим требованиям [155].

Заключение

Учитывая доказательства, представленные в этом документе, мы настоятельно рекомендуем проявлять большую осторожность при решении вопроса о возбуждении уголовного производства, включая тщательную оценку текущих научных доказательств рисков и вреда, связанных с ВИЧ. Это помогает уменьшить стигму, дискриминацию и избежать судебных ошибок.

В этом контексте мы надеемся, что это Заявление о консенсусе побудит представителей власти, сотрудников правоохранительной и судебной систем, при любом применении уголовного права в делах, связанных с ВИЧ, уделять пристальное внимание значительным достижениям в области науки о ВИЧ последних тридцати лет , и прилагать все усилия для обеспечения правильного и полного понимания текущих научных знаний.

Конфликт интересов

Питер Годфри-Фауссет работает в Лондонской школе гигиены и тропической медицины и прикомандирован к ЮНЭЙДС на условиях полной занятости. Все остальные авторы заявляют, что у них нет конфликта интересов.

Выражение признательности

Мы признательны за поддержку Салли Кэмерон, Эдвину Бернарду, Луизе Кабал, Стефани Клайваз-Лорангер, Патрику Эба, Ричарду Эллиоту, Сесиль Казачкин, Дэвиду Маклаю, Кевину Осборну, Марианджеле Симау и Лорел Спраг.

Финансирование

Финансирование этой работы было предоставлено Международным обществом борьбы со СПИДом (IAS), Международной ассоциацией поставщиков услуг по уходу в

сфере СПИДа (IAPAC), Фондом поддержки сетей гражданского общества имени Роберта Карра и ЮНЭЙДС.

Вклад авторов

Все авторы участвовали в многочисленных раундах обсуждений, написании и редактировании этого Заявления о консенсусе.

Список справочной литературы

1. Joint United Nations Programme on HIV/AIDS (UNAIDS). UNAIDS guidance note on ending overly broad HIV criminalisation. Geneva: UNAIDS; 2013.
2. Глобальная комиссия по ВИЧ и законодательству. ВИЧ и законодательство: риски, права и здоровье. Женева: ПРООН; 2012.
3. Bernard EJ, Cameron S. Advancing HIV Justice 2: Building momentum in global advocacy against HIV criminalization. HIV Justice Network, Global Network of People Living with HIV; April 2016.
4. World Health Organization. Sexual health, human rights and the law. Geneva: World Health Organization; 2015.
5. Patel P, Borkowf CB, Brooks JT, Lasry A, Lansky A, Mermin J. Estimating per-act HIV transmission risk: a systematic review. AIDS. 2014 Jun 19; 28(10):1509-19.
6. Centers for Disease Control and Prevention. CDC Information Undetectable Viral Load and HIV Transmission Risk. October 2017. Доступно на https://www.cdc.gov/hiv/pdf/risk/art/cdc-hiv-uvl-transmission.pdf
7. 2008 MBQB 201; Canada.
8. R v Mabior. SCC 47. 2012; Canada.
9. Boyd M, Cooper D, Crock E, Crooks L, Giles M, Grulich A, et al. Sexual transmission of HIV and the law: an Australian medical consensus statement. Med J Aust. 2016; 205(9):409-12.
10. Loutfy M, Tyndall M, Baril J-G, Montaner J, Kaul R, Hankins C. Canadian consensus statement on HIV and its transmission in the context of the criminal law. Can J Infect Dis Med Microbiol. 2014; 25(3):135–40.
11. Albert J, Berglund T, Gisslén, M, Gröön P, Sönnerborg A, Tegnell A, et al. Risk of HIV transmission from patients on antiretroviral therapy: a position statement from the Public Health Agency of Sweden and the Swedish Reference Group for Antiviral Therapy. Scandinavian Journal of Infectious Diseases. 2014; 46(10):673-7.
12. Vernazza P, Hirschel B, Bernasconi E, Flepp M. Les personnes séropositives ne souffrant d’aucune autre MST et suivant un traitment antirétroviral efficace ne transmettent pas le VIH par voie sexuelle. Bulletin des médecins suisses. 2008 30; 89(5), 165-9. На французском языке.
13. Vandamme A-M, Van Laethem K, Schmit J-C, Van Wijngaerden E, Reynders M, Debyser Z, et al. Long-term stability of human immunodeficiency virus viral load and infectivity in whole blood. European Journal of Clinical Investigation. 1999; 29:445-52.
14. Powers K, Poole C, Pettifor A, Cohen M. Rethinking the heterosexual infectivity of HIV-1: a systematic review and meta-analysis. Lancet Infect Dis. 2008 Sep; 8(9):553-63.
15. Lytle CD. An in vitro evaluation of condoms as barriers to a small virus. Sex Transm Dis. 1997; 24:161–4.
16. Vittinghoff E, Douglas J, Judson F, McKirnan D, MacQueen K and Buchbinder SP. Per-contact risk of human immunodeficiency virus transmission between male sexual partners. Am J Epidemiol. 1999 Aug 1; 150(3):306-11.
17. Weller S, Davis K. Condom effectiveness in reducing heterosexual HIV transmission. Cochrane Database Syst Rev.[Интернет]. 2001 [цитировано 6 июня 2018 г.] Доступно на: http://onlinelibrary.wiley.com
18. Macdonald N, Elam G, Hickson F, Imrie J, McGarrigle CA, Fenton KA, et al. Factors associated with HIV seroconversion in gay men in England at the start of the 21st century. Sex Transm Infect. 2008 Feb; 84(1):8-13.

19. Lavoie E, Alary M, Remis RS, Otis J, Vincelette J, Turmel B. et al. Determinants of HIV seroconversion among men who have sex with men living in a low HIV incidence population in the era of highly active antiretroviral therapies. Sex Transm Dis. 2008 Jan; 35(1):25-9.

20. Scott HM, Vittinghoff E, Irvin R, Sachdev D, Liu A, Gurwith M et al. Age, Race/Ethnicity, and Behavioral Risk Factors Associated with Per-Contact Risk of HIV Infection Among Men Who Have Sex with Men in the United States. J Acquir Immune Defic Syndr. 2014 January 1;65(1): 115–121.

21. Smith DK, Herbst JH, Zhang X, Rose CE. Condom effectiveness for HIV prevention by consistency of use among men who have sex with men in the United States. J Acquir Immune Defic Syndr. 2015 Mar 1; 68(3):337-44. Referenced in doi:10.1097/QAI.0000000000000461.

22. Weller S, Davis-Beaty K. Condom effectiveness in reducing heterosexual HIV transmission. Cochrane Database Syst Rev 2002;(1):CD003255.

23. Crosby R, Bounse S. Condom effectiveness: where are we now?. Sex Health. 2012; 9:10-17

24. Buchbinder S, Katz M, Hessol N, O'Malley P, Homberg S. Long-term HIV-1 infection without immunologic progression. AIDS. 1994; 8:1123-8.

25. Madec Y, Boufassa F, Avettand-Fenoel V, Hendou S, Melard A, Boucherit S, et al. Early control of HIV-1 infection in long-term nonprogressors followed since diagnosis in the ANRS SEROCO/HEMOCO cohort. J Acquir Immune Defic Syndr. 2009; 50:19-26.

26. Poropatich K, Sullivan DJ. Human immunodeficiency virus type 1 long-term non-progressors: the viral, genetic and immunological basis for disease non-progression. Journal of General Virology. 2010; 2(2):247-68.

27. Learmont J, Geczy A, Mills J, Ashton L, Raynes-Greenow C, Garsia R, et al. Immunologic and virologic status after 14 to 18 years of infection with an attenuated strain of HIV-1. A report from the Sydney Blood Bank Cohort. N Engl J Med 1999;340:1715-22.

28. Rhodes DI, Ashton L, Solomon A, Carr A, Cooper D, Kaldor J, et al. Characterization of three nef-defective human immunodeficiency virus type 1 strains associated with long-term nonprogression. Australian Long-Term Nonprogressor Study Group. J. Virol. 2000 Nov:74(22):10581–8.

29. Cohen MS, Chen YQ, McCauley M, Gamble T, Hosseinipour MC, Kumarasamy N, et al. Prevention of HIV-1 infection with early antiretroviral therapy. N Engl J Med. 2011 Aug 11; 365:493-505.

30. Rodger AJ, Cambiano V, Bruun T, Vernazza P, Collins S, van Lunzen J, et al. Sexual activity without condoms and risk of HIV transmission in serodifferent couples when the HIV-positive partner is using suppressive antiretroviral therapy. JAMA. 2016; 316:171-81.

31. Montaner JS, Hogg R, Wood E, Kerr T, Tyndall M, Levy AR, et al. The case for expanding access to highly active antiretroviral therapy to curb the growth of the HIV epidemic. Lancet. 2006; 368(9534):531-6.

32. Quinn TC, Wawer MJ, Sewankambo N, Serwadda D, Li C, Wabwire-Mangen F, et al. Viral load and heterosexual transmission of human immunodeficiency virus type 1. Rakai Project Study Group. N Engl J Med. 2000; 342(13):921-9.

33. Tovanabutra S, Robison V, Wongtrakul J, Sennum S, Suriyanon V, Kingkeow D, et al. Male viral load and heterosexual transmission of HIV-1 subtype E in northern Thailand. J Acquir Immune Defic Syndr. 2002 Mar 1; 29(3):275-83.

34. Fideli US, Allen SA, Musonda R, Trask S, Hahn BH, Weiss H, et al. Virologic and immunologic determinants of heterosexual transmission of human immunodeficiency virus type 1 in Africa. AIDS Res Hum Retroviruses. 2001 Jul 1; 17(10):901-10.

35. Hughes JP, Baeten JM, Lingappa JR, Magaret AS, Wald A, de Bruyn G. Determinants of per-coital-act HIV-1 infectivity among African HIV-1-serodiscordant couples. J Infect Dis. 2012 Feb 1; 205(3):358-65.

36. Grulich A, Bavinton B, Jin F, Prestage G, Zablotska, Grinsztejn B, et al. HIV transmission in male serodiscordant couples in Australia, Thailand and Brazil. Abstract for 2015 Conference on Retroviruses and Opportunistic Infections, Seattle, USA, 2015.

37. Cohen MS, Chen YQ, McCauley M, Gamble T, Hosseinipour M, Kumarasamy N, et al. Antiretroviral Therapy for the Prevention of HIV-1 Transmission. N Engl J Med. 2016 Sep 1; 375(9):830-9.

38. Supervie V, Viard J-P, Costagliola D, Breban R. Heterosexual risk of HIV transmission per sexual act under combined antiretroviral therapy: systematic review and Bayesian modeling. Clin Infect Dis Off Publ Infect Dis Soc Am. 2014 Jul 1;59(1):115-22.

39. Loutfy MR, Wu W, Letchumanan L, Bondy L, Antoniou T, Margolese S, et al. Systematic Review of HIV Transmission between Heterosexual Serodiscordant Couples where the HIV Positive Partner Is Fully Suppressed on Antiretroviral Therapy. PLoS ONE. 2012 Feb 13;8(12).

40. Anglemyer A, Rutherford GW, Baggaley RC, Egger M, Siegfried N. Antiretroviral therapy for prevention of HIV transmission in HIV-discordant couples. Cochrane Database Syst Rev. 2013 Apr 30;(4).

41. Mastro TD, de Vincenzi I.Probabilities of sexual HIV-1 transmission. AIDS.1996.10 Suppl A:S75-82.

42. Gray RH, Wawer MJ, Brookmeyer R, Sewankambo NK, Serwadda D, Wabwire-Mangen F, et al. Probability of HIV-1 transmission per coital act in monogamous, heterosexual, HIV-1-discordant couples in Rakai, Uganda. Lancet. 2001 April 14; 357(9263):1149-53.

43. Young J, Rickenbach M, Calmy A, Bernasconi E, Staehelin C, Schmid P, et al. Transient detectable viremia and the risk of viral rebound in patients from the Swiss HIV Cohort Study. BMC Infect Dis. 2015; 15(1):382.

44. Sörstedt E, Nilsson S, Blaxhult A, Gisslén M, Flamholc L, Sönnerborg A,et al. Viral blips during suppressive antiretroviral treatment are associated with high baseline HIV-1 RNA levels. BMC Infectious Diseases. 2016; 16:305.

45. Van Sighem A, Zhang S, Reiss P, Gras L, van der Ende M, Kroon F, et al. Immunologic, virologic, and clinical consequences of episodes of transient viremia during suppressive combination antiretroviral therapy. J Acquir Immune Defic Syndr. 2008 May 1; 48:104-8.

46. Teira R, Vidal F, Muñoz-Sánchez P, Geijo P, Viciana P, Ribera E, et al. Very low level viraemia and risk of virological failure in treated HIV-1-infected patients. HIV Medicine, online edition. HIV Med. 2017 Mar; 18(3):196-203.

47. Fonner V, Dalglish S, Kennedya C, Baggaley R, O’Reilly K, Koechlinb F, et al. Effectiveness and safety of oral HIV preexposure prophylaxis for all populations. AIDS. 2016; 30:1973-83.

48. Molina J-M, Capitant C, Spire B, Pialoux G, Cotte L, Charreau I, et al. On-Demand Preexposure Prophylaxis in Men at High Risk for HIV-1 Infection. N Engl J Med. 2015 Dec 3; 373:2237-46.

49. McCormack, S, Dunn D, Desai M, Dolling D, Gafos M, Gilson R, et al. Pre-exposure prophylaxis to prevent the acquisition of HIV-1 infection (PROUD): effectiveness results from the pilot phase of a pragmatic open-label randomised trial. The Lancet. 2015 Sept 9; 387(10013):53-60.

50. Grant RM, Liegler T, Defechereux P, Kashuba AD, Taylor D, Abdel-Mohsen M, et al. Drug resistance and plasma viral RNA level after ineffective use of oral pre-exposure prophylaxis in women. AIDS. 2015; 29:331-7.

51. Schechter M, do Lago R, Mendelsohn A, Moreira R, Moulton L, Harrison L, et alBehavioral impact, acceptability, and HIV incidence among homosexual men with access to postexposure chemoprophylaxis for HIV. JAIDS. 2004 15 April; 35(5):519-25.

52. Pinkerton SD, Martin J, Roland M, Katz M, Coates T, Kahn J, et al. Cost-effectiveness of postexposure prophylaxis after sexual or injection-drug use exposure to human immunodeficiency virus. Arch Intern Med. 2004; 164:46-54.

53. Jochimsen EM. Failures of zidovudine postexposure prophylaxis. Am J Med. 1997 May 19;102(5) Sup2:52-55.

54. Lot F, Abiteboul D. Occupational infections with HIV in France among health-care personnel. Bull Epi Hebdom. 1999; 18:69-70. На французском языке.

55. Beltrami EM, Luo C-C, de la Torre N, Cardo DM. Transmission of drug-resistant HIV after an occupational exposure despite postexposure prophylaxis with a combination drug regimen. Infect Control Hosp Epidemiols. 2002; 23:345-48, 2002.

56. Hawkins DA, Asboe D, Barlow K, Evans B. Seroconversion to HIV-1 following a needlestick injury despite combination post-exposure prophylaxis. J Infect. 2001; 43:12-15.

57. Wulfsohn A, Venter WDF, Schultze D, Levey M, Sanne IM. Post-exposure prophylaxis after sexual assault in South Africa. Proceedings of the Tenth Conference on Retroviruses and Opportunistic Infections; 2003 Feb; Boston, U.S.: abstract 42.

58. Lunding S et al. Danish postexposure prophylaxis (PEP) registry: use and failure of antiretroviral chemoprophylaxis following sexual exposure to HIV. Sixteenth International AIDS Conference, Toronto, abstract TUPE0433, 2006.

59. Donnell D, Mimiaga MJ, Mayer K, Chesney M, Koblin B, Coates T. Use of non-occupational post-exposure prophylaxis does not lead to an increase in high risk sex behaviors in men who have sex with men participating in the EXPLORE trial. AIDS Behav. 2010; 14(5):1182-1189.

60. Sonder GJB, Prins JM, Regez RM, et al. Comparison of two HIV postexposure prophylaxis regimens among men who have sex with men in Amsterdam: adverse effects do not influence compliance. Sex Transm Dis. 2010; 37(11):681-686.

61. McAllister J, Read P, McNulty A, Tong WW, Ingersoll A, Carr A. Raltegravir-emtricitabine-tenofovir as HIV nonoccupational post-exposure prophylaxis in men who have sex with men: safety, tolerability and adherence. HIV Med. 2014; 15(1):13-22.

62. Jain S, Oldenburg CE, Mimiaga MJ, Mayer KH. Subsequent HIV infection among men who have sex with men who used non-occupational post-exposure prophylaxis at a Boston community health center: 1997-2013. AIDS Patient Care STDS. 2015; 29(1):20-25.

63. Foster R, McAllister J, Read TR, et al. Single-tablet emtricitabine-rilpivirine-tenofovir as HIV postexposure prophylaxis in men who have sex with men. Clin Infect Dis. 2015:1-5.

64. Linden JA, Oldeg P, Mehta SD, McCabe KK, LaBelle C. HIV postexposure prophylaxis in sexual assault: current practice and patient adherence to treatment recommendations in a large urban teaching hospital. Acad Emerg Med. 2005; 12(7):640-646.

65. Griffith WF, Ackerman GE, Zoellner CL, Sheffield JS. Sexual assault: a report on human immunodeficiency virus postexposure prophylaxis. Obstet Gynecol Int. 2010;(196963):1-6.

66. Olshen E, Hsu K, Woods ER, Harper M, Harnisch B, Samples CL. Use of human immunodeficiency virus postexposure prophylaxis in adolescent sexual assault victims. Arch of Pediat Adolesc Med. 2006; 160(7):674-680.

67. Cardo D, Culver D, Ciesielski C, Srivastava P, Marcus R, Abiteboul D, et al. A Case–Control Study of HIV Seroconversion in Health Care Workers after Percutaneous Exposure. N Engl J Med. 1997 Nov 20; 337:1485-90.

68. Poynten IM, Smith DE, Cooper DA, Kaldor JM, Grulich AE. The public health impact of widespread availability of nonoccupational postexposure prophylaxis against HIV. HIV Medicine. 2007 September; 8(6):374-381.

69. Siegfried N, Muller M, Deeks JJ, Volmink J. Male circumcision for prevention of heterosexual acquisition of HIV in men. Cochrane Database Syst Rev. 2009 Apr 15;(2): CD003362.

70. Millett GA, Flores SA, Marks G, Reed JB, Herbst JH. Circumcision status and risk of HIV and sexually transmitted infections among men who have sex with men: a meta-analysis. JAMA. 2008 Oct 8; 300(14):1674-84. Erratum in: JAMA. 2009 Mar 18; 301(11):1126-9.

71. Crepaz N, Marks G, Liau A, Mullins MM, Aupont LW, Marshall KJ et al. Prevalence of unprotected anal intercourse among HIV-diagnosed MSM in the United States: a meta-analysis. AIDS. 2009 Aug 24; 23(13):1617-29.

72. Van De Ven P, Kippax S, Crawford J, Rawstorne P, Prestage G, Grulich A et al. In a minority of gay men, sexual risk practice indicates strategic positioning for perceived risk reduction rather than unbridled sex. AIDS Care. 2002 Aug; 14(4):471-80.

73. Jin F, Crawford J, Prestage G,Zablotska I,b Imrie J, Kippax S, et al. Unprotected anal intercourse, risk reduction behaviours, and subsequent HIV infection in a cohort of homosexual men. AIDS. 2009 January 14; 23(2):243–52.

74. Baggaley RF, Boily M-C, White RG, Alary M. Risk of HIV-1 transmission for parenteral exposure and blood transfusion: a systematic review and meta-analysis. AIDS. 2006; 20:805-12.

75. Vittinghoff E, Douglas J, Judson F, McKirnan D, MacQueen K, Buchbinder SP. Per-contact risk of human immunodeficiency virus transmission between male sexual partners. Am J Epidemiol.1999 Aug 1;150:306-11.

76. Sexton J, Garnett G, Røttingen J-A. Metaanalysis and metaregression in interpreting study variability in the impact of sexually transmitted diseases on susceptibility to HIV infection. Sex Transm Dis. 2005 Jun; 32(6):351-7.

77. Campo J, Perea MA, del Romero J, Cano J, Hernando V, Bascones A. Oral transmission of HIV, reality or fiction? An update. Oral Dis. 2006 May; 12(3):219-28.

78. Baggaley RF, White RG, Boily MC. Systematic review of orogenital HIV 1 transmission probabilities. Int J Epidemiol.2008 Dec;37(6):1255-65.

79. Morrow G, Vachot L, Vagenas P and Robbiani M. Current concepts of HIV transmission. Curr HIV/AIDS Rep. 2007 Feb; 4(1):29-35.

80. Centers for Disease Control and Prevention. Oral sex and HIV Risk. CDC HIV/AIDS Facts, June 2009.

81. del Romero J, Marincovich B, Castilla J, Garcia S, Campo J, Hernando V,.et al. Evaluating the risk of HIV transmission through unprotected orogenital sex. AIDS 2002 June 14; 16(9):1296–97.

82. Raiteri R, Fora R, Sinicco A. No HIV-1 transmission through lesbian sex. Lancet 1994;344:270.

83. European Study Group on Heterosexual Transmission of HIV. Comparison of female to male and male to female transmission of HIV in 563 stable couples. BMJ 1992 Mar 28; 304(6830):809-13.

84. Boily M-C, Baggaley RF, Wang L, Masse B, White RG, Hayes RJ, et al. Heterosexual risk of HIV-1 infection per sexual act: systematic review and meta-analysis of observational studies. Lancet Infect Dis 2009 Feb;9(2):118-29.

85. Rodger A, Cambiano V, Bruun T, Vernazza P, Collins S, Estrada V, et al. HIV transmission risk through condomless sex if the HIV positive partner is on suppressive ART: PARTNER study. Presentation at CROI, 2014 Mar 3-6;Boston, U.S.

86. Baggaley RF, White RG, Boily M-C. HIV transmission risk through anal intercourse: systematic review, meta-analysis and implications for HIV prevention. Int J Epidemiol. 2010 Aug; 39(4):1048-63.

87. Halperin DT, Shiboski SC, Palefsky JM and Padian NS. High level of HIV-1 infection from anal intercourse: a neglected risk factor in heterosexual AIDS prevention. Paper presented at XIV International AIDS Conference. 2002; Barcelona, Spain.

88. Leynaert B, Downs AM, de Vincenzi I. Heterosexual transmission of human immunodeficiency virus: variability of infectivity throughout the course of infection. European Study Group on Heterosexual Transmission of HIV. Am J Epidemiol. 1998 Jul 1; 148(1):88-96

89. Jin F, Jansson J, Law M, Prestage GP, Zablotska I, Imrie JC, et al. Per-contact probability of HIV transmission in homosexual men in Sydney in the era of HAART. AIDS. 2010 Mar 27; 24(6):907-13.

90. DeGruttola V, Seage GR 3rd, Mayer KH and Horsburgh CR Jr. Infectiousness of HIV between male homosexual partners. J Clin Epidemiol.1989;42(9):849-56.

91. Jacquez JA, Koopman JS, Simon CP and Longini IM Jr. Role of the primary infection in epidemics of HIV infection in gay cohorts. J Acquir Immune Defic Syndr. 1994 Nov; 7(11):1169-84.

92. Bavinton B, B. Grinsztejn, N. Phanuphak, F. Jin, I. Zablotska, G. Prestage, et al. HIV treatment prevents HIV transmission in male serodiscordant couples in Australia, Thailand and Brazil. 9th International AIDS Society Conference on HIV Science, Paris, abstract no TUAC0506LB, July 2017.

93. Berthier A, Fauchet R, Genetet N, Fonlupt J, Genetet N, Gueguen M, et al. Transmissibility of human immunodeficiency virus in haemophilic an non-haemophilic children living in a private school in France. Lancet 1986 Sep 13; 2(8507):598-601.

94. Fischl MA, Dickinson GM, Scott GE, et al. Evaluation of heterosexual partners, children and household contacts of adults with AIDS. JAMA 1987; 257:640-4.

95. Friedland G, Kahl P, Saltzman B, Rogers M, Feiner C, Mayers M, et al. Additional evidence for lack of transmission of HIV infection by close interpersonal (casual) contact. AIDS. 1990 Jul 1; 4(7):639-44.

96. Rogers MF, White CR, Sanders R, Schable C, Ksell TE, Wasserman RL, et al. Lack of transmission of human immunodeficiency virus from infected children to their household contacts. Pediatrics. 1990 Feb; 85(2):210-14.

97. Courville TM, Caldwell B, Brunell P. Lack of Evidence of Transmission of HIV-1 to Family Contacts of H1V-1 Infected Children. Clinical Pediatrics. 1998 Mar; 37(3):175-8.

98. Lusher JM, Operskalski EA, Lee H, Mosley JW, Aledort LM, Dietrich, SL, et al. Risk of human immunodeficiency virus type 1 infection among sexual and nonsexual household contacts of persons with congenital clotting disorders. Pediatrics. 1991 Aug; 88(2):242-9.

99. Gilbart VL. Unusual HIV transmissions through blood contact: analysis of cases reported in the United Kingdom to December 1997. Communicable Disease and Public Health. 1998 June;1(2): 108-13.

100. Yeung SC, Kazazi F, Randle CG, et al. Patients infected with human immunodeficiency virus type 1 have low levels of virus in saliva even in the presence of periodontal disease. J Infect Dis. 1993 Apr; 167(4):803-9.

101. Shine N, Konopka K, Düzgüneş N: The anti-HIV-1 activity associated with saliva. J Dent Res. 1997 Feb; 76(2):634-640.

102. Shugars DC, Schock DC, Patton J. HIV-1 RNA load in blood plasma, saliva and crevicular fluid. J Dent Res. 1998; 77(Special Issue A):285.

103. Shugars DC, Wahl S. The role of the oral environment in HIV-1 transmission. JADA. 1998 July; 129(7):851-8.

104. Malamud D, Friedman HM. HIV in the oral cavity: virus, viral inhibitory activity, and antiviral antibodies. Crit Rev Oral Biol Med. 1993; 4(3-4):461–6.

105. Archibald DW, Cole GA. In vitro inhibition of HIV-1 infectivity by human salivas. AIDS Res Hum Retroviruses 1990 Dec; 6(12):1425–32.

106. Yeh CK, Handelman B, Fox PC, Baum BJ. Further studies of salivary inhibition of HIV-1 infectivity. J Acquir Immune Defic Syndr. 1992; 5(9):898-903.

107. Cresswell FV, Ellis J, Hartley J, Sabin CA, Orkin C, Churchill DR. A systematic review of risk of HIV transmission through biting or spitting: implications for policy. HIV Medicine. 2018 April 23. Доступно на https://onlinelibrary.wiley.com/doi/abs/10.1111/hiv.12625

108. Tsoukas C et al. Lack of transmission of HIV through human bites and scratches. JAIDS 1988; 1(5):505-7.

109. Richman KM, Richman LS. The potential for transmission of human immunodeficiency virus through human bites. J Acquir Immune Defic Syndr. 1993; 64:40-46.

110. Shirley LR, Ross SA, Risk of transmission of human immunodeficiency virus by bite of an infected toddler. The Journal of Pediatrics. 1989 March; 114(3):425-7.

111. Drummond R. Seronegative 18 months after being bitten by a patient with AIDS. JAMA. 1986 Nov 7; 256(17):2342-3.

112. Romea S, Alkiza ME, Ramon JM, Oromí J. Risk for occupational transmission of HIV infection among health care workers. European Journal of Epidemiology. 1995 April; 11(2):225–9.

113. Verrusio AC, Risk of transmission of the human immunodeficiency virus to health care workers exposed to HIV-infected patients: a review, JADS. 1989 March; 118(3):339-42.

114. Henderson DK, Fahey BJ, Willy M, Schmitt JM, Carey K, Koziol DE, et al. Risk for Occupational Transmission of Human Immunodeficiency Virus Type 1 (HIV-1) Associated with Clinical Exposures: A Prospective Evaluation. Ann Intern Med. 1990 Nov 15; 113(10):740-746.

115. Tereskerz PM, Bentley M, Jagger J. Risk of HIV-1 infection after human bites. Lancet. 1996 Nov 30; 348(9040):1512.

116. Sabin CA, Lundgren JD. The natural history of HIV infection. Current Opinion in HIV and AIDS. 2013; 8(4):311-317.

117. Okulicz JF, Marconi VC, Landrum ML, Wegner S, Weintrob A, Ganesan A, et al. Clinical Outcomes of Elite Controllers, Viremic Controllers, and Long-Term Nonprogressors in the US Department of Defense HIV Natural History Study. J Infect Dis. 2009 Dec 1; 200(11): 1714-23.

118. McManus H, O'Connor CC, Boyd M, Broom J, Russell D, Watson K, et al. Long-term survival in HIV positive patients with up to 15 Years of antiretroviral therapy. PLoS One. 2012 Nov 7; 7(11).

119. Lee FJ, Amin J, Carr A. Efficacy of initial antiretroviral therapy for HIV-1 infection in adults: a systematic review and meta-analysis of 114 studies with up to 144 weeks' follow-up. PLoS One. 2014 May 15; 9(5):e97482.

120. The INSIGHT START Study Group. Initiation of Antiretroviral Therapy in Early Asymptomatic HIV Infection. N Engl J Med. 2015 Aug 27; 373(9): 795-807.

121. Stephan C, Hill A, Sawyer W, van Delft Y, Moecklinghoff C. Impact of baseline HIV-1 RNA levels on initial highly active antiretroviral therapy outcome: a meta-analysis of 12,370 patients in 21 clinical trials. HIV Med. 2013 May; 14(5):284-92.

122. Trickey A, May M, Vehreschild J-J, Obel N, Gill MJ, Crane H, et al. Survival of HIV-positive patients starting antiretroviral therapy between 1996 and 2013: a collaborative analysis of cohort studies. The Lancet HIV. 2017 August; 4(8):e349-56.

123. Patterson S, Cescon A, Samji H, Chan K, Zhang W, Raboud J, et al. Life expectancy of HIV-positive individuals on combination antiretroviral therapy in Canada. BMC Infect Dis. 2015 Jul 17; 15:274.

124. Zhu H, Napravnik S, Eron JJ, Cole SR, Ma Y, Wohl DA, et al. Decreasing excess mortality of HIV-infected patients initiating antiretroviral therapy: comparison with mortality in general population in China, 2003-2009. J Acquir Immune Defic Syndr. 2013 Aug 15; 63(5):e150-7.

125. Teeraananchai S, Kerr SJ, Amin J, Ruxrungtham K, Law MG. Life expectancy of HIV-positive people after starting combination antiretroviral therapy: a meta-analysis. HIV Med. 2017 Apr; 18(4):256-66.

126. Lohse N, Obel N. Update of Survival for Persons with HIV Infection in Denmark. Ann Intern Med. 2016 Nov 15; 165(10):749-750.

127. Price AJ, Glynn J, Chihana M, Kayuni N, Floyd S, Slaymaker E, et al. Sustained 10-year gain in adult life expectancy following antiretroviral therapy roll-out in rural Malawi: July 2005 to June 2014. Int J Epidemiol. 2017; 46(2):479-91.

128. Nsanzimana S, Remera E, Kanters S, Chan K, Forrest JI, Ford N, et al. Life expectancy among HIV-positive patients in Rwanda: a retrospective observational cohort study. Lancet Glob Health. 2015 Mar; 3(3):e169-77.

129. Johnson LF, Mossong J, Dorrington RE, Schomaker M, Hoffmann CJ, Keiser O, et al. International Epidemiologic Databases to Evaluate AIDS Southern Africa Collaboration. Life expectancies of South African adults starting antiretroviral treatment: collaborative analysis of cohort studies. PLoS Med. 2013 April 9; 10(4):e1001418.

130. Reniers G, Blom S, Calvert C, Martin-Onraet A, Herbst AJ, Eaton JW, et al. Trends in the burden of HIV mortality after roll-out of antiretroviral therapy in KwaZulu-Natal, South Africa: an observational community cohort study. Lancet HIV. 2017 Mar; 4(3):e113-e121.

131. Gueler A, Moser A, Calmy A, Günthard HF, Bernasconi E, Furrer H, et al. Life expectancy in HIV-positive persons in Switzerland: matched comparison with general population. AIDS. 2017 Jan 28; 31(3):427-36.

132. Teeraananchai S, Chaivooth S, Kerr SJ, Bhakeecheep S, Avihingsanon A, Teeraratkul A, Sirinirund P, Law MG, Ruxrungtham K. Life expectancy after initiation of combination antiretroviral therapy in Thailand. Antivir Ther. 2017 Jan 5.

133. Asiki G, Reniers G, Newton R, Baisley K, Nakiyingi-Miiro J, Slaymaker E, et al. Adult life expectancy trends in the era of antiretroviral treatment in rural Uganda (1991-2012). AIDS. 2016 Jan 28; 30(3):487-93.

134. May MT, Gompels M, Delpech V, Porter K, Orkin C, Kegg S, et al. Impact on life expectancy of HIV-1 positive individuals of CD4+ cell count and viral load response to antiretroviral therapy. AIDS. 2014 May 15; 28(8):1193-202.

135. Samji H, Cescon A, Hogg RS, Modur SP, Althoff KN, Buchacz K, et al. Closing the gap: increases in life expectancy among treated HIV-positive individuals in the United States and Canada. PLoS One. 2013 Dec 18; 8(12):e81355.

136. Siddiqi AE, Hall HI, Hu X, Song R. Population-Based Estimates of Life Expectancy After HIV Diagnosis: United States 2008-2011. J Acquir Immune Defic Syndr. 2016 Jun 1; 72(2):230-6.

137. Marcus JL, Chao CR, Leyden WA, Xu L, Quesenberry CP Jr, Klein DB, et al. Narrowing the Gap in Life Expectancy Between HIV-Infected and HIV-Uninfected Individuals With Access to Care. J Acquir Immune Defic Syndr. 2016 Sep 1; 73(1):39-46.

138. Furuya-Kanamori L, Kelly MD, McKenzie SJ. Co-morbidity, ageing and predicted mortality in antiretroviral treated Australian men: a quantitative analysis. PLoS One. 2013 Oct 25; 8(10):e78403.

139. van Sighem AI, Gras LA, Reiss P, Brinkman K, de Wolf F. Life expectancy of recently diagnosed asymptomatic HIV-infected patients approaches that of uninfected individuals. AIDS. 2010 Jun 19; 24(10):1527-35.

140. Nglazi M, West S, Dave J, Levitt N, Lambert E. Quality of life in individuals living with HIV/AIDS attending a public sector antiretroviral service in Cape Town, South Africa. BMC Public Health. 2014 July 3; 14:676.

141. Wandeler G, Johnson LF, Egger M. Trends in life expectancy of HIV-positive adults on antiretroviral therapy across the globe: comparisons with general population. Curr Opin HIV AIDS. 2016 Sep; 11(5):492-500.

142. Palella FJ Jr, Baker RK, Moorman AC, Chmiel JS, Wood KC, Brooks JT, et al. Mortality in the highly active antiretroviral therapy era: changing causes of death and disease in the HIV outpatient study. J Acquir Immune Defic Syndr. 2006 Sep; 43(1):27-34.

143. Crum NF, Riffenburgh RH, Wegner S, Agan BK, Tasker SA, Spooner KM, et al. Comparisons of causes of death and mortality rates among HIV-infected persons: analysis of the pre-, early, and late HAART (highly active antiretroviral therapy) eras. J Acquir Immune Defic Syndr. 2006 Feb 1; 41(2):194-200.

144. Deeks SG, Phillips AN. HIV infection, antiretroviral treatment, ageing, and non-AIDS related morbidity. BMJ. 2009 Jan 31; 338:288-92.

145. Kojic EM, Wang CC, Cu-Uvin S. (2007). HIV and menopause: a review. J Womens Health (Larchmt). Dec; 16(10):1402-11.

146. Manfredi R. HIV disease and advanced age: an increasing therapeutic challenge. Drugs Aging. 2002; 19(9):647-69.

147. Manfredi R. HIV infection and advanced age emerging epidemiological, clinical, and management issues. Ageing Res Rev. 2004;Jan;3(1):31-54.

148. Serrano-Villar S, Gutiérrez F, Miralles C, Berenguer J, Rivero A, Martínez E, et al. Human Immunodeficiency Virus as a Chronic Disease: Evaluation and Management of Nonacquired Immune Deficiency Syndrome-Defining Conditions. Open Forum Infect Dis. 2016 May 12; 3(2):ofw097.

149. Narayan KM, Miotti PG, Anand NP, Kline LM, Harmston C, Gulakowski R 3rd, et al. HIV and noncommunicable disease comorbidities in the era of antiretroviral therapy: a vital agenda for research in low- and middle-income country settings. J Acquir Immune Defic Syndr. 2014 Sep 1; 67 Suppl 1:S2-7.

150. Bloomfield GS, Khazanie P, Morris A, Rabadán-Diehl C, Benjamin LA, Murdoch D, et al. HIV and noncommunicable cardiovascular and pulmonary diseases in low- and middle-income countries in the ART era: what we know and best directions for future research. J Acquir Immune Defic Syndr. 2014 Sep 1; 67 Suppl 1:S40-53.

151. Helleberg M, Afzal S, Kronborg G, Larsen CS, Pedersen G, Pedersen C, et al.Mortality Attributable to Smoking Among HIV-1-Infected Individuals: A Nationwide, Population-Based Cohort Study. Clin Infect Dis. 2013 Mar; 56(5):727-34.

152. Rodriguez B, Sethi AK, Cheruvu VK, Mackay W, Bosch RJ, Kitahata M, et al. Predictive value of plasma HIV RNA level on rate of CD4 T-cell decline in untreated HIV infection. Journal of the American Medical Association, 2006 Sep 27;296(12):1498-1506.

153. Bernard E, Azad Y, Delpech V, Geretti AM. HIV Forensics II: Estimating the likelihood of recent HIV infection: Implications for criminal prosecution. National AIDS Trust. London. July 2011.

154. Goodenow M, Huet T, Saurin W, Kwok S, Sninsky J, Wain-Hobson S. HIV-1 isolates are rapidly evolving quasispecies: evidence for viral mixtures and preferred nucleotide substitutions. J Acquir Immune Defic Syndr 1989; 2:344–352.

155. Abecasis A, Pingarilho M, Vandamme A-M. Phylogenetic analysis as a forensic tool in HIV transmission investigations: a literature review. AIDS 2017 Dec 26; 31, 2017.

156. Romero-Severson E, Skar H, Bulla I, Albert J, Leitner T. Timing and order of transmission events is not directly reflected in a pathogen phylogeny. Mol Biol Evol 2014; 31:2472–2482.

157. Rambaut A, Posada D, Crandall KA, Holmes EC. The causes and consequences of HIV evolution. Nat Rev Genet 2004; 5:52–61.

158. Bernard EJ, Azad Y, Vandamme AM, Weait M, Geretti AM. HIV forensics: pitfalls and acceptable standards in the use of phylogenetic analysis as evidence in criminal investigations of HIV transmission. HIV Med. 2007 Sep; 8(6):382–387.

159. Romero-Severson EO, Bulla I, Leitner T. Phylogenetically resolving epidemiologic linkage. Proc Natl Acad Sci USA. 2016 Mar 8; 113(10):2690-5.

160. Huelsenbeck J, Hillis DM. Success of phylogenetic methods in the four-taxon case. Syst Biol 1993;247–264.

161. Wertheim JO, Sanderson MJ, Worobey M, Bjork A. Relaxed molecular clocks, the bias-variance trade-off, and the quality of phylogenetic inference. Syst Biol 2010; 59:1–8.

162. González-Candelas F, Bracho MA, Wróbel B, Moya A. Molecular evolution in court: analysis of a large hepatitis C virus outbreak from an evolving source. BMC Biol 2013; 11:76.

163. Abecasis AB, Geretti AM, Albert J, Power L, Weait M, Vandamme A-M. Science in court: the myth of HIV fingerprinting. Lancet Infect Dis. 2011 Feb; 11(2):78-9.).

164. Ou CY, Ciesielski CA, Myers G, Bandea CI, Luo CC, Korber BT, et al. Molecular epidemiology of HIV transmission in a dental practice. Science 1992; 256:1165–1171.

165. Smith TF, Waterman MS. The continuing case of the Florida dentist. Science 1992; 256:1155-1156.

166. DeBry RW, Abele LG, Weiss SH, Hill MD, Bouzas M, Lorenzo E, et al. Dental HIV transmission? Nature 1993; 361:691.

167. Lemey P, Van Dooren S, Van Laethem K, Schrooten Y, Derdelinckx I, Goubau P, et al. Molecular testing of multiple HIV-1 transmissions in a criminal case. AIDS Lond Engl 2005; 19:1649–1658.

168. Goedhals D, Rossouw I, Hallbauer U, Mamabolo M, de Oliveira T. The tainted milk of human kindness. Lancet Lond Engl 2012; 380:702.

169. Paraschiv S, Banica L, Nicolae I, Niculescu I, Abagiu A, Jipa R, et al. Epidemic dispersion of HIV and HCV in a population of co-infected Romanian injecting drug users. PloS One 2017; 12.
