# Supplementary material for: Expert consensus statement on the science of HIV in the context of criminal law
Source: J Int AIDS Soc. 2018 Jul 25;21(7):e25161. doi: 10.1002/jia2.25161 (PMC6058263; doi:10.1002/jia2.25161)
Supplement: Supplementary file 5 — Supplementary Material S5. Expert Consensus Statement SPANISH Translation. [file JIA2-21-e25161-s005.pdf]

Declaración de Consenso de expertos sobre la ciencia relativa al VIH en el contexto del derecho penal

Françoise Barré-Sinoussi[1], Salim S. Abdool Karim[2,3, 4], Jan Albert[5], Linda-Gail Bekker[6], Chris Beyrer[7], Pedro Cahn[8, 9, 10], Alexandra Calmy[11], Beatriz Grinsztejn[12], Andrew Grulich[13], Adeeba Kamarulzaman[14], Nagalingeswaran Kumarasamy[15], Mona Rafik Loutfy[16, 17, 18], Kamal Marhoum El Filali[19], Souleymane Mboup[20], Julio S.G Montaner[21,22], Paula Munderi[23], Vadim Pokrovsky[24,25], Anne-Mieke Vandamme[26, 27], Benjamin Young[28] y Peter Godfrey-Faussett[§29,30]

1. Instituto Pasteur, París (Francia)
2. Facultad de Salud Pública Mailman de la Universidad de Columbia, Nueva York (Estados Unidos de América)
3. Centro para el Programa de Investigación del Sida en Sudáfrica de la Universidad de KwaZulu-Natal, Durban (Sudáfrica)
4. Facultad de Medicina Weill de la Universidad Cornell, Nueva York (Estados Unidos de América)
5. Departamento de Microbiología y Biología Tumoral y Celular del Instituto Karolinska, Estocolmo (Suecia)
6. Instituto de Enfermedades Infecciosas y Medicina Molecular de la Universidad de Ciudad del Cabo, Ciudad del Cabo (Sudáfrica)
7. Departamento de Epidemiología del Centro para la Investigación del Sida y el Centro para la Salud Pública y los Derechos Humanos de la Facultad de Salud Pública John Hopkins Bloomberg, Maryland (Estados Unidos)
8. División de Infectología del Hospital Juan A. Fernández, Buenos Aires (Argentina)
9. Facultad de Medicina de la Universidad de Buenos Aires, Buenos Aires (Argentina)
10. Fundación Huésped, Buenos Aires (Argentina)
11. Servicio de Enfermedades Infecciosas del Hospital Universitario de Ginebra, Ginebra (Suiza)
12. Instituto Nacional de Infectología Evandro Chagas-Fiocruz de la Fundación Oswaldo Cruz, Río de Janeiro (Brasil)
13. Instituto Kirby de la Universidad de Nueva Gales del Sur, Sídney (Australia)
14. Facultad de Medicina de la Universidad de Malaya, Kuala Lumpur (Malasia)
15. Centro Médico YRGCARE, Voluntary Health Services, Chennai (India)
16. Instituto de Investigación del Women's College Hospital, Toronto (Canadá)
17. Women's College Hospital, Toronto (Canadá)
18. Departamento de Medicina de la Universidad de Toronto (Canadá)
19. Servicio de Enfermedades Infecciosas del Hospital Universitario Ibn Rochd, Casablanca (Marruecos)
20. Instituto de Investigación para la Salud, la Vigilancia Epidemiológica y la Formación, Dakar (Senegal)
21. Facultad de Medicina de la Universidad de Columbia Británica, Vancouver (Canadá)
22. Centro de Excelencia para el VIH/Sida de Columbia Británica, Vancouver (Canadá)
23. Asociación Internacional de Proveedores de Atención al Sida, Kampala (Uganda)
24. Universidad Rusa de la Amistad de los Pueblos (RUDN), Moscú (Federación de Rusia)
25. Instituto de Investigación Central sobre Epidemiología del Servicio Federal para la Protección de los Derechos de los Consumidores y la Vigilancia del Bienestar Humano, Moscú (Federación de Rusia)
26. Universidad Católica de Lovaina, Departamento de Microbiología e Inmunología del Instituto de Investigación Médica Rega, Virología Clínica y Epidemiológica, Lovaina (Bélgica)

27. Unidad de Microbiología del Instituto de Higiene y Medicina Tropical de la Universidad Nueva de Lisboa, Lisboa (Portugal)
28. Asociación Internacional de Proveedores de Atención al Sida, Washington D.C. (Estados Unidos)
29. ONUSIDA, Ginebra (Suiza)
30. Departamento de Enfermedades Infecciosas y Tropicales de la Escuela de Higiene y Medicina Tropical de Londres, Londres (Inglaterra)

[§] **Autor encargado de la correspondencia:** Prof. Peter Godfrey-Faussett

Dirección: ONUSIDA, Avenue Appia 20, 1211 Genève, Suiza

Teléfono: + 41 22 791 4054

Correo electrónico: godfreyp@unaids.org

Direcciones de correo electrónico de los autores:

F. B.-S.: francoise.barre-sinoussi@pasteur.fr

J. A.: Jan.Albert@ki.se

S. S. A. K.: Salim.AbdoolKarim@caprisa.org

L.-G. B.: linda-gail.bekker@hiv-research.org.za

C. B.: cbeyrer@jhu.edu

P. C.: pedro.cahn@huesped.org.ar

A. C.: alexandra.calmy@hcuge.ch

P. G.-F. : faussettp@unaids.org

B. G.: beatriz.grinsztejn@gmail.com

A. G.: agrulich@kirby.unsw.edu.au

A. K.: adeeba@ummc.edu.my

N. M.: kumarasamy@yrgcare.org

M. R. L.: mona.loutfy@wchospital.ca

K. M. E. F.: mefkamal@hotmail.com

S. M.: souleymane.mboup@iressef.org

J. S. G. M.: jmontaner@cfenet.ubc.ca

P. M.: munderip@gmail.com

V. P.: pokrovsky.vad@yandex.ru

A.-M. V.: annemie.vandamme@kuleuven.be

B. Y.: byoung@iapac.org

Palabras clave

- derechos humanos
- derecho y políticas
- factores de riesgo
- políticas
- penalización
- tipificación
- derecho penal
- procesamiento

Resumen

Introducción

A nivel mundial, los procesamientos por la no divulgación del estado serológico respecto del VIH, exposición al VIH o transmisión del VIH a menudo están vinculados a actividades sexuales, mordeduras o escupidas. Esto incluye instancias donde no hubo intención de causar ningún daño, no se produjo la transmisión del VIH o dicha transmisión era imposible o muy poco probable. Esto sugiere que los procesamientos no siempre se basan en las mejores pruebas científicas y médicas disponibles.

Discusión

Veinte científicos provenientes de diferentes regiones del mundo elaboraron la presente Declaración de Consenso de expertos con el objeto de abordar la utilización que el sistema de justicia penal hace de la ciencia relativa al VIH. Para esto, se llevó a cabo un análisis exhaustivo de la información publicada en investigaciones científicas y médicas sobre la transmisión del VIH, la eficacia de su tratamiento y las pruebas filogenéticas forenses, y se adaptó a un lenguaje más comprensible para el contexto del derecho penal.

La descripción de las posibilidades de transmisión del VIH se limitó a las vías más frecuentes discutidas en las causas penales. La probabilidad de transmisión del VIH en un evento único y aislado se analizó en el contexto de un de riesgo continuo, dado que las probabilidades de que el VIH se transmita varían en función de diversos factores interrelacionados, como la carga viral, la utilización de preservativo y otras prácticas que reducen el riesgo de transmisión. Las pruebas actuales sugieren que las posibilidades de que el VIH se transmita durante un único acto sexual, mordedura o escupida son inexistentes o escasas.

Se investigó el efecto positivo de las terapias antirretrovirales en la salud de las personas viviendo con VIH, las cuales han aumentado su esperanza de vida, siendo ahora similar a la de las personas VIH-negativas, por lo que, la infección por el VIH se ha transformado en una condición crónica tratable. Por último, el estudio de la utilización de pruebas científicas en los tribunales reveló que los análisis filogenéticos por sí solos no pueden demostrar más allá de toda duda razonable que una persona haya infectado a otra, aunque se pueden utilizar para eximir de responsabilidad al encausado.

Conclusiones

La utilización de las últimas pruebas científicas sobre la ciencia del VIH en causas penales puede reducir el número de procesamientos y fallos condenatorios injustos. Los autores recomiendan obrar con prudencia durante los procesamientos, al mismo tiempo que recomiendan a los Gobiernos y aquellos actores que trabajan en los sistemas jurídicos y judiciales a prestar especial atención a los avances significativos que se han producido a lo largo de los tres últimos decenios en la esfera científica del VIH para que la aplicación de las leyes en los casos relacionados a la transmisión del VIH se fundamente en los últimos conocimientos científicos.

Introducción

Al menos 68 países cuentan con leyes que penalizan específicamente la no divulgación del estado serológico respecto del VIH, la exposición al VIH o la transmisión del VIH. Se tiene conocimiento de que 33 países han aplicado otras disposiciones del derecho penal en casos similares. (datos inéditos, HIV Justice Network, 2018). La mayoría de los procesamientos han estado vinculados al riesgo percibido de contraer el VIH asociado a la actividad sexual, aunque también se han registrado procesamientos por actos como morder o escupir (datos inéditos, HIV Justice Network, 2018). Este tipo de leyes y procesamientos no siempre se han basado en las pruebas científicas y médicas disponibles sobre la transmisión del VIH [1], no han evolucionado para reflejar los avances de los conocimientos sobre el VIH y su tratamiento, y pueden estar influenciados por el estigma social y el miedo que persiste asociado al VIH [2]. Se sigue tratando de manera diferente los casos y los enjuiciamientos de personas viviendo con VIH donde no hubo intención de causar ningún daño; donde no se produjo la transmisión del VIH o dicha transmisión era imposible o muy poco probable; y donde no existió presunta transmisión ni se demostró una [1,3]. Preocupados por como el derecho penal se aplica de una forma no coherente con las pruebas médicas y científicas actuales sobre las vías de transmisión del VIH, 20 científicos del campo del VIH, expertos en investigación científica, epidemiología y en la atención de pacientes, provenientes de diferentes regiones del mundo, elaboraron la presente Declaración de Consenso. En particular, preocupa la exageración del riesgo de transmisión del VIH y también de los posibles daños para la salud y el bienestar de las personas. Este limitado conocimiento de datos científicos actualizados sobre la ciencia del VIH refuerza el estigma, puede provocar errores judiciales y ser perjudicial para los esfuerzos destinados a hacer frente a la epidemia del VIH [4]. La Declaración de Consenso ha sido refrendada por otros científicos de todo el mundo, (Ver material suplementario S1), por la Sociedad Internacional del SIDA, la Asociación Internacional de Proveedores de Atención al Sida y el Programa Conjunto de las Naciones Unidas sobre el VIH/Sida. (Un Resumen Ejecutivo de esta Declaracion se incluyó como material suplementario S2).

El objetivo de la presente Declaración de Consenso es ayudar a científicos expertos a considerar las diferentes causas penales en las que presuntamente se haya producido un acto de no divulgación del estado serológico respecto del VIH, exposición (percibida o posible) al VIH o transmisión del VIH. Este documento ofrece opiniones formuladas por expertos acerca de las distintas dinámicas de transmisión del VIH (es decir, las “posibilidades” de transmisión), los efectos a largo plazo de la infección crónica por el VIH (es decir, el “daño”

del VIH) y el empleo de los análisis filogenéticos como pruebas. La Declaración describe las posibilidades de transmisión del VIH entre las personas que han tomado parte en una determinada situación, en un momento determinado y bajo circunstancias determinadas, aspectos en los que se suelen centrar las causas penales, y pretende divulgar las pruebas científicas que existen en la actualidad con respecto al VIH de una forma que resulte comprensible para los destinatarios ajenos al ámbito científico. (La Declaración de Consenso ha sido traducida a francés, ruso, y espanol. Ver material suplementario material S3, S4, S5).

Discusión

La primera parte de la presente Declaración se centra en las posibilidades de transmisión del VIH durante los actos concretos que se suelen tener en cuenta en los procesamientos: actividades sexuales, mordeduras o escupidas [3]. No hace referencia a otras posibles formas de transmisión del VIH, por ejemplo, recibir inyecciones, transfusiones sanguíneas o trasplantes de tejidos sin garantías de seguridad o ser objeto de procedimientos médicos que entrañen cortes o perforaciones con instrumental no esterilizado, pincharse accidentalmente con una aguja, compartir agujas, jeringuillas, soluciones de droga u otro material infectivo contaminado para consumir drogas inyectables o a través de la lactancia materna.

El contenido y la estructura de la presente Declaración de Consenso se decidió en una reunión celebrada en Seattle, EUA (febrero de 2017). Se preparó una búsqueda exhaustiva de las publicaciones disponibles en inglés hasta abril de 2017 en la base de datos en línea PubMed. Se utilizaron términos relacionados con las posibilidades de transmisión del VIH, como “VIH y carga viral”, “riesgo de transmisión del VIH por cada acto sexual”, “transmisión del VIH por sexo oral”, “transmisión del VIH por sexo anal”, “transmisión del VIH por cada coito vaginal preservativo”, “transmisión del VIH por cada sexo anal preservativo” y “transmisión del VIH por cada sexo anal circuncisión”. Se utilizaron artículos clave para buscar artículos relacionados. Se dio preferencia a los metaanálisis, los exámenes y los estudios importantes. Los autores identificaron otras fuentes y cuando resultó conveniente, se emplearon resúmenes de conferencias científicas.

Los autores participaron en varias rondas de redacción y revisión del texto de la Declaración, teniendo en cuenta la calidad de la información disponible en investigaciones científicas y médicas según la siguiente jerarquía: examen sistemático de ensayos clínicos aleatorios; ensayos clínicos aleatorios; y estudios comparativos (es decir, estudios de cohortes, estudios de casos y testigos, y estudios de control histórico). Se llevaron a cabo dos teleconferencias

para debatir acerca del proyecto preliminar y, a continuación, se completaron tres rondas de reformulación a través de la correspondencia electrónica entre todos los autores. Se realizaron consultas con expertos jurídicos en el plano nacional e internacional, por ejemplo, con el personal del Programa Conjunto de las Naciones Unidas sobre el VIH/Sida (ONUSIDA), acerca de la aplicación del derecho penal en los casos relacionados con el VIH. Se convocó una segunda reunión presencial en París (Julio de 2017) para resolver las cuestiones pendientes sobre el análisis de los datos. Los autores llevaron a cabo otras rondas de comentarios y reformulación hasta estar de acuerdo en que la Declaración de Consenso presentaba con precisión las investigaciones científicas actuales en el campo de la transmisión, los daños de la infección por el VIH, y la utilización de evidencia científica en los tribunales.

Los autores tuvieron en cuenta las conclusiones numéricas y las estimaciones estadísticas de todos los estudios mencionados en el presente documento, incluyendo los resúmenes de datos de los informes presentados de manera sistemática o en formato de tablas (por ejemplo, los trabajos de Patel *et al.* [5]). El tipo y la calidad de las pruebas en que se basan las estimaciones relativas a las posibilidades de transmisión del VIH en función de los distintos actos son variables; los autores tuvieron en cuenta estas consideraciones al evaluar las probabilidades de transmisión vinculadas a los distintos actos. Los autores dividieron las pruebas vinculadas a la transmisión por diferentes actos en tres categorías (tabla 1).

Tabla 1: Escala de calidad para las pruebas relacionadas con las posibilidades de transmisión del VIH

Actos para los que es posible calcular las probabilidades de transmisión con cierto grado de certidumbre porque se han realizado varios estudios de cohortes (p. ej., actos como coitos vaginales o anales).
Actos para los que es posible calcular las probabilidades de transmisión con menor grado de certidumbre a partir de estudios de casos aislados, verosimilitud biológica o modelos matemáticos (p. ej., actos como el sexo oral o la transmisión por medio de líquido preeyaculatorio).
Actos para los que resulta inverosímil desde una perspectiva biológica que se produzca la transmisión dado que no existen las condiciones necesarias para la transmisión (p. ej., escupidas).

Al describir las pruebas, los autores intentaron utilizar los conceptos científicos de un modo que resultase útil en el contexto del derecho penal. El concepto estadístico de los intervalos de confianza, por ejemplo, aborda la incertidumbre inherente a los resultados de la muestra de un subconjunto de una población. En el caso de las probabilidades iguales a cero o cercanas a esta cifra, los intervalos de confianza cobran una especial relevancia, ya que el hecho de que no se produzca un determinado suceso durante un estudio no constituye una prueba de que

nunca podría suceder. Cuanto mayor sea la muestra del estudio, con más precisión se podrá calcular si la probabilidad es de cero. Por consiguiente, las probabilidades de cero calculadas a partir de datos de estudios están asociadas a un intervalo de confianza que comprende del cero a una probabilidad pequeña y positiva. Es importante no malinterpretar los cálculos de los intervalos de confianza con el objeto de exagerar las posibilidades teóricas remotas.

El análisis de la metodología y los resultados de los estudios mencionados en la presente Declaración de Consenso sirvió de base para la elaboración de tres descriptores situados a lo largo de un continuo que describen las posibilidades de que se produzca la transmisión del VIH durante un solo acto concreto (tabla 2).

Tabla 2: Definición de las posibilidades de transmisión del VIH durante un solo acto concreto

**Terminología para la presente Declaración**	**Posibilidades de transmisión por cada acto**
Escasas posibilidades	La transmisión durante un único acto es posible, pero la probabilidad es baja.
Posibilidades insignificantes	La transmisión durante un único acto es extremadamente improbable, infrecuente o remota.
Ninguna posibilidad	La posibilidad de transmisión durante un único acto es biológicamente inverosímil o efectivamente nula.

Cabe destacar que la presente Declaración de Consenso no se ha concebido como un documento de salud pública que informe el desarrollo de programas y mensajes relativos a la prevención, el tratamiento y la atención del VIH. Su enfoque, basado en el nivel de riesgo individual que se puede aplicar en la justicia penal, difiere de las descripciones a nivel población que se utilizan en el contexto de la salud pública, las cuales suelen clasificar los actos sexuales desde el "riesgo reducido" hasta el "riesgo elevado". Las diferencias existentes entre los descriptores del ámbito de la salud pública y los que se utilizan en la presente Declaración de Consenso reflejan tanto los antecedentes como el contexto. En primer lugar, las definiciones del ámbito de la salud pública utilizadas para describir el riesgo de transmisión del VIH se desarrollaron durante el inicio de la epidemia del VIH, antes de que se tuviera acceso a la evidencia disponible s sobre su transmisión. En segundo lugar, describe el riesgo relativo (no el riesgo absoluto) con el fin de ayudar a las personas a reducir las posibilidades de transmisión del VIH mediante la comparación de diferentes actos.

La utilización de una terminología más simple en el área de la salud publica tenía como objetivo el desarrollo de campañas educativas para la prevención del VIH. Las categorías generalizadas conllevan actualmente auténticos problemas para la elaboración de mensajes de

promoción de la salud con relación al VIH basados en la última evidencia científica disponible [6], como es la información sobre las distintas variables que modifican el riesgo de transmisión del VIH vinculado a determinados actos, por ejemplo, con la carga viral. En algunos casos, el concepto de riesgo vinculado a ciertos actos sexuales según la caracterización utilizada en salud pública también se ha utilizado de manera incorrecta en los procesos penales, por ejemplo, en el caso canadiense de Mabior [7,8]. Por tanto, aunque la transmisión por vía sexual es un tipo habitual de transmisión del VIH en la población mundial, la presente Declaración de Consenso reconoce que las posibilidades de transmisión del VIH durante un único encuentro sexual son inexistentes o escasas, mientras que en los casos de escupidas o mordeduras no hay ninguna posibilidad o las posibilidades son insignificantes. Este enfoque científico respecto al VIH en el contexto del derecho penal es similar al que se utiliza en las declaraciones nacionales de Consenso científico de Australia [9], de Canadá [10], Suecia [11] y de Suiza [12].

Sinopsis de las posibilidades de transmisión

El VIH no se transmite con facilidad de una persona a otra. Se trata de un virus relativamente frágil que se transmite a través de vías muy bien definidas. No se transmite por el aire, por microgotas, por fómites, por vectores y no puede penetrar en piel humana intacta [13].

Para que se produzca la transmisión del VIH, deben cumplirse ciertas condiciones básicas:

- Debe haber una cantidad suficiente del virus en determinadas secreciones (sangre, semen, líquido preseminal, secreciones rectales o vaginales, o leche materna).
- Una cantidad suficiente de una de estas secreciones debe entrar en contacto directo con zonas del cuerpo de una persona VIH-negativa donde pueda comenzar la infección. Normalmente se transmite a través de membranas mucosas, tejidos dañados o úlceras inflamadas, pero no áreas de piel intacta.
- El virus debe vencer los mecanismos de defensa inmunitaria innatas de la persona para que pueda producirse y propagarse la infección.

La mayoría de las actividades cotidianas no presentan ningún riesgo de transmisión del VIH, dado que no se cumplen estas condiciones. Dejando al margen la transmisión parenteral o vertical, debe existir un contacto íntimo, como, por ejemplo, una relación sexual para que se produzca la transmisión del VIH. Incluso en estos casos la probabilidad de transmisión por cada acto es nula o escasa (las estimaciones oscilan entre el 0 y el 1,4% por acto) [5].

Factores que influyen en las posibilidades de transmisión del VIH

Las posibilidades de transmisión del VIH con relación a determinados actos varían en función de una serie de factores interrelacionados. Cuando se combinan varios factores interrelacionados, su efecto se reduce al mínimo o se amplifica en cierta medida [14].

- El uso correcto de un preservativo evita la transmisión del VIH

La correcta utilización de un preservativo (ya sea masculino o femenino) evita la transmisión del VIH dado que su porosidad protege contra los patógenos de transmisión sexual más pequeños, incluido el VIH [15]; los preservativos de látex y poliuretano actúan como una barrera física impermeable para el VIH. Un uso correcto del preservativo es aquel en el que su integridad no se ve comprometida y el preservativo se mantiene colocado durante todo el acto sexual en cuestión. Si el preservativo se utiliza correctamente durante la relación sexual, no es posible que se transmita el VIH.

Estudios de población han revelado que el uso consistente del preservativo durante las relaciones sexuales anales o vaginales reduce radicalmente la posibilidad de transmisión del VIH, incluso en los casos de uso incorrecto o de rotura [16-21]. Por ejemplo, un metaanálisis de 14 estudios reveló que el uso consistente del preservativo masculino durante las relaciones sexuales vaginales durante períodos prolongados reduce la posibilidad de transmisión del VIH en un 80% como mínimo [22]. Sin embargo, otras investigaciones más recientes sugieren que este porcentaje quizás sea mayor [23], ya que el metaanálisis empleó métodos de análisis de datos no normalizados que podrían haber dado lugar a sesgos de selección o de otro tipo y, por tanto, podrían haber reducido el nivel de prevención registrado [22,23].

Los estudios de población solo son relevantes si se producen varios actos sexuales y se desconoce si se utilizó correctamente el preservativo en cada uno de los casos. La estimación del 80% respecto a la eficacia de los preservativos a nivel de la población no existe como una estimación independiente del riesgo de transmisión del VIH, pero debe aplicarse al riesgo vinculado a los distintos actos sexuales. Por ejemplo, si el riesgo estimado de que un hombre VIH-positivo transmita el VIH a una mujer durante una única relación sexual vaginal sin preservativo es del 0,08% [5], entonces el riesgo de transmisión cuando se utiliza un preservativo se puede considerar *al menos* un 80% menor, es decir, del 0,016% (menos de 2 de cada 10.000) [5]. Cabe destacar que, cuando existen otros factores que reducen el riesgo (p. ej., una carga viral reducida o la retirada antes de la eyaculación), la posibilidad de transmisión del VIH se reduce todavía más, incluso cuando se utiliza el preservativo incorrectamente.

Es necesario reiterar que el VIH no se puede transmitir en los casos concretos en que se haya utilizado un preservativo correctamente (es decir, si se mantuvo colocado durante el acto sexual en cuestión y su integridad no se vio comprometida). Las estimaciones a nivel de la población solo se pueden aplicar a las situaciones donde se han producido múltiples casos de uso del preservativo, lo que engloba los casos puntuales de uso incorrecto y rotura.

- Una carga viral baja o “indetectable” reduce significativamente o elimina la posibilidad de transmisión del VIH

  Justo después de que una persona contraiga la infección por el VIH, su carga viral será muy elevada, pero, por lo general, disminuirá a lo largo de las primeras semanas, a medida que su sistema inmunitario responda a la infección. Si dicha persona no inicia el tratamiento, su carga viral se mantendrá bastante estable durante alguno tiempo mientras se produce el deterioro gradual del sistema inmunitario. En el caso de las infecciones por el VIH avanzadas, la carga viral suele aumentar de nuevo a niveles superiores.

  La terapia antirretroviral evita la replicación del VIH, lo que reduce considerablemente la carga viral en las secreciones de las personas. Al iniciarse una terapia antirretroviral eficaz, la carga viral suele caer hasta niveles indetectables con los análisis de sangre de laboratorio estándar existentes durante unas semanas o unos meses. La disponibilidad de los análisis y los límites mínimos de detección varían en distintas partes del mundo, de manera que dichos límites oscilan entre las 20 copias/ml y 400 copias/ml aproximadamente. Los pacientes sin progresión de la enfermedad a largo plazo son un pequeño grupo de personas que viven con el VIH, que no reciben tratamiento antirretrovirico y pueden mantener un recuento de linfocitos (células) CD4 en un rango normal por muchos años, porque su sistema inmunitario es capaz de controlar el VIH [24-28].

  Una carga viral reducida mejora la función inmunitaria y reduce radicalmente las probabilidades de que se produzca la enfermedad y el fallecimiento a largo plazo. También reduce considerablemente las posibilidades de transmitir el VIH [29-31]. La disminución de la carga viral está asociada a una reducción simultánea de las probabilidades de transmitir el VIH [32-35], lo que significa que muchas personas que están en tratamiento no pueden transmitir el virus.

En los últimos análisis de diferentes estudios clave (concretamente, HPTN052, PARTNER y Opposites Attract) en los que participaron tanto parejas heterosexuales como parejas masculinas con distintos estados serológicos con respecto al VIH, no se detectó ningún caso de transmisión sexual vinculado a una persona con una carga viral indetectable [29,30,36,37]. Estas constataciones han transformado los mensajes del campo de la salud pública. Los Centros para el Control y la Prevención de Enfermedades de los Estados Unidos, por ejemplo, ahora describen la posibilidad estimada de que una persona VIH-positiva con una carga viral indetectable (gracias a la eficacia del tratamiento antirretrovírico) transmita el VIH como un “riesgo en efecto inexistente” [6].

En 2011, el ensayo HPTN052 (llevado a cabo en Botswana, el Brasil, los Estados Unidos, la India, Kenya, Malawi, Sudáfrica, Tailandia y Zimbabwe), investigó el impacto del inicio temprano del tratamiento en1.763 personas que recibían terapia antirretroviral, tenían una carga viral estable por debajo de las 400 copias/ml, y no transmitieron el VIH. Se realizó un seguimiento de las parejas de los participantes VIH-positivos equivalente a 8.509 años-persona. Las únicas transmisiones registradas con relación a las personas en tratamiento se produjeron o bien en la fase inicial de este (antes de que la carga viral se estabilizara por debajo de las 400 copias) o cuando la carga viral era superior a las 1.000 copias/ml en dos visitas consecutivas [29,37].

Los estudios PARTNER y Opposites Attract revelaron que no existía transmisión del VIH en el caso de las personas con una carga viral inferior a las 200 copias/ml sobre la base de más de 75.000 relaciones sexuales por vía vaginal o anales sin preservativo [18,30,38]. En el estudio PARTNER, las parejas heterosexuales informaron acerca de unos 36.000 actos sexuales sin preservativo, mientras que las parejas homosexuales masculinas notificaron unos 22.000 actos sexuales sin preservativo [30]. No se produjo ninguna transmisión del VIH entre las parejas del estudio. Sí tuvieron lugar 11 infecciones nuevas por el VIH, pero, en todos los casos, los análisis filogenéticos revelaron que la causa de las infecciones había sido el contacto sexual con otra persona distinta de la pareja sexual habitual del participante. El estudio Opposites Attract englobó cerca de 17.000 actos sexuales sin preservativo entre hombres. No se registró ninguna transmisión del VIH entre las parejas que participaron en el estudio, aunque se produjeron tres infecciones nuevas por el VIH como resultado del contacto sexual con otra persona distinta de la pareja sexual habitual del participante [18].

Por otra parte, en un metaanálisis y un examen sistemático de 2013 se reportó que no existían transmisiones cuando la carga viral se encontraba por debajo de un límite de entre 50 y 500 copias/ml (en función del estudio) [39]. Otro estudio reveló que no se producían transmisiones cuando la carga viral era inferior a las 400 copias/ml [40]. Otros estudios aportaron pruebas de que una carga viral baja (aunque detectable) reduce radicalmente (y puede eliminar) las posibilidades de transmisión. Por ejemplo, en diferentes estudios anteriores donde los participantes no seguían ninguna terapia antirretroviral se registró una ausencia de transmisiones entre las parejas en las que la persona que vivía con el VIH presentaba una carga viral baja, aunque detectable: por debajo de las 1.500 copias/ml (Uganda) [32], de las 1.094 copias/ml (Tailandia) [33] y de las 1.000 copias/ml (Zambia) [34]. En el estudio ugandés, se estableció que la probabilidad de transmisión por medio de relaciones sexuales por vía vaginal cuando la carga viral era inferior a las 1.700 copias/ml era de 1 de cada 10.000 [41].

Pese a que muchos pacientes que cumplen su terapia antirretroviral presentan unas subidas temporales de poca magnitud de la carga viral conocidas como *blips* [42,43], no son indicativos de que el tratamiento del VIH esté "fallando"; se considera que no tienen trascendencia clínica; y no se ha demostrado que incrementen las posibilidades de transmisión del VIH durante las relaciones sexuales [44,45]. En los estudios a gran escala realizados con parejas con distintos estados serológicos con respecto al VIH, participaron numerosos participantes VIH-positivos que experimentaron *blips* en su carga viral en el transcurso del estudio. Por tanto, los *blips* se han tenido en cuenta en la reducción que se observó en las transmisiones.

- La profilaxis previa a la exposición (PPrE) reduce considerablemente las posibilidades de contraer el VIH
  La PPrE hace referencia al uso de medicamentos antirretrovíricos por parte de las personas VIH-negativas antes de exponerse al VIH para evitar contraer la infección [46-50]. Un estudio reciente ha revelado que la PPrE ofrece una eficacia de hasta un 95% entre las personas que cumplen el tratamiento [50]; no obstante, solo se han descrito algunos fallos de la PPrE en personas que cumplen el tratamiento, lo que sugiere que es probable que la PPrE presente una eficacia superior al 95%.

- La profilaxis posterior a la exposición (PPE) reduce significativamente las posibilidades de contraer el VIH
  La PPE hace referencia al uso por corto plazo del tratamiento antirretrovírico por parte de una persona VIH-negativa tras una exposición al VIH. Si se inicia en un plazo de 72 horas a partir de la exposición y se toma durante 28 días con una buena observancia, la PPE reduce significativamente las probabilidades de que la persona se convierta en VIH-positiva, ya que impide que el VIH se establezca en sus células inmunitarias incluso una vez que el virus está dentro del cuerpo [51-52]. A pesar de que la PPE no es 100% efectiva, se han registrado unos índices elevados de eficacia [51,53-67] (p. ej., un 81% entre los pacientes que reciben tratamientos de primera generación [67] y hasta un 100% en los pacientes que reciben tratamientos más modernos [68]). Parece que existen diversos factores que influyen en la eficacia de la PPE y, por lo general, cuanto antes se inicie la PPE y cuanto menor sea la cantidad de VIH que penetra en el cuerpo de la persona, mayor será la eficacia [68].

- La circuncisión médica masculina reduce las posibilidades de que las mujeres transmitan el VIH a los hombres
  La circuncisión médica masculina reduce las posibilidades de que las mujeres VIH-positivas transmitan el virus a los hombres VIH-negativos en un 50% aproximadamente [69]. La circuncisión también puede disminuir la transmisión sexual del VIH entre los hombres que tienen relaciones sexuales con otros hombres, en el caso de los varones VIH-negativos que adoptan el rol insertivo, pero los estudios no son concluyentes [70].

- Las prácticas de reducción de riesgos como la retirada previa a la eyaculación y la utilización de posiciones solo receptivas durante las relaciones sexuales anales **reducen** las posibilidades de transmisión del VIH.
  Algunas personas que viven con el VIH emplean prácticas de reducción de riesgos como retirarse antes de la eyaculación o adoptar posiciones estratégicas (es decir, relaciones sexuales anales solo con rol receptivo al mantener relaciones sexuales sin preservativo con una persona VIH-negativa o una persona con estado serológico desconocido. [71-73]. Este tipo de medidas reducen las posibilidades de transmisión del VIH durante las relaciones sexuales en caso de que existan dichas posibilidades [71]. Por ejemplo, un estudio realizado en 2010 reveló que las probabilidades de transmisión durante el sexo anal se redujeron aproximadamente dos tercios en los casos en que la persona VIH-positiva asumió el rol insertivo, pero no eyaculó [73].

También es sabido que las posibilidades de transmisión son menores cuando la persona VIH-positiva adopta el rol receptivo en lugar del rol insertivo durante las relaciones sexuales anales [73-75].

- Las infecciones de transmisión sexual (ITS), en determinadas circunstancias, pueden aumentar las posibilidades de que se produzca la transmisión del VIH.
  La presencia de ciertas ITS sin tratar, en particular las ITS ulcerosas, en cualquiera de los miembros de la pareja se ha asociado a un aumento de las probabilidades de transmisión del VIH durante la actividad sexual si la persona que vive con el VIH no tiene una carga viral baja [76]. Cuando los dos miembros de la pareja presentan úlceras genitales, el riesgo es aún mayor [14]. Sin embargo, la existencia de una ITS no aumenta las posibilidades de transmisión si la persona VIH-positiva sigue una terapia antirretroviral eficaz [30] *o* si la persona VIH-negativa está tomando PPrE [48,49].

Posibilidades de transmisión del VIH durante las relaciones sexuales

La transmisión sexual del VIH suele producirse cuando las secreciones con una cantidad suficiente de VIH entran en contacto con las membranas mucosas situadas en el prepucio o la uretra (en el pene); el cuello uterino o la vagina; el ano; o el recto. La transmisión del VIH también es posible a través del contacto con las membranas mucosas bucales, pero estas son mucho menos vulnerables a la transmisión del VIH [58].

Sexo oral, es decir, relaciones sexuales buco vaginales y buco peneanas

- Las posibilidades de transmisión del VIH al practicarle sexo oral a una persona VIH-positiva incluso cuando la persona no presenta una carga viral baja o no se utiliza un preservativo, son inexistentes o insignificantes en función del contexto [77,78].

Las relaciones sexuales buco genitales se promueven como una opción sexual más segura para las parejas con diferentes estados serológicos con respecto al VIH que quieren mantener relaciones sexuales íntimas y, según se informa, su práctica es muy habitual.

Es sabido que el sexo oral conlleva muchas menos posibilidades de transmisión del VIH que las relaciones sexuales vaginales o anales [79,80]. De hecho, el riesgo de transmisión del VIH vinculado al sexo oral es tan bajo que los científicos no han sido capaces de establecer una estimación contundente desde un punto de vista estadístico.

Los pocos estudios clínicos que han investigado la transmisión ligada al sexo oral no han conseguido detectar ningún caso de transmisión del VIH [74,81,82]. En un estudio realizado con parejas heterosexuales y en otro con parejas lesbianas, no se detectó ninguna transmisión como resultado de la práctica de sexo oral [81,82]. Un tercer estudio realizado con hombres que tienen relaciones sexuales con otros hombres reveló la inexistencia de seroconversiones entre los participantes que aseguraron practicar exclusivamente felaciones (con eyaculación) a hombres VIH-positivos o de estado serológico desconocido [74]. Un modelo estadístico aplicado a estas constataciones reveló que el riesgo por contacto vinculado al sexo oral era de entre 0 y 0,04% (4 de cada 10.000) [78] y, en algunos informes, se utilizan estos valores [79,80,83]. Dado que en el estudio no se detectó ninguna seroconversión, el límite superior de 0,04% se puede interpretar como un límite superior de posibilidad.

- No existe ninguna posibilidad de transmisión del VIH al practicar relaciones sexuales buco genitales a una persona VIH-positiva si esta presenta una carga viral baja **o** si se utiliza un preservativo correctamente, **y tampoco** si la persona VIH-negativa está tomando PPrE [78].

Pese a que ningún estudio ha investigado los efectos de la terapia antirretrovirica ni de la PPrE en las posibilidades de transmisión del VIH asociadas al sexo oral, según nuestra opinión como expertos, no existe ninguna posibilidad de transmisión del VIH al practicar relaciones sexuales buco genitales a una persona VIH-positiva que siga una terapia antirretrovirica, y tampoco si la persona que adopta el rol insertivo en dicha relación sexual está tomando PPrE. De la misma forma, el uso correcto de un preservativo reduce las probabilidades de transmisión del VIH a cero.

Relaciones sexuales con penetración vaginal

- Las posibilidades de transmisión del VIH asociadas a las relaciones sexuales con penetración vaginal cuando la persona VIH-positiva no presenta una carga viral reducida **y** no se utiliza preservativo es bajo [84]. Las probabilidades de transmisión disminuyen todavía más si no tiene lugar la eyaculación en el interior del cuerpo de la persona VIH-negativa.

Dos metaanálisis realizados con parejas heterosexuales [14,84] revelaron que las probabilidades de transmisión del VIH durante un único acto sexual vaginal son bajas: 0,08% (8 de cada 10.000) en ausencia de cofactores de riesgo [5,14,41,84]. No está claro si las probabilidades de que un hombre transmita el VIH a una mujer durante las relaciones sexuales vaginales son superiores a las posibilidades de que la transmisión se produzca de la

mujer al hombre. Algunos estudios no han detectado ninguna diferencia, mientras que otros indican que la probabilidad de que un hombre transmita el VIH a una mujer prácticamente duplica la probabilidad de que una mujer transmita el VIH a un hombre [14,35,83,84].

- Las posibilidades de transmisión del VIH en una relación sexual con penetración vaginal cuando la persona VIH-positiva presenta una carga viral reducida **o** utiliza un preservativo, **o** cuando la persona VIH-negativa está tomando PPrE, son inexistentes o insignificantes, dependiendo del contexto [29,38].

Como se indicó anteriormente, numerosos estudios han puesto de relieve que las probabilidades de que una persona VIH-positiva con una carga viral reducida transmita el VIH durante una relación con penetración vaginal son inexistentes o insignificantes [29,37-39,85]. En ningún ensayo clínico se ha registrado ningún caso de transmisión durante las relaciones sexuales con penetración vaginal con relación a una persona que presente una carga viral indetectable.

El VIH no se puede transmitir cuando se utiliza correctamente un preservativo, ya que el virus no puede atravesar el látex ni el poliuretano si estos se encuentran intactos. De la misma manera, no existe ninguna posibilidad de transmisión del VIH si la carga viral de la persona es indetectable.

Relaciones sexuales con penetración anal

- Las posibilidades de transmisión del VIH cuando no se utiliza preservativo **y** la persona VIH-positiva no presenta una carga viral reducida son bajas [86]. Los riesgos son similares independientemente de si la persona con rol receptivo es un hombre o una mujer. El riesgo es menor cuando la persona VIH-positiva asume el rol receptivo, en lugar del insertivo. El riesgo también es menor si la persona VIH-positiva que asume el rol insertivo no eyacula en el interior de la persona pasiva.

Los estudios revelan que el rol receptivo en las relaciones sexuales anales que se practican sin preservativo, tanto en las parejas heterosexuales o del mismo sexo está asociado a una mayor probabilidad de transmisión del VIH que el rol receptivo en las relaciones sexuales vaginales sin preservativo [5,87,88]. Los distintos estudios han arrojado estimaciones acerca de la probabilidad de transmisión del VIH por cada acto sexual anal que engloban desde el 0,01% (1 de cada 10.000) hasta más del 3% (300 de cada 10.000) [20,75,84,88-91]. La probabilidad de que la persona insertiva transmita el virus a la persona receptiva es superior que a la inversa [18,75,84].

Dos exámenes sistemáticos (2010 y 2014) arrojaron una estimación por cada acto sexual de aproximadamente un 1,4% (140 de cada 10.000) en el caso de las relaciones sexuales anales receptivos (es decir, cuando la persona VIH-positiva asume el rol insertivo) [5,86]. Un estudio de cohorte prospectivo realizado en 2010 reveló que las probabilidades caían de un 1,43% (143 de cada 10.000) cuando existía eyaculación a un 0,54% (54 de cada 10.000) en ausencia de eyaculación [91]. Según las estimaciones, las probabilidades de transmisión para cada acto sexual son de 0,11% (11 de cada 10.000) cuando la persona VIH-negativa asume el rol insertivo. [5].

- Las posibilidades de transmisión del VIH a raíz de una relación sexual con penetración anal cuando la persona VIH-positiva presenta una carga viral reducida **o** utiliza un preservativo, **o** cuando la persona VIH-negativa está tomando PPrE, son inexistentes o insignificantes, dependiendo del contexto [85,86]. La posibilidad es similar sea el receptor femenino o masculino.

Existe una posibilidad insignificante de que una persona VIH-positiva con una carga viral reducida transmita el VIH durante una relación sexual con penetración anal. Como se indicó anteriormente, en los estudios PARTNER y Opposites Attract no se detectó ninguna transmisión tras aproximadamente 39.000 actos sexuales anales practicados sin preservativo cuando la carga viral era inferior a las 200 copias/ml [30,92]. De hecho, no se ha registrado ningún caso de transmisión del VIH de una persona con una carga viral indetectable a otra, en ningún estudio clínico

El VIH no se puede transmitir cuando se utiliza correctamente un preservativo, ya que el virus no puede atravesar el látex ni el poliuretano si estos se encuentran intactos. De la misma manera, no existe ninguna posibilidad de transmisión del VIH si la carga viral de la persona es indetectable.

Posibilidades de transmisión del VIH debido al contacto ordinario, a escupidas y a mordeduras

Contacto ordinario

El VIH no se puede transmitir a través del contacto con una superficie del entorno, como una silla, un banco o un aseo; no se trasmite a través de la ingesta de comida ni de bebidas; ni con el contacto ordinario entre los seres humanos, como aquellos que se producen al abrazarse, compartir objetos domésticos o comer juntos.

El VIH no puede sobrevivir durante mucho tiempo en el aire y no es capaz de penetrar en la piel si está ilesa. No se ha detectado ningún caso de infección por el VIH vinculado al

contacto con una superficie del entorno, a la ingesta de comidas ni bebidas, ni al contacto ordinario entre las personas, pese a que muchos estudios científicos han contemplado esta posibilidad [93-98].

Mordeduras y escupidas

- No existe ninguna posibilidad de transmisión del VIH por contacto con la saliva de una persona VIH-positiva, es decir, a través de besos, mordeduras o escupidas.

Numerosos estudios han contemplado la posibilidad de que se transmita el VIH por medio de la saliva, pero ninguno ha arrojado ninguna prueba, incluido un estudio de 34.000 casos realizado en 1997 en el Reino Unido de Gran Bretaña e Irlanda del Norte [99]. El hecho de que el VIH no se transmita por la saliva se atribuye a dos factores: la saliva contiene una cantidad muy pequeña de VIH [100] y, gracias a la presencia de varios componentes inhibidores en las secreciones bucales, la saliva protege a las células vulnerables contra la infección por el VIH [101-106].

- No hay ninguna posibilidad de transmisión del VIH por medio de mordeduras o escupidas si la saliva de la persona VIH-positiva no contiene sangre o contiene una pequeña cantidad de esta.

Las pruebas actuales sugieren que el VIH ni siquiera se puede transmitir cuando la saliva contiene sangre en pequeñas cantidades. Aunque las investigaciones iniciales apuntaban hacia un riesgo teórico de transmisión en caso de que saliva que contuviese sangre penetrase en el cuerpo de una persona al entrar en contacto con el tejido mucoso (por ejemplo, al llegar a los ojos o la boca), no se ha notificado ningún caso de transmisión del VIH por escupidas que contengan sangre [107]. Por lo tanto, nuestra opinión como expertos es que no existe ninguna posibilidad de que el VIH se transmita a partir de saliva que contenga pequeñas cantidades de sangre.

- Las posibilidades de transmisión del VIH por medio de una mordedura cuando la saliva de la persona VIH-positiva contiene sangre y entra en contacto con una membrana mucosa o una herida abierta **y** la carga viral no es reducida ni indetectable son inexistentes o insignificantes.

En muchos estudios se han descrito innumerables casos en los que las mordeduras no provocaron la transmisión del VIH [108-112] o se ha determinado que la transmisión era poco probable [107,109,113,114].

Para que la transmisión sea posible en el caso de las mordeduras, la persona VIH-positiva debe tener sangre en la boca en el momento de la mordedura, debe haber una cantidad

suficiente de VIH en la sangre de la persona VIH-positiva y la mordedura debe ser lo bastante profunda como para atravesar la piel de la persona VIH-negativa y provocar traumatismos o daños tisulares [106,107,115]. Incluso cuando se cumplen todas estas condiciones, la posibilidad de transmisión por una única mordedura es, como máximo, insignificante.

Mejoras importantes en la esperanza y la calidad de vida de las personas que viven con el VIH

En la segunda sección de la presente Declaración de Consenso, se analizan los daños relacionados con el VIH debido a las persistentes concepciones erróneas que exageran los daños causados por la infección parecen influir en la aplicación del derecho penal [3]. El derecho penal tiene en cuenta los posibles daños causados por un posible delito, así como la probabilidad del hecho delictivo en sí, de manera que las definiciones de las lesiones corporales, por ejemplo, son distintas de las lesiones corporales graves, que, a su vez, se diferencian de los homicidios involuntarios o los asesinatos. Por consiguiente, es importante hacer hincapié en los enormes cambios que se han logrado en la calidad de vida de las personas que viven con el VIH a lo largo de los últimos decenios.

La evolución natural de la infección por el VIH en ausencia de tratamiento varía enormemente de una persona a otra [116]. Si no se trata, la mayoría de las personas atraviesan una etapa asintomática que se prolonga entre 2 y 15 años durante la que el virus se replica y provoca el deterioro gradual del sistema inmunitario. Un pequeño porcentaje de las personas que viven con el VIH poseen un sistema inmunitario que bloquea la replicación del virus durante un período indefinido [117], pero la gran mayoría de las personas, con el tiempo, desarrollan el sida si no reciben tratamiento (aproximadamente la mitad en un plazo de 10 años [118]). El sida se define como la presencia de determinados marcadores de laboratorio o infecciones oportunistas y enfermedades concretas que, si no se inicia la terapia antirretroviral, finalmente pueden provocar la muerte de la persona.

Las terapias antirretrovirales reducen enormemente el avance de la enfermedad asociada al VIH. A nivel mundial, se han revisado las directrices para el tratamiento con el fin de recomendar el inicio del tratamiento antirretrovírico inmediatamente después del diagnóstico de la infección por el VIH, ya que la mayoría de las personas en tratamiento lograrán tener una carga viral indetectable, mantendrán un sistema inmunitario sano, conservarán una buena salud y evitarán las complicaciones de la infección por el VIH a largo plazo [119,120]. Incluso aquellas personas que empiezan el tratamiento con una carga viral elevada y lo cumplen pueden esperar una reducción importante de la carga viral, hasta el

punto de que se produzca una recuperación significativa de su sistema inmunitario y puedan gozar así de una buena salud a largo plazo [121]. Para muchas personas, el tratamiento eficaz consiste en tomar una única pastilla al día.

Diferentes estudios realizados en numerosos países han demostrado de manera sistemática que las terapias antirretrovirales han aumentado radicalmente la esperanza de vida, que dicha esperanza de vida ha seguido mejorando con el tiempo y que la salud y la calidad de vida a largo plazo de las personas que viven con el VIH ha mejorado de forma considerable [122-141]. La esperanza de vida de los jóvenes que viven con el VIH e inician una terapia antirretroviral ya se aproxima a la de una persona joven de la población en general [45,132,134,135,137]. Además, el uso de terapias antirretrovirales han cambiado la causa de defunción de las personas que viven con el VIH de las manifestaciones clínicas del sida a distintas causas no relacionadas con el VIH [142,143] similares a las que afectan a la población general [144]. Asimismo, la atención clínica ha cambiado para incluir la gestión y el tratamiento de cuestiones de salud asociadas al envejecimiento, como la menopausia y las enfermedades cardiovasculares [143-150], y las intervenciones encaminadas a influir en las opciones de estilo de vida, como el tabaquismo [151]. En algunas subpoblaciones, una atención clínica continuada puede aumentar la esperanza de vida de las personas que viven con el VIH por encima de la de las personas VIH-negativas [135].

Aunque el VIH provoca una infección que requiere un tratamiento continuado con una terapia antirretroviral, las personas que viven con el VIH pueden gozar de una vida larga y productiva, trabajar, estudiar, viajar, mantener relaciones, tener hijos y criarlos, y contribuir a la sociedad de muchas otras formas.

Presentación de pruebas de la transmisión del VIH

En la última sección de la presente Declaración de Consenso, se reconoce la importancia del uso correcto de las pruebas científicas y médicas en los enjuiciamientos relacionados con el VIH donde se tienen en cuenta las pruebas de una transmisión real de una persona a otra.

Las directrices internacionales sobre el VIH en el contexto del derecho penal recomiendan que "la prueba de la causalidad, con relación a la transmisión del VIH, debería basarse siempre en las evidencias que se deriven de una serie de fuentes relevantes, entre ellas, historiales médicos, métodos científicos rigurosos y la historia sexual" [1].

- El historial médico puede ofrecer información contextual, pero no puede demostrar la transmisión entre la parte demandante y el acusado.

Las circunstancias del carácter y el momento de la relación sexual o las demás fuentes posibles de la infección por el VIH de una persona deben ser primordiales en los casos de supuestas transmisiones sexuales del VIH. Los historiales médicos, si están disponibles y se obtienen de una forma legítima, resultan de gran valor a la hora de identificar el último resultado negativo y el primero positivo en las pruebas del VIH de la parte demandante y el acusado. Al tener en cuenta el margen de diagnóstico de cada prueba, esta información se puede utilizar para determinar el período durante el que la parte actora contrajo el VIH y si el encausado ya era VIH-positivo en ese momento. Cabe subrayar que no se puede determinar si la parte demandante y el acusado se ha infectado primero sobre la base de quién ha obtenido primero un resultado positivo en las pruebas del VIH ni de qué persona ha presentado los cargos contra la otra.

En ocasiones, la información relacionada con la carga viral del VIH y los recuentos de CD4 que figuran en los historiales médicos se han presentado como pruebas para determinar el momento en que se produjo la infección por el VIH. Sin embargo, las cargas virales y los recuentos de CD4 presentan variaciones considerables de una persona a otra y para una misma persona, de manera que no se pueden emplear para demostrar el momento exacto en que alguien contrajo el VIH [152].

- Los análisis filogenéticos pueden utilizarse como un instrumento forense. Los resultados pueden ser compatibles con la alegación de que el acusado ha infectado a la parte demandante, pero no pueden probarlo de manera concluyente. Es importante recordar que los resultados filogenéticos pueden eximir de responsabilidad al encausado si son incompatibles con la acusación de que el encausado infectó a la parte actora.

Los análisis filogenéticos comparan la relación de la evolución del VIH de distintas personas, pero los resultados deben interpretarse con precaución en conjunto con otras pruebas fácticas y médicas cuando se utilizan en causas penales [153]. La complejidad de los análisis filogenéticos se debe, en parte, al hecho de que el VIH no es un virus de evolución rápida. El virus muta repetidamente, por lo que las personas que viven con el VIH presentan más de una variante del virus [154]. Durante la transmisión, se transmite una cantidad limitada de variantes del virus (una o algunas), pero estas también mutarán para dar lugar a variantes nuevas, de manera que el VIH de dos personas nunca es idéntico [155].

Los análisis filogenéticos del VIH incluyen la estimación de las relaciones de evolución de las variantes del VIH, por ejemplo, a fin de investigar las redes de transmisión del VIH a efectos de la salud pública. En las causas penales, los análisis filogenéticos también

investigan si la parte demandante y el acusado forman parte de la misma red de transmisión. La red se representa como un “árbol” filogenético. Concretamente, el árbol filogenético debe interpretarse como un árbol genético del VIH, el cual puede diferir del historial de transmisión porque las variantes del VIH pueden ser anteriores a la transmisión o desaparecer después de esta [156], y porque es posible que algunas personas de la red de transmisión no hayan sido diagnosticadas o no se hayan sometido a ensayos antes de elaborar el árbol.

La filogenia del VIH es muy diferente de la elaboración de perfiles de ADN, ya que, dada la evolución constante de las variantes del VIH de cada persona, la filogenia no puede obtener una “correspondencia exacta”. Cuando parece que existe una “correspondencia filogenética” entre el VIH de dos personas, esto quiere decir que dos o más variantes están “vinculadas” desde el punto de vista epidemiológico, no que sean la misma [155,157]. Las pruebas filogenéticas del VIH *pueden* exonerar de responsabilidad a un encausado acusado de transmitir el VIH a la parte actora ya que, en caso de que las cepas virales detectadas en el encausado y en la parte actora no estén relacionadas, las pruebas filogenéticas contradirán de manera concluyente la alegación de que el encausado es la fuente del virus de la parte actora [155,158].

Los últimos avances en la esfera de la filogenia y la secuenciación del ADN permiten analizar en cierta medida la dirección y el momento de la transmisión [159-162], pero actualmente estos métodos no son lo suficientemente precisos o exactos para demostrar qué persona causo la infección a la otra [155,163]. En parte, esto se debe a que siempre puede haber personas de las que no se tiene conocimiento o que no están diagnosticadas en la red de transmisión [155]. Por lo tanto, hoy en día los análisis filogenéticos no permiten descartar la posibilidad de que la parte demandante haya infectado al acusado, que ambos hayan sido infectados por un tercero [158,163] u otras situaciones de transmisión de mayor complejidad que hayan provocado que la parte demandante y el acusado tengan variantes del VIH vinculadas desde el punto de vista epidemiológico. El hecho de que tener el VIH no sirva de protección contra una “super-infección” posterior de una variante diferente incrementa la complejidad [158]. En concreto, la confianza acerca de la dirección de la infección se reduce si el acusado y la parte demandante han participado en numerosos actos sexuales que podrían haber dado lugar a múltiples casos de transmisión en distintas direcciones [155].

Los análisis filogenéticos son complejos y, por tanto, es importante que la filogenia del VIH con fines forenses y su interpretación corra a cargo de expertos que conozcan a la perfección las limitaciones de la técnica y que expliquen específicamente dichas limitaciones en sus informes escritos y sus testimonios orales. La interpretación de los resultados filogenéticos con

fines forenses requiere conocimientos especializados de filogenia y la distinción entre los árboles de evolución y los historiales de transmisión del virus. No es un proceso sencillo y todavía está pendiente la normalización de las metodologías [155]. La fiabilidad de las pruebas derivadas de los análisis filogenéticos depende de una serie de factores metodológicos, como el uso de “controles locales” adecuados [164-166] y secuencias de bases de datos [167-169] que se deben elegir utilizando criterios de selección coherentes [155]. Las investigaciones internacionales indican que las pruebas filogenéticas utilizadas en los ensayos clínicos no siempre cumplen estos requisitos [155].

Conclusión

En vista de la evidencia expuesta en el presente documento, recomendamos encarecidamente aumentar la precaución durante los procesamientos penales, en particular valorando con cuidado la evidencia científica actual sobre los daños y riesgos relacionados con el VIH. Esto es fundamental para reducir el estigma y la discriminación, así como para evitar errores judiciales.

En este contexto, esperamos que la presente Declaración de Consenso anime a los Gobiernos y a las partes que trabajan en los sistemas jurídicos y judiciales a prestar especial atención a los significativos avances que se han producido a lo largo de los tres últimos decenios en la esfera científica del VIH, y a que hagan todo lo posible para que la aplicación del derecho penal en los casos vinculados al VIH se fundamente en la comprensión correcta y completa de los conocimientos científicos actuales.

Intereses contrapuestos

Peter Godfrey-Faussett es empleado de la Escuela de Higiene y Medicina Tropical de Londres y funcionario a tiempo completo de ONUSIDA. Todos los demás autores afirman no tener intereses contrapuestos.

Agradecimientos

Agradecemos el apoyo de Sally Cameron, Edwin Bernard, Luisa Cabal, Stéphanie Claivaz-Loranger, Patrick Eba, Richard Elliott, Cécile Kazatchkine, David McLay, Kevin Osborne, Mariangela Simão y Laurel Sprague.

Financiación

El presente trabajo ha sido financiado por la Sociedad Internacional del SIDA (IAS), la Asociación Internacional de Proveedores de Atención al Sida (IAPAC), el Fondo Robert Carr para Redes de la Sociedad Civil y ONUSIDA.

Contribución de los autores

Todos los autores participaron en diferentes rondas de debate, redacción y edición de la presente Declaración de Consenso.

Referencias

1. Programa Conjunto de las Naciones Unidas sobre el VIH/Sida (ONUSIDA). Nota orientativa de ONUSIDA. *Poner fin a la penalización excesiva por la no revelación, exposición y transmisión del VIH: importantes consideraciones científicas, médicas y jurídicas*. Ginebra: ONUSIDA; 2013.
2. Comisión Mundial sobre el VIH y la Legislación. *VIH y la ley: riesgos, derechos y salud*. Ginebra: PNUD; 2012.
3. Bernard E. J. y Cameron S. *Advancing HIV Justice 2: Building momentum in global advocacy against HIV criminalization*. HIV Justice Network y Red Mundial de Personas que Viven con el VIH; abril de 2016.
4. Organización Mundial de la Salud. *Sexual health, human rights and the law*. Ginebra: Organización Mundial de la Salud; 2015.
5. Patel P., Borkowf C. B., Brooks J. T., Lasry A., Lansky A. y Mermin J. "Estimating per-act HIV transmission risk: a systematic review". *AIDS*. 19 de junio de 2014; 28(10):1509-19.
6. Centros para el Control y la Prevención de Enfermedades. "CDC Information. Undetectable Viral Load and HIV Transmission Risk". Octubre de 2017. Disponible en https://www.cdc.gov/hiv/pdf/risk/art/cdc-hiv-uvl-transmission.pdf.
7. 2008 MBQB 201; Canadá.
8. *R. v. Mabior*. SCC 47. 2012; Canadá.
9. Boyd M., Cooper D., Crock E., Crooks L., Giles M., Grulich A. *et al*. "Sexual transmission of HIV and the law: an Australian medical consensus statement". *Med J Aust*. 2016; 205(9):409-12.
10. Loutfy M., Tyndall M., Baril J.-G., Montaner J., Kaul R. y Hankins C. "Canadian consensus statement on HIV and its transmission in the context of the criminal law". *Can J Infect Dis Med Microbiol*. 2014; 25(3):135-40.
11. Albert J., Berglund T., Gisslén, M., Gröön P., Sönnerborg A., Tegnell A. *et al*. "Risk of HIV transmission from patients on antiretroviral therapy: a position statement from the Public Health Agency of Sweden and the Swedish Reference Group for Antiviral Therapy". *Scandinavian Journal of Infectious Diseases*. 2014; 46(10):673-7.
12. Vernazza P., Hirschel B., Bernasconi E. y Flepp M. "Les personnes séropositives ne souffrant d'aucune autre MST et suivant un traitment antirétroviral efficace ne transmettent pas le VIH par voie sexuelle". *Bulletin des médecins suisses*. 2008 30;89(5),165-9.
13. Vandamme A.-M., Van Laethem K., Schmit J.-C., Van Wijngaerden E., Reynders M., Debyser Z. *et al*. "Long-term stability of human immunodeficiency virus viral load and infectivity in whole blood". *European Journal of Clinical Investigation*. 1999; 29:445-52.
14. Powers K., Poole C., Pettifor A. y Cohen M. "Rethinking the heterosexual infectivity of HIV-1: a systematic review and meta-analysis". *Lancet Infect Dis*. Septiembre de 2008; 8(9):553-63.
15. Lytle C. D. "An in vitro evaluation of condoms as barriers to a small virus". *Sex Transm Dis*. 1997; 24:161-4.
16. Vittinghoff E., Douglas J., Judson F., McKirnan D., MacQueen K. y Buchbinder S. P. "Per-contact risk of human immunodeficiency virus transmission between male sexual partners". *Am J Epidemiol*. 1 de agosto de 1999; 150(3):306-11.
17. Weller S. y Davis K. "Efectividad del preservativo en la reducción de la transmisión del VIH en heterosexuales". Cochrane Database Syst Rev. [Internet]. 2001 [citado el 6 de junio de 2018] Disponible en: http://onlinelibrary.wiley.com.

18. Macdonald N., Elam G., Hickson F., Imrie J., McGarrigle C. A., Fenton K. A. *et al*. “Factors associated with HIV seroconversion in gay men in England at the start of the 21st century”. *Sex Transm Infect*. Febrero de 2008; 84(1):8-13.

19. Lavoie E., Alary M., Remis R. S., Otis J., Vincelette J., Turmel B. *et al*. “Determinants of HIV seroconversion among men who have sex with men living in a low HIV incidence population in the era of highly active antiretroviral therapies”. *Sex Transm Dis*. Enero de 2008; 35(1):25-9.

20. Scott H. M., Vittinghoff E., Irvin R., Sachdev D., Liu A., Gurwith M. *et al*. “Age, Race/Ethnicity, and Behavioral Risk Factors Associated with Per-Contact Risk of HIV Infection Among Men Who Have Sex with Men in the United States”. *J Acquir Immune Defic Syndr*. 1 de enero de 2014; 65(1): 115-121.

21. Smith D. K., Herbst J. H., Zhang X. y Rose C. E. “Condom effectiveness for HIV prevention by consistency of use among men who have sex with men in the United States”. *J Acquir Immune Defic Syndr*. 1 de marzo de 2015; 68(3):337-44. Referencia de identificador de objeto digital: 10.1097/QAI.0000000000000461.

22. Weller S. y Davis-Beaty K. “Efectividad del preservativo en la reducción de la transmisión del VIH en heterosexuales”. Cochrane Database Syst Rev 2002; (1):CD003255.

23. Crosby R. y Bounse S. “Condom effectiveness: where are we now?”. *Sex Health*. 2012; 9:10-17

24. Buchbinder S., Katz M., Hessol N., O'Malley P. y Homberg S. “Long-term HIV-1 infection without immunologic progression”. *AIDS*. 1994; 8:1123-8.

25. Madec Y., Boufassa F., Avettand-Fenoel V., Hendou S., Melard A., Boucherit S. *et al*. “Early control of HIV-1 infection in long-term nonprogressors followed since diagnosis in the ANRS SEROCO/HEMOCO cohort”. *J Acquir Immune Defic Syndr*. 2009; 50:19-26.

26. Poropatich K. y Sullivan D. J. “Human immunodeficiency virus type 1 long-term non-progressors: the viral, genetic and immunological basis for disease non-progression”. *Journal of General Virology*. 2010; 2(2):247-68.

27. Learmont J., Geczy A., Mills J., Ashton L., Raynes-Greenow C., Garsia R. *et al*. “Immunologic and virologic status after 14 to 18 years of infection with an attenuated strain of HIV-1. A report from the Sydney Blood Bank Cohort”. *N Engl J Med* 1999; 340:1715-22.

28. Rhodes D. I., Ashton L., Solomon A., Carr A., Cooper D., Kaldor J. *et al*. “Characterization of three nef-defective human immunodeficiency virus type 1 strains associated with long-term nonprogression. Australian Long-Term Nonprogressor Study Group”. *J. Virol*. Noviembre de 2000: 74(22):10581-8.

29. Cohen M. S., Chen Y. Q., McCauley M., Gamble T., Hosseinipour M. C., Kumarasamy N. *et al*. “Prevention of HIV-1 infection with early antiretroviral therapy”. *N Engl J Med*. 11 de agosto de 2011; 365:493-505.

30. Rodger A. J., Cambiano V., Bruun T., Vernazza P., Collins S., van Lunzen J. *et al*. “Sexual activity without condoms and risk of HIV transmission in serodifferent couples when the HIV-positive partner is using suppressive antiretroviral therapy”. *JAMA*. 2016; 316:171-81.

31. Montaner J. S., Hogg R., Wood E., Kerr T., Tyndall M., Levy A. R. *et al*. “The case for expanding access to highly active antiretroviral therapy to curb the growth of the HIV epidemic”. *Lancet*. 2006; 368(9534):531-6.

32. Quinn T. C., Wawer M. J., Sewankambo N., Serwadda D., Li C., Wabwire-Mangen F. *et al*. “Viral load and heterosexual transmission of human immunodeficiency virus type 1. Rakai Project Study Group”. *N Engl J Med*. 2000; 342(13):921-9.

33. Tovanabutra S., Robison V., Wongtrakul J., Sennum S., Suriyanon V., Kingkeow D. *et al*. “Male viral load and heterosexual transmission of HIV-1 subtype E in northern Thailand”. *J Acquir Immune Defic Syndr*. 1 de marzo de 2002; 29(3):275-83.

34. Fideli U. S., Allen S. A., Musonda R., Trask S., Hahn B. H., Weiss H. *et al*. "Virologic and immunologic determinants of heterosexual transmission of human immunodeficiency virus type 1 in Africa". *AIDS Res Hum Retroviruses*. 1 de julio de 2001; 17(10):901-10.

35. Hughes J. P., Baeten J. M., Lingappa J. R., Magaret A. S., Wald A. y de Bruyn G. "Determinants of per-coital-act HIV-1 infectivity among African HIV-1-serodiscordant couples". *J Infect Dis*. 1 de febrero de 2012; 205(3):358-65.

36. Grulich A., Bavinton B., Jin F., Prestage G., Zablotska, Grinsztejn B. *et al*. "HIV transmission in male serodiscordant couples in Australia, Thailand and Brazil". Resumen para la Conferencia sobre Retrovirus e Infecciones Oportunistas de 2015, Seattle (Estados Unidos), 2015.

37. Cohen M. S., Chen Y. Q., McCauley M., Gamble T., Hosseinipour M., Kumarasamy N. *et al*. "Antiretroviral Therapy for the Prevention of HIV-1 Transmission". *N Engl J Med*. 1 de septiembre de 2016; 375(9):830-9.

38. Supervie V., Viard J.-P., Costagliola D. y Breban R. "Heterosexual risk of HIV transmission per sexual act under combined antiretroviral therapy: systematic review and Bayesian modeling". *Clin Infect Dis*, publicación oficial de la Infect Dis Soc Am. 1 de julio de 2014; 59(1):115-22.

39. Loutfy M. R., Wu W., Letchumanan L., Bondy L., Antoniou T., Margolese S. *et al*. "Systematic Review of HIV Transmission between Heterosexual Serodiscordant Couples where the HIV Positive Partner Is Fully Suppressed on Antiretroviral Therapy". *PLoS ONE*. 13 de febrero de 2012; 8(12).

40. Anglemyer A., Rutherford G. W., Baggaley R. C., Egger M. y Siegfried N. "Tratamiento antirretroviral para la prevención de la transmisión del VIH en parejas serodiscordantes". Cochrane Database Syst Rev. 30 de abril de 2013; (4).

41. Mastro T. D. y de Vincenzi I. "Probabilities of sexual HIV-1 transmission". *AIDS*.1996.10 supl. A:S75-82.

42. Gray R. H., Wawer M. J., Brookmeyer R., Sewankambo N. K., Serwadda D., Wabwire-Mangen F. *et al*. "Probability of HIV-1 transmission per coital act in monogamous, heterosexual, HIV-1-discordant couples in Rakai, Uganda". *Lancet*. 14 de abril de 2001; 357(9263):1149-53.

43. Young J., Rickenbach M., Calmy A., Bernasconi E., Staehelin C., Schmid P. *et al*. "Transient detectable viremia and the risk of viral rebound in patients from the Swiss HIV Cohort Study". *BMC Infect Dis*. 2015; 15(1):382.

44. Sörstedt E., Nilsson S., Blaxhult A., Gisslén M., Flamholc L., Sönnerborg A. *et al*. "Viral blips during suppressive antiretroviral treatment are associated with high baseline HIV-1 RNA levels". *BMC Infectious Diseases*. 2016; 16:305.

45. Van Sighem A., Zhang S., Reiss P., Gras L., van der Ende M., Kroon F. *et al*. "Immunologic, virologic, and clinical consequences of episodes of transient viremia during suppressive combination antiretroviral therapy". *J Acquir Immune Defic Syndr*. 1 de mayo de 2008; 48:104-8.

46. Teira R., Vidal F., Muñoz-Sánchez P., Geijo P., Viciana P., Ribera E. *et al*. "Very low level viraemia and risk of virological failure in treated HIV-1-infected patients". *HIV Medicine*, edición en línea. *HIV Med*. Marzo de 2017; 18(3):196-203.

47. Fonner V., Dalglish S., Kennedya C., Baggaley R., O'Reilly K., Koechlinb F. *et al*. "Effectiveness and safety of oral HIV preexposure prophylaxis for all populations". *AIDS*. 2016; 30:1973-83.

48. Molina J.-M., Capitant C., Spire B., Pialoux G., Cotte L., Charreau I. *et al*. "On-Demand Preexposure Prophylaxis in Men at High Risk for HIV-1 Infection". *N Engl J Med*. 3 de diciembre de 2015; 373:2237-46.

49. McCormack, S., Dunn D., Desai M., Dolling D., Gafos M., Gilson R. *et al*. "Pre-exposure prophylaxis to prevent the acquisition of HIV-1 infection (PROUD): effectiveness results from

the pilot phase of a pragmatic open-label randomised trial". *The Lancet*. 9 de septiembre de 2015; 387(10013):53-60.

50. Grant R. M., Liegler T., Defechereux P., Kashuba A. D., Taylor D., Abdel-Mohsen M. *et al*. "Drug resistance and plasma viral RNA level after ineffective use of oral pre-exposure prophylaxis in women". *AIDS*. 2015; 29:331-7.
51. Schechter M., do Lago R., Mendelsohn A., Moreira R., Moulton L., Harrison L. *et al*. "Behavioral impact, acceptability, and HIV incidence among homosexual men with access to postexposure chemoprophylaxis for HIV". *JAIDS*. 15 de abril de 2004; 35(5):519-25.
52. Pinkerton S. D., Martin J., Roland M., Katz M., Coates T., Kahn J. *et al*. "Cost-effectiveness of postexposure prophylaxis after sexual or injection-drug use exposure to human immunodeficiency virus". *Arch Intern Med*. 2004; 164:46-54.
53. Jochimsen E. M. "Failures of zidovudine postexposure prophylaxis". *Am J Med*. 19 de mayo de 1997; 102(5) supl. 2:52-55.
54. Lot F. y Abiteboul D. "Infections professionnelles par le VIH en France chez le personnel de santé". *Bull Epi Hebdom*. 1999; 18:69-70.
55. Beltrami E. M., Luo C.-C., de la Torre N. y Cardo D. M. "Transmission of drug-resistant HIV after an occupational exposure despite postexposure prophylaxis with a combination drug regimen". *Infect Control Hosp Epidemiols*. 2002; 23:345-48, 2002.
56. Hawkins D. A., Asboe D., Barlow K. y Evans B. "Seroconversion to HIV-1 following a needlestick injury despite combination post-exposure prophylaxis". *J Infect*. 2001; 43:12-15.
57. Wulfsohn A., Venter W. D. F., Schultze D., Levey M. y Sanne I. M. "Post-exposure prophylaxis after sexual assault in South Africa". Actas de la Décima Conferencia sobre Retrovirus e Infecciones Oportunistas; febrero de 2003; Boston (Estados Unidos): resumen 42.
58. Lunding S. *et al*. "Danish postexposure prophylaxis (PEP) registry: use and failure of antiretroviral chemoprophylaxis following sexual exposure to HIV". XVI Conferencia Internacional sobre el Sida, Toronto, resumen TUPE0433, 2006.
59. Donnell D., Mimiaga M. J., Mayer K., Chesney M., Koblin B. y Coates T. "Use of non-occupational post-exposure prophylaxis does not lead to an increase in high risk sex behaviors in men who have sex with men participating in the EXPLORE trial". *AIDS Behav*. 2010; 14(5):1182-1189.
60. Sonder G. J. B., Prins J. M., Regez R. M. *et al*. "Comparison of two HIV postexposure prophylaxis regimens among men who have sex with men in Amsterdam: adverse effects do not influence compliance". *Sex Transm Dis*. 2010; 37(11):681-686.
61. McAllister J., Read P., McNulty A., Tong W. W., Ingersoll A. y Carr A. "Raltegravir-emtricitabine-tenofovir as HIV nonoccupational post-exposure prophylaxis in men who have sex with men: safety, tolerability and adherence". *HIV Med*. 2014; 15(1):13-22.
62. Jain S., Oldenburg C. E., Mimiaga M. J. y Mayer K. H. "Subsequent HIV infection among men who have sex with men who used non-occupational post-exposure prophylaxis at a Boston community health center: 1997-2013". *AIDS Patient Care STDS*. 2015; 29(1):20-25.
63. Foster R., McAllister J., Read T. R. *et al*. "Single-tablet emtricitabine-rilpivirine-tenofovir as HIV postexposure prophylaxis in men who have sex with men". *Clin Infect Dis*. 2015: 1-5.
64. Linden J. A., Oldeg P., Mehta S. D., McCabe K. K. y LaBelle C. "HIV postexposure prophylaxis in sexual assault: current practice and patient adherence to treatment recommendations in a large urban teaching hospital". *Acad Emerg Med*. 2005; 12(7):640-646.
65. Griffith W. F., Ackerman G. E., Zoellner C. L. y Sheffield J. S. "Sexual assault: a report on human immunodeficiency virus postexposure prophylaxis". *Obstet Gynecol Int*. 2010; (196963):1-6.

66. Olshen E., Hsu K., Woods E. R., Harper M., Harnisch B. y Samples C. L. “Use of human immunodeficiency virus postexposure prophylaxis in adolescent sexual assault victims”. *Arch of Pediat Adolesc Med*. 2006; 160(7):674-680.

67. Cardo D., Culver D., Ciesielski C., Srivastava P., Marcus R., Abiteboul D. *et al*. “A Case–Control Study of HIV Seroconversion in Health Care Workers after Percutaneous Exposure”. *N Engl J Med*. 20 de noviembre de 1997; 337:1485-90.

68. Poynten I. M., Smith D. E., Cooper D. A., Kaldor J. M y Grulich A. E. “The public health impact of widespread availability of nonoccupational postexposure prophylaxis against HIV”. *HIV Medicine*. Septiembre de 2007; 8(6):374-381.

69. Siegfried N., Muller M., Deeks J. J. y Volmink J. “Circuncisión masculina para la prevención de la transmisión heterosexual del VIH en hombres”. Cochrane Database Syst Rev. 15 de abril de 2009; (2): CD003362.

70. Millett G. A., Flores S. A., Marks G., Reed J. B. y Herbst J. H. “Circumcision status and risk of HIV and sexually transmitted infections among men who have sex with men: a meta-analysis”. *JAMA*. 8 de octubre de 2008; 300(14):1674-84. Fe de erratas: en *JAMA*. 18 de marzo de 2009; 301(11):1126-9.

71. Crepaz N., Marks G., Liau A., Mullins M. M., Aupont L. W., Marshall K. J. *et al*. “Prevalence of unprotected anal intercourse among HIV-diagnosed MSM in the United States: a meta-analysis”. *AIDS*. 24 de agosto de 2009; 23(13):1617-29.

72. Van De Ven P., Kippax S., Crawford J., Rawstorne P., Prestage G. Grulich A. *et al*. “In a minority of gay men, sexual risk practice indicates strategic positioning for perceived risk reduction rather than unbridled sex”. *AIDS Care*. Agosto de 2002; 14(4):471-80.

73. Jin F., Crawford J., Prestage G., Zablotska I., Imrie J., Kippax S. *et al*. “Unprotected anal intercourse, risk reduction behaviours, and subsequent HIV infection in a cohort of homosexual men”. *AIDS*. 14 de enero de 2009; 23(2):243-52.

74. Baggaley R. F., Boily M.-C., White R. G. y Alary M. “Risk of HIV-1 transmission for parenteral exposure and blood transfusion: a systematic review and meta-analysis”. *AIDS*. 2006; 20:805-12.

75. Vittinghoff E., Douglas J., Judson F., McKirnan D., MacQueen K. y Buchbinder S. P. “Per-contact risk of human immunodeficiency virus transmission between male sexual partners”. *Am J Epidemiol*. 1 de agosto de 1999; 150:306-11.

76. Sexton J., Garnett G. y Røttingen J.-A. “Metaanalysis and metaregression in interpreting study variability in the impact of sexually transmitted diseases on susceptibility to HIV infection”. *Sex Transm Dis*. Junio de 2005; 32(6):351-7.

77. Campo J., Perea M. A., del Romero J., Cano J., Hernando V. y Bascones A. “Oral transmission of HIV, ¿reality or fiction? An update”. *Oral Dis*. Mayo de 2006; 12(3):219-28.

78. Baggaley R. F., White R. G. y Boily M. C. “Systematic review of orogenital HIV 1 transmission probabilities”. *Int J Epidemiol*. Diciembre de 2008; 37(6):1255-65.

79. Morrow G., Vachot L., Vagenas P. y Robbiani M. “Current concepts of HIV transmission”. *Curr HIV/AIDS Rep*. febrero de 2007; 4(1):29-35.

80. Centros para el Control y la Prevención de Enfermedades. “Oral sex and HIV Risk. CDC HIV/AIDS Facts”, junio de 2009.

81. Del Romero J., Marincovich B., Castilla J., García S., Campo J., Hernando V. *et al*. “Evaluating the risk of HIV transmission through unprotected orogenital sex”. *AIDS*. 14 de junio de 2002; 16(9):1296-97.

82. Raiteri R., Fora R. y Sinicco A. “No HIV-1 transmission through lesbian sex”. *Lancet*. 1994; 344:270.

83. Grupo Europeo de Estudio sobre la Transmisión Heterosexual del VIH. “Comparison of female to male and male to female transmission of HIV in 563 stable couples”. *BMJ*. 28 de marzo de 1992; 304(6830):809-13.

84. Boily M.-C., Baggaley R. F., Wang L., Masse B., White R. G., Hayes R. J. *et al*. “Heterosexual risk of HIV-1 infection per sexual act: systematic review and meta-analysis of observational studies”. *Lancet Infect Dis*. Febrero de 2009; 9(2):118-29.

85. Rodger A., Cambiano V., Bruun T., Vernazza P., Collins S., Estrada V. *et al*. “HIV transmission risk through condomless sex if the HIV positive partner is on suppressive ART: PARTNER study”. Presentación de la Conferencia sobre Retrovirus e Infecciones Oportunistas, 3 a 6 de marzo de 2014; Boston (Estados Unidos).

86. Baggaley R. F., White R. G. y Boily M.-C. “HIV transmission risk through anal intercourse: systematic review, meta-analysis and implications for HIV prevention”. *Int J Epidemiol*. Agosto de 2010; 39(4):1048-63.

87. Halperin D. T., Shiboski S. C., Palefsky J. M. y Padian N. S. “High level of HIV-1 infection from anal intercourse: a neglected risk factor in heterosexual AIDS prevention”. Artículo presentado en la XIV Conferencia Internacional sobre el Sida. 2002; Barcelona (España).

88. Leynaert B., Downs A. M. y de Vincenzi I. “Heterosexual transmission of human immunodeficiency virus: variability of infectivity throughout the course of infection”. Grupo Europeo de Estudio sobre la Transmisión Heterosexual del VIH. *Am J Epidemiol*. 1 de julio de 1998; 148(1):88-96

89. Jin F., Jansson J., Law M., Prestage G. P., Zablotska I., Imrie J. C. *et al*. “Per-contact probability of HIV transmission in homosexual men in Sydney in the era of HAART”. *AIDS*. 27 de marzo de 2010; 24(6):907-13.

90. DeGruttola V., Seage G. R. 3º, Mayer K. H. y Horsburgh C. R. Jr. “Infectiousness of HIV between male homosexual partners”. *J Clin Epidemiol*.1989; 42(9):849-56.

91. Jacquez J. A., Koopman J. S., Simon C. P. y Longini I. M. Jr. “Role of the primary infection in epidemics of HIV infection in gay cohorts”. *J Acquir Immune Defic Syndr*. Noviembre de 1994; 7(11):1169-84.

92. Bavinton B., Grinsztejn B., Phanuphak N., Jin F., Zablotska I., Prestage G. *et al*. “HIV treatment prevents HIV transmission in male serodiscordant couples in Australia, Thailand and Brazil”. XIX Conferencia sobre la Ciencia del VIH de la Sociedad Internacional del SIDA, París, resumen núm. TUAC0506LB, julio de 2017.

93. Berthier A., Fauchet R., Genetet N., Fonlupt J., Genetet N., Gueguen M. *et al*. “Transmissibility of human immunodeficiency virus in haemophilic an non-haemophilic children living in a private school in France”. *Lancet*. 13 de septiembre de 1986; 2(8507):598-601.

94. Fischl M. A., Dickinson G. M., Scott G. E. *et al*. “Evaluation of heterosexual partners, children and household contacts of adults with AIDS”. *JAMA*. 1987; 257:640-4.

95. Friedland G., Kahl P., Saltzman B., Rogers M., Feiner C., Mayers M. *et al*. “Additional evidence for lack of transmission of HIV infection by close interpersonal (casual) contact”. *AIDS*. 1 de julio de 1990; 4(7):639-44.

96. Rogers M. F., White C. R., Sanders R., Schable C., Ksell T. E., Wasserman R. L. *et al*. “Lack of transmission of human immunodeficiency virus from infected children to their household contacts”. *Pediatrics*. Febrero de 1990; 85(2):210-14.

97. Courville T. M., Caldwell B. y Brunell P. “Lack of Evidence of Transmission of HIV-1 to Family Contacts of H1V-1 Infected Children”. *Clinical Pediatrics*. Marzo de 1998; 37(3):175-8.

98. “+infection among sexual and nonsexual household contacts of persons with congenital clotting disorders”. *Pediatrics*. Agosto de 1991; 88(2):242-9.

99. Gilbart V. L. "Unusual HIV transmissions through blood contact: analysis of cases reported in the United Kingdom to December 1997". *Communicable Disease and Public Health*. Junio de 1998; 1(2): 108-13.

100. Yeung S. C., Kazazi F., Randle C. G. *et al*. "Patients infected with human immunodeficiency virus type 1 have low levels of virus in saliva even in the presence of periodontal disease". *J Infect Dis*. Abril de 1993; 167(4):803-9.

101. Shine N., Konopka K. y Düzgüneş N. "The anti-HIV-1 activity associated with saliva". *J Dent Res*. Febrero de 1997; 76(2):634-640.

102. Shugars D. C., Schock D. C. y Patton J. "HIV-1 RNA load in blood plasma, saliva and crevicular fluid". *J Dent Res*. 1998; 77(número especial A):285.

103. Shugars D. C. y Wahl S. "The role of the oral environment in HIV-1 transmission". *JADA*. Julio de 1998; 129(7):851-8.

104. Malamud D. y Friedman H. M. "HIV in the oral cavity: virus, viral inhibitory activity, and antiviral antibodies". *Crit Rev Oral Biol Med*. 1993; 4(3-4):461-6.

105. Archibald D. W. y Cole G. A. "In vitro inhibition of HIV-1 infectivity by human salivas". *AIDS Res Hum Retroviruses*. Diciembre de 1990; 6(12):1425-32.

106. Yeh C. K., Handelman B., Fox P. C. y Baum B. J. "Further studies of salivary inhibition of HIV-1 infectivity". *J Acquir Immune Defic Syndr*. 1992; 5(9):898-903.

107. Cresswell F. V., Ellis J., Hartley J., Sabin C. A., Orkin C. y Churchill D. R. "A systematic review of risk of HIV transmission through biting or spitting: implications for policy". *HIV Medicine*. 23 de abril de 2018. Disponible en https://onlinelibrary.wiley.com/doi/abs/10.1111/hiv.12625.

108. Tsoukas C. *et al*. "Lack of transmission of HIV through human bites and scratches". *JAIDS*. 1988; 1(5):505-7.

109. Richman K. M. y Richman L. S. "The potential for transmission of human immunodeficiency virus through human bites". *J Acquir Immune Defic Syndr*. 1993; 64:40-46.

110. Shirley L. R. y Ross S. A. "Risk of transmission of human immunodeficiency virus by bite of an infected toddler". *The Journal of Pediatrics*. Marzo de 1989; 114(3):425-7.

111. Drummond R. "Seronegative 18 months after being bitten by a patient with AIDS". *JAMA*. 7 de noviembre de 1986; 256(17):2342-3.

112. Romea S, Alkiza M. E., Ramon J. M. y Oromí J. "Risk for occupational transmission of HIV infection among health care workers". *European Journal of Epidemiology*. Abril de 1995; 11(2):225-9.

113. Verrusio A. C. "Risk of transmission of the human immunodeficiency virus to health care workers exposed to HIV-infected patients: a review". *JADS*. Marzo de 1989; 118(3):339-42.

114. Henderson D. K., Fahey B. J., Willy M., Schmitt J. M., Carey K., Koziol D. E. *et al*. "Risk for Occupational Transmission of Human Immunodeficiency Virus Type 1 (HIV-1) Associated with Clinical Exposures: A Prospective Evaluation". *Ann Intern Med*. 15 de noviembre de 1990; 113(10):740-746.

115. Tereskerz P. M., Bentley M. y Jagger J. Risk of HIV-1 infection after human bites. *Lancet*. 30 de noviembre de 1996; 348(9040):1512.

116. Sabin C. A. y Lundgren J. D. "The natural history of HIV infection. Current Opinion in HIV and AIDS". 2013; 8(4):311-317.

117. Okulicz J. F., Marconi V. C., Landrum M. L., Wegner S., Weintrob A., Ganesan A. *et al*. "Clinical Outcomes of Elite Controllers, Viremic Controllers, and Long-Term Nonprogressors in the US Department of Defense HIV Natural History Study". *J Infect Dis*. 1 de diciembre de 2009; 200(11): 1714-23.

118. McManus H., O'Connor C. C., Boyd M., Broom J., Russell D., Watson K. *et al*. "Long-term survival in HIV positive patients with up to 15 Years of antiretroviral therapy". *PLoS One*. 7 de noviembre de 2012; 7(11).

119. Lee F. J., Amin J. y Carr A. "Efficacy of initial antiretroviral therapy for HIV-1 infection in adults: a systematic review and meta-analysis of 114 studies with up to 144 weeks' follow-up". *PLoS One*. 15 de mayo de 2014; 9(5): e97482.

120. Grupo del estudio START de INSIGHT. "Initiation of Antiretroviral Therapy in Early Asymptomatic HIV Infection". *N Engl J Med*. 27 de agosto de 2015; 373(9): 795-807.

121. Stephan C., Hill A., Sawyer W., van Delft Y. y Moecklinghoff C. "Impact of baseline HIV-1 RNA levels on initial highly active antiretroviral therapy outcome: a meta-analysis of 12,370 patients in 21 clinical trials". *HIV Med*. Mayo de 2013; 14(5):284-92.

122. Trickey A., May M., Vehreschild J.-J., Obel N., Gill M. J., Crane H. *et al*. "Survival of HIV-positive patients starting antiretroviral therapy between 1996 and 2013: a collaborative analysis of cohort studies". *The Lancet HIV*. Agosto de 2017; 4(8):e349-56.

123. Patterson S., Cescon A., Samji H., Chan K., Zhang W., Raboud J. *et al*. "Life expectancy of HIV-positive individuals on combination antiretroviral therapy in Canada". *BMC Infect Dis*. 17 de julio de 2015; 15:274.

124. Zhu H., Napravnik S., Eron J. J., Cole S. R., Ma Y., Wohl D. A. *et al*. "Decreasing excess mortality of HIV-infected patients initiating antiretroviral therapy: comparison with mortality in general population in China, 2003-2009". *J Acquir Immune Defic Syndr*. 15 de agosto de 2013; 63(5):e150-7.

125. Teeraananchai S., Kerr S. J., Amin J., Ruxrungtham K. y Law M. G. "Life expectancy of HIV-positive people after starting combination antiretroviral therapy: a meta-analysis". *HIV Med*. Abril de 2017; 18(4):256-66.

126. Lohse N. y Obel N. "Update of Survival for Persons with HIV Infection in Denmark". *Ann Intern Med*. 15 de noviembre de 2016; 165(10):749-750.

127. Price A. J., Glynn J., Chihana M., Kayuni N., Floyd S., Slaymaker E. *et al*. "Sustained 10-year gain in adult life expectancy following antiretroviral therapy roll-out in rural Malawi: July 2005 to June 2014". *Int J Epidemiol*. 2017; 46(2):479-91.

128. Nsanzimana S., Remera E., Kanters S., Chan K., Forrest J. I., Ford N. *et al*. "Life expectancy among HIV-positive patients in Rwanda: a retrospective observational cohort study". *Lancet Glob Health*. Marzo de 2015; 3(3):e169-77.

129. Johnson L. F., Mossong J., Dorrington R. E., Schomaker M., Hoffmann C. J., Keiser O. *et al*. International Epidemiologic Databases to Evaluate AIDS Southern Africa Collaboration. "Life expectancies of South African adults starting antiretroviral treatment: collaborative analysis of cohort studies". *PLoS Med*. 9 de abril de 2013; 10(4): e1001418.

130. Reniers G., Blom S., Calvert C., Martin-Onraet A., Herbst A. J., Eaton J. W. *et al*. "Trends in the burden of HIV mortality after roll-out of antiretroviral therapy in KwaZulu-Natal, South Africa: an observational community cohort study". *Lancet HIV*. Marzo de 2017; 4(3):e113-e121.

131. Gueler A., Moser A., Calmy A., Günthard H. F., Bernasconi E., Furrer H. *et al*. "Life expectancy in HIV-positive persons in Switzerland: matched comparison with general population". *AIDS*. 28 de enero de 2017; 31(3):427-36.

132. Teeraananchai S., Chaivooth S., Kerr S. J., Bhakeecheep S., Avihingsanon A., Teeraratkul A., Sirinirund P., Law M. G. y Ruxrungtham K. "Life expectancy after initiation of combination antiretroviral therapy in Thailand". *Antivir Ther*. 5 de enero de 2017.

133. Asiki G., Reniers G., Newton R., Baisley K., Nakiyingi-Miiro J., Slaymaker E. *et al*. "Adult life expectancy trends in the era of antiretroviral treatment in rural Uganda (1991-2012)". *AIDS*. 28 de enero de 2016; 30(3):487-93.

134. May M. T., Gompels M., Delpech V., Porter K., Orkin C., Kegg S. *et al*. "Impact on life expectancy of HIV-1 positive individuals of CD4+ cell count and viral load response to antiretroviral therapy". *AIDS*. 15 de mayo de 2014; 28(8):1193-202.

135. Samji H., Cescon A., Hogg R. S., Modur S. P., Althoff K. N., Buchacz K. *et al*. "Closing the gap: increases in life expectancy among treated HIV-positive individuals in the United States and Canada". *PLoS One*. 18 de diciembre de 2013; 8(12):e81355.

136. Siddiqi A. E., Hall H. I., Hu X. y Song R. "Population-Based Estimates of Life Expectancy After HIV Diagnosis: United States 2008-2011". *J Acquir Immune Defic Syndr*. 1 de junio de 2016; 72(2):230-6.

137. Marcus J. L., Chao C. R., Leyden W. A., Xu L., Quesenberry C. P. Jr., Klein D. B. *et al*. "Narrowing the Gap in Life Expectancy Between HIV-Infected and HIV-Uninfected Individuals With Access to Care". *J Acquir Immune Defic Syndr*. 1 de septiembre de 2016; 73(1):39-46.

138. Furuya-Kanamori L., Kelly M. D. y McKenzie S. J. "Co-morbidity, ageing and predicted mortality in antiretroviral treated Australian men: a quantitative analysis". *PLoS One*. 25 de octubre de 2013; 8(10): e78403.

139. van Sighem A. I., Gras L. A., Reiss P., Brinkman K. y de Wolf F. "Life expectancy of recently diagnosed asymptomatic HIV-infected patients approaches that of uninfected individuals". *AIDS*. 19 de junio de 2010; 24(10):1527-35.

140. Nglazi M., West S., Dave J., Levitt N. y Lambert E. "Quality of life in individuals living with HIV/AIDS attending a public sector antiretroviral service in Cape Town, South Africa". *BMC Public Health*. 3 de julio de 2014; 14:676.

141. Wandeler G., Johnson L. F. y Egger M. "Trends in life expectancy of HIV-positive adults on antiretroviral therapy across the globe: comparisons with general population". *Curr Opin HIV AIDS*. Septiembre de 2016; 11(5):492-500.

142. Palella F. J. Jr., Baker R. K., Moorman A. C., Chmiel J. S., Wood K. C., Brooks J. T. *et al*. "Mortality in the highly active antiretroviral therapy era: changing causes of death and disease in the HIV outpatient study". *J Acquir Immune Defic Syndr*. Septiembre de 2006; 43(1):27-34.

143. Crum N. F., Riffenburgh R. H., Wegner S., Agan B. K., Tasker S. A., Spooner K. M. *et al*. "Comparisons of causes of death and mortality rates among HIV-infected persons: analysis of the pre-, early, and late HAART (highly active antiretroviral therapy) eras". *J Acquir Immune Defic Syndr*. 1 de febrero de 2006; 41(2):194-200.

144. Deeks S. G. y Phillips A. N. "HIV infection, antiretroviral treatment, ageing, and non-AIDS related morbidity". *BMJ*. 31 de enero de 2009; 338:288-92.

145. Kojic E. M., Wang C. C. y Cu-Uvin S. (2007). "HIV and menopause: a review". *J Womens Health (Larchmt)*. Diciembre; 16(10):1402-11.

146. Manfredi R. "HIV disease and advanced age: an increasing therapeutic challenge". *Drugs Aging*. 2002; 19(9):647-69.

147. Manfredi R. "HIV infection and advanced age emerging epidemiological, clinical, and management issues". *Ageing Res Rev*. enero de 2004; 3(1):31-54.

148. Serrano-Villar S., Gutiérrez F., Miralles C., Berenguer J., Rivero A., Martínez E. *et al*. "Human Immunodeficiency Virus as a Chronic Disease: Evaluation and Management of Nonacquired Immune Deficiency Syndrome-Defining Conditions". *Open Forum Infect Dis*. 12 de mayo de 2016; 3(2):ofw097.

149. Narayan K. M., Miotti P. G., Anand N. P., Kline L. M., Harmston C., Gulakowski R. 3.º *et al*. "HIV and noncommunicable disease comorbidities in the era of antiretroviral therapy: a vital agenda for research in low- and middle-income country settings". *J Acquir Immune Defic Syndr*. 1 de septiembre de 2014; 67 supl. 1: S2-7.

150. Bloomfield G. S., Khazanie P., Morris A., Rabadán-Diehl C., Benjamin L. A., Murdoch D. *et al*. "HIV and noncommunicable cardiovascular and pulmonary diseases in low- and middle-income countries in the ART era: what we know and best directions for future research". *J Acquir Immune Defic Syndr*. 1 de septiembre de 2014; 67 supl. 1: S40-53.

151. Helleberg M., Afzal S., Kronborg G., Larsen C. S., Pedersen G., Pedersen C. *et al*. "Mortality Attributable to Smoking Among HIV-1-Infected Individuals: A Nationwide, Population-Based Cohort Study". *Clin Infect Dis*. Marzo de 2013; 56(5):727-34.

152. Rodríguez B., Sethi A. K., Cheruvu V. K., Mackay W., Bosch R. J., Kitahata M. *et al*. "Predictive value of plasma HIV RNA level on rate of CD4 T-cell decline in untreated HIV infection". *Journal of the American Medical Association*, 27 de septiembre de 2006; 296(12):1498-1506.

153. Bernard E., Azad Y., Delpech V. y Geretti A. M. "HIV Forensics II: Estimating the likelihood of recent HIV infection: Implications for criminal prosecution". Fondo Fiduciario Nacional para el Sida del Reino Unido. Londres. Julio de 2011.

154. Goodenow M., Huet T., Saurin W., Kwok S., Sninsky J. y Wain-Hobson S. "HIV-1 isolates are rapidly evolving quasispecies: evidence for viral mixtures and preferred nucleotide substitutions". *J Acquir Immune Defic Syndr*. 1989; 2:344-352.

155. Abecasis A., Pingarilho M. y Vandamme A.-M. "Phylogenetic analysis as a forensic tool in HIV transmission investigations: a literature review". *AIDS*. 26 de diciembre de 2017; 31, 2017.

156. Romero-Severson E., Skar H., Bulla I., Albert J. y Leitner T. "Timing and order of transmission events is not directly reflected in a pathogen phylogeny". *Mol Biol Evol*. 2014; 31:2472-2482.

157. Rambaut A., Posada D., Crandall K. A. y Holmes E. C. "The causes and consequences of HIV evolution". *Nat Rev Genet*. 2004; 5:52-61.

158. Bernard E. J., Azad Y., Vandamme A. M., Weait M. y Geretti A. M. "HIV forensics: pitfalls and acceptable standards in the use of phylogenetic analysis as evidence in criminal investigations of HIV transmission". *HIV Med*. Septiembre de 2007; 8(6):382-387.

159. Romero-Severson E. O., Bulla I. y Leitner T. "Phylogenetically resolving epidemiologic linkage". *Proc Natl Acad Sci USA*. 8 de marzo de 2016; 113(10):2690-5.

160. Huelsenbeck J. y Hillis D. M. "Success of phylogenetic methods in the four-taxon case". *Syst Biol*. 1993; 247-264.

161. Wertheim J. O., Sanderson M. J., Worobey M. y Bjork A. "Relaxed molecular clocks, the bias-variance trade-off, and the quality of phylogenetic inference". *Syst Biol*. 2010; 59:1-8.

162. González-Candelas F., Bracho M. A., Wróbel B. y Moya A. "Molecular evolution in court: analysis of a large hepatitis C virus outbreak from an evolving source". *BMC Biol*. 2013; 11:76.

163. Abecasis A. B., Geretti A. M., Albert J., Power L., Weait M. y Vandamme A.-M. "Science in court: the myth of HIV fingerprinting". *Lancet Infect Dis*. Febrero de 2011; 11(2):78-9.

164. Ou C. Y., Ciesielski C. A., Myers G., Bandea C. I., Luo C. C., Korber B. T. *et al*. "Molecular epidemiology of HIV transmission in a dental practice". *Science*. 1992; 256:1165-1171.

165. Smith T. F. y Waterman M. S. "The continuing case of the Florida dentist". *Science*. 1992; 256:1155-1156.

166. DeBry R. W., Abele L. G., Weiss S. H., Hill M. D., Bouzas M., Lorenzo E. *et al*. "Dental HIV transmission?" *Nature*. 1993; 361:691.

167. Lemey P., Van Dooren S., Van Laethem K., Schrooten Y., Derdelinckx I., Goubau P. *et al*. "Molecular testing of multiple HIV-1 transmissions in a criminal case". *AIDS Lond Engl*. 2005; 19:1649-1658.

168. Goedhals D., Rossouw I., Hallbauer U., Mamabolo M. y de Oliveira T. "The tainted milk of human kindness". *Lancet Lond Engl*. 2012; 380:702.

169. Paraschiv S., Banica L., Nicolae I., Niculescu I., Abagiu A., Jipa R. *et al*. "Epidemic dispersion of HIV and HCV in a population of co-infected Romanian injecting drug users". *PloS One*. 2017; 12.
